# Evaluation of phytoconstituents in marigold effluent for their antifungal activity against plant pathogens

**DOI:** 10.3389/ffunb.2024.1345543

**Published:** 2024-04-04

**Authors:** Tulja Sanam, Umashankar Nagaraju, Benherlal P. S, Sridhar Goud Nerella, Jayaramaiah. R, Kadalli. G. G, Satya Srii. V

**Affiliations:** ^1^ Department of Agricultural Microbiology, University of Agricultural Sciences, Bangalore, India; ^2^ Department of Plant Biotechnology, University of Agricultural Sciences, Bangalore, India; ^3^ Department of Medicinal Chemistry, National Institute of Pharmaceutical Education and Research (NIPER), Hyderabad, India; ^4^ Department of Agronomy, University of Agricultural Sciences, Bangalore, India; ^5^ Department of Soil Science and Analytical Chemistry, University of Agricultural Sciences, Bangalore, India; ^6^ Department of Seed Science and Technology, University of Agricultural Sciences, Bangalore, India

**Keywords:** marigold (*Tagetes erecta* L.) effluent, phytoconstituents, GC-MS, principal component analysis, organic compounds, antifungal activity, plant pathogens

## Abstract

The current study placed an intense emphasis on the excess discharge of agro-based industrial effluent and the use of plant extract antimicrobials to inhibit the growth of pathogens in crop plants. An effluent (treated and untreated) from the marigold flower processing industry has been identified for the presence of volatile and semi-volatile organic compounds, and a total of 18 in treated effluent and 23 in untreated effluent were found using gas chromatography–mass spectrometry. A total of 13 classes were identified, which include carboxylic acid, phenols, esters, alkanes, alkenes, alcohols, cyanide, heterocyclic, flavonoids, aldehydes, polycyclic aromatic, cycloalkanes, and cycloalkenes. A principal component analysis with varimax rotation was applied to discern the abundance of identified compounds under each class. An *in vitro* antifungal bioassay was conducted using effluents at three different concentrations against plant pathogens (*Alternaria alter nata*, *Sclerotium rolfsii*, *Rhizoctonia solani*, *Pythium aphanidermata*, *Fusarium oxysporum*, and *Colletotrichum gloeosporioides*). The study proved that treated and untreated effluents clearly inhibited the growth of fungal pathogens by 10 to 32% and 37 to 92%, respectively. The findings suggest that marigold flower effluent can be a promising resource for developing new plant protection methods that are effective against pathogenic fungi.

## 1 Introduction

The ecosystem is being severely harmed by the indiscriminate discharge of treated and untreated industrial effluents into waterbodies or for agricultural use. Before being discharged and utilized, this constraint requires thought. Prior to any further usage, it is important to concentrate on the material elements because it is well known that volatile and semi-volatile compounds are the primary constructors or destroyers of the ecosystem (Wilde et al., 2020). Numerous researchers are studying the organic constituents in air (Li et al., 2022), food (He et al., 2022), and water (Ghorbani et al., 2022). However, there is a lack of information on the characterization of the organic fraction of industrial effluents, especially agro-industry effluents that may have many phytoconstituents with beneficial properties. The identification of the unidentified analytes from the complex mixtures of the organic compounds in effluents mainly relies on matching the registered mass spectra with the spectra in a reference library (Gao et al., 2004). Using a single identifying variable is inadequate for the results to be considered trustworthy. The integration of both the GC and MS identification approaches significantly improves the reliability of GC/MS detection (Babushok et al., 2007).

In the present study, an analysis of agro-industry effluent for its volatile and semi-volatile compounds was carried out, and there was an attempt to find its potential role in promoting antimicrobial activity. Effluent was collected from the marigold flower processing industry located at Karnataka, India, which deals with the processing of the marigold flower (*Tagetes erecta* L.). The flower petals were mainly used for the extraction of pigments (**Supplementary Figure S1**). During the process of extraction, around 4 to 6 (105–158 k) gallons lakh litres of wastewater is oozed out from the flower, which further undergoes wastewater treatment, and the treated marigold flower effluent (TMFE) is let into waterbodies. In this study, the water before treatment and after treatment (**Supplementary Figure S2**) is collected and analyzed for its organic constituents, as it is an agro-industry waste and may contain beneficial bioactive compounds. Previous studies on the volatile substances in the flowers and leaves of marigold have indicated that there is a large proportion of various terpenoids, which produce the terpenes volatile organic compounds (VOCs) (Ogunwande and Olawore, 2006). Few flavone derivatives have been identified in different marigold species, namely, luteolin and luteolin 7-O-glucoside from *Tagetes multiflora* (Abdala, 2003), and chrysocriol-7-O-(6-O-β-L-arabinofuranosyl)-D-glucopyranoside from *Tagetes patula* (Kurkina et al., 2021). Similarly, carotenoids, mainly β-carotene and zeaxanthin, were found in Tagetes species (Breithaupt et al., 2001). Phenolic derivatives such as syringic acid, 2,3,4-dihy-droxybenzonicacid, gallic acid, and n-hexadecane were found in *T*. *erecta* (Huang et al., 2007). Phenylpropanoids, namely caffeic acid-O-glucoside and rosmarinic acid, from *T*. *maxima* (Parejo et al., 2004) were reported. The above-mentioned compounds, in addition to essential oils (Salehi et al., 2018), Terpenoids, Palmitin, and Alkaloids, were found in *T*. *erecta* (Xu et al., 2011). Direct plant extracts as well as the effluents from plant processing industries possess volatile and semi-volatile compounds, namely effluent from pulp and paper mills. An agro-industry effluent was studied for the presence of compounds, namely phenol, 2-methoxy-4-(1-propenyl) (6.33%), phenol, hexadecenoic acid (5.1%), ethanone,1-(3-methoxyphenyl) (5.09%), ergosta-4, 6, 22-trien-3, beta-ol (4.38%), and beta-sitosterol (4.05%) (Ligon et al., 2008).

In addition to effluent and plant extracts containing organic compounds, earlier research has shown conclusively that plants and plant-based components that contain organic compounds play a significant role in antimicrobial activity (Gakuubi et al., 2017; Siddiqui et al., 2017). Many plants possess bioactive compounds, including tannins, alkaloids, phenolic chemicals, and flavonoids, which have been shown to have antibacterial activities *in vitro* (Duraipandiyan et al., 2006; Djeussi et al., 2013; Samuel et al., 2013). The marigold flower of the *T. patula* (Faizi et al., 2008) and *T. erecta* (Perisoara et al., 2022) species’ extracts were also reported to have antibacterial and antifungal activity (Mares et al., 2002). A study aimed to evaluate the antibacterial and antifungal activities of extracts from different parts of *T. patula*, *T. erecta*, and *T. minuta*, and obtained a positive result against few Gram-positive bacteria and Gram-negative bacteria (Latifian et al., 2021).

Considering the previous findings pertaining to plant extracts, especially the flower part possessing organic compounds and antimicrobial properties, the chemical composition of the marigold flower effluent (MFE) has been identified using GC-MS, and the antifungal activity of the marigold flower effluent from the agricultural produce processing industry is evaluated. To determine the degree of inhibition with concentration change, an *in vitro* antifungal plate bioassay using the ethyl acetate fraction against plant pathogens was designed. A principal component analysis (PCA) with varimax rotation was plotted to elucidate the abundance of compounds under each class and to quantify the compounds separately under treated and untreated marigold flower effluent (UMFE).

Recent studies have focused intensively on the discovery of new antimicrobial compounds from a wide variety of plants (Chootip et al., 2017) in relation to human disease-causing microbes. It has also been observed that there is an increased interest in the development of sustainable antifungal agents, such as plant-based essential oils and extracts, to combat plant diseases (Costa et al., 2000; Rahman et al., 2010). Many plant species have been tested for their antimicrobial properties, but most of them have not been adequately evaluated. Studies in the literature of *Tagetes* sp. have proven the efficacy of the species in having beneficial bioactive compounds with antimicrobial properties (Igwaran et al., 2017). There are very few studies pertaining to the use of plant extracts or agro-industry effluents as an antifungal agent, especially for plant pathogens. Using extracts or effluents possessing phytoconstituents as an antimicrobial agent protects plants from disease-causing pathogens and reduces pesticide toxicity in soil. Henceforth, in the present study, the effluent from the marigold processing industry is evaluated for its antifungal activity against plant pathogens. This study was primarily taken up to screen new alternatives to utilizing industrial waste effluent in a manner that would benefit the industry as well as farming.

## 2 Materials and methods

### 2.1 Sample collection

Marigold flower effluent (MFE) samples (both treated and untreated effluent) were collected from a marigold flower processing unit located at Hassan, Karnataka State, India. The untreated MFE is processed through flocculation, anaerobic digestion, and filtration to obtain treated MFE. Both samples were collected in clean glass bottles without leaving any air space in the bottles, and the bottles were sealed airtight, labelled, and stored at 4°C until further use. The flow chart pertaining to the processing, collection, and treatment of MFE is depicted in **Supplementary Figures S1**, **S2**.

### 2.2 Preparation of samples

The liquid–liquid partitioning method was used for the extraction of organic compounds from an aqueous of 1000 mL TMFE, and UMFE was filtered using Whatmann 1 filter paper and subjected to a concentrate form using a rotary evaporator (DLAB Digital Rotary Evaporator, Model: RE100-Pro; HCS Scientific & Chemical Pte Ltd, Penjuru Tech Hub, Singapore) at 65°C, 85 rpm under reduced pressure for the sample. It took approximately 2 to 3 h until the effluent was concentrated. An aliquot (15 mL) of a previously concentrated sample was taken into a 50 mL polypropylene screw-capped centrifuge tube. An equal volume (15 mL) of ethyl acetate (1:1, *v*/*v*) was added to the tube and vigorously homogenized in an ultrasound bath for 10 min. After phase partitioning (3 min, 4000 rpm) in a centrifuge, an aliquot of 12 mL of the organic layer was transferred to a 40 mL glass vial and concentrated by drying using vacuum centrifuge (Eppendorf, Eppendorf Vacufuge plus Vacuum Concentrator) for 30 mins, and the residue was dissolved in ethyl acetate solvent (3 mL). The obtained fractions of ethyl acetate from TMFE and UMFE were subjected to GC-MS (González-Rodríguez et al., 2009).

### 2.3 Analysis of organic compounds in the MFE using gas chromatography–mass spectrometry (GC-MS)

The ethyl acetate fraction was subjected to GC-MS analysis with the split injection in a ratio of 1: 10 in hexane using Shimadzu GC-MS QP 2010 fitted with a Shimadzu AOC-20i auto sampler, and Shimadzu Class-5000 Chromatog raphy Workstation software v 2.32 (Shimadzu, Italy) using a C_18_ nonpolar column containing a 100% dimethylpolysiloxane (PDMS) phase capillary column length of 30 m, 0.25 mm inner diameter, and 0.25 μm of film thickness (Agilent J&W CP-Sil 5 CB). GC-MS operated under an injector temperature of 260°C, column oven temperature of 75°C, flow control mode at linear velocity, and column flow of helium (99.9% purity) at 1.50 mL min^−1^. The oven temperature program was maintained at 75°C for 5 min and 320°C for 20 min, with an overall holding time of 57.21 min. The injected volume was 2.0 μL. The volatile and semi-volatile compounds were identified at an MS ionization voltage at 40 eV, ion source temperature of 140°C, and interface temperature of 240°C.

Individual components were identified by the NIST 20 MS LIB database matching system with an embedded Automated Mass Spectrometry Deconvolution and Identification System (AMDIS) and MS Interpreter for structural identification. The software calculates the Kovats Retention Index of similar compounds and identifies the unknown compound of interest (Mikaia et al., 2014). Each component percentage of area was calculated by comparing its average peak area to the total areas (**Supplementary Data**).

### 2.4 Classification of compounds and principal component analysis

The reported probable compounds were classified into different classes based on their functional groups. For better understanding, a principal component bi-plot analysis (PCA) was performed. The PCA is a standard approach for the exploration of variability in multivariate data. A variability exists among the class of compounds identified in TMFE and UMFE. The observations of possibly correlated variables were converted into a set of linearly independent variables named principal components, which were represented as functions of the original variables and considered as single observations in the function space rather than as high dimensional vectors. With the PCA transformation, the first principal component has the largest possible variance to account for, while the succeeding component in turn has the largest variance uncorrelated with those of the previous components.

In this study, the identified volatile organic compounds (VOCs) in treated and untreated effluent were used to represent the OCs profiles, and PCA application was carried out with SPSS 17.0 to determine the principal components of each profile. The data were preliminarily manipulated by calculating the unweighted average concentration of each OC species with identical units for standardization to eliminate the effects of different sizes and the unit of measurement. As the eigenvalues of the original variables were being calculated, varimax orthogonal rotation was applied to determine the principal components of observations with the identical sum of eigenvalues. The Kaiser criterion was used when determining the number of principal components (only the principal components with eigenvalues exceeding 1 were considered) (Yang et al., 2013).

### 2.5 Antifungal assay

To conduct the antifungal plate bioassay, the pure cultures of *Alternaria alternata* (ITCC 7362), *Sclerotium rolfsii* (ITCC 7421), *Rhizoctonia solani* (MTCC 9668), *Pythium aphanidermata* (MTCC 10247), Fusarium oxysporum (ITCC 6859), and *Colletotrichum gloeosporioides* (ITCC 7505) were procured from the Department of Plant Pathology, University of Agricultural Sciences, Bangalore. All six pathogens were tested for their virulence, sub-cultured on Potato Dextrose Agar (PDA) slants, and maintained at 8°C.

The inhibitory effect of TMFE and UMFE on the growth of plant pathogens at different concentrations (3 mg mL^−1^, 6 mg mL^−1^, and 9 mg mL^−1^) was tested *in vitro* by the poisoned food technique with modifications (Gakuubi et al., 2017). The marigold flower effluents were centrifuged at 5000× *g* for 15 min, the obtained organic layer was transferred to a vial and concentrated by drying using vaccum centrifuge (Eppendorf, Eppendorf Vacufuge plus Vacuum Concentrator) for 30 mins, and the residue was dissolved in ethyl acetate solvent and filter, while being sterilized using a sterile 22 μm syringe filter (Millipore). The filtrate was added to molten PDA (45°C) at three different concentrations (3 mg mL^−1^, 6 mg mL^−1^, and 9 mg mL^−1^) in a sterile Erlenmeyer flask by giving a gentle swirl to disperse the fraction in the medium. The PDA with sterile ethyl acetate fraction was dispensed into sterile petri dishes with enough care taken to avoid the trapping of air bubbles. The medium was allowed to solidify at room temperature (23 ± 2°C).

Petri plates with medium (PDA + different concentrations of TMFE and UMFE) are inoculated aseptically with fungal mycelial discs (4 mm in diameter) from 7-day-old pure culture plates using a sterile corkborer. A control PDA plate was maintained with only 1 mL of ethyl acetate to measure the effect of TMFE and UMFE on the selected fungal culture. Three replicates were maintained for each treatment, and the plates were incubated at 28°C. The fungal colony diameter readings were taken after five days of incubation. The percentage inhibition of the mycelial growth of the test fungi by TMFE and UMFE was calculated using the formula (Gakuubi et al., 2017):


$$\text{Inhibition of mycelial growth }\left(\%\right)=\;\frac{\left(dc-dt\right)}{dc}\;\times 100$$

where *dc* is the mean diameter of the colony in the control sample and dt is the mean diameter of the colony in the treated sample. The results of the bioassay were represented as the mean ± SD of three replicates in each test. An analysis of variance (two-way ANOVA) with factorial concept and type III sums of squares was performed using SPSS 2.0 software at a 95% confidence interval.

## 3 Results and discussion

### 3.1 Analysis of organic compounds in MFE by GC-MS

**Figure 1:**
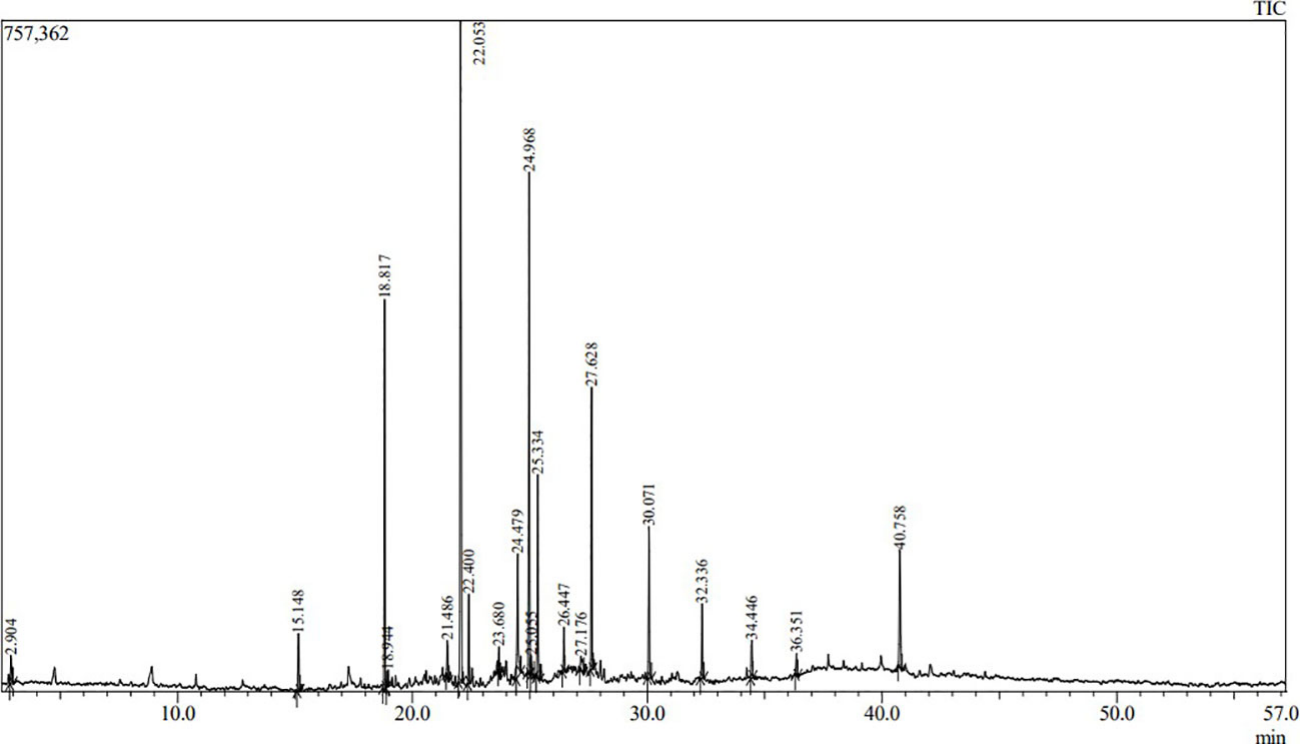
The chromatographic profile of TMFE using GC-MS.

**Figure 2:**
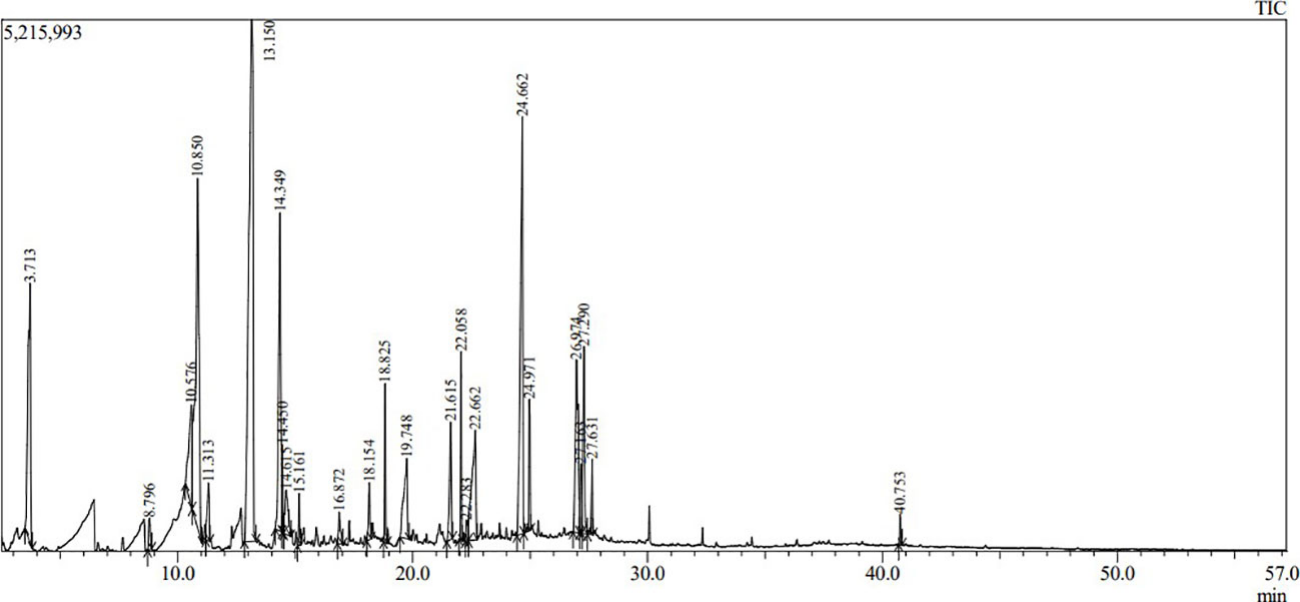
The chromatographic profile of UMFE using GC-MS.

Volatile and semi-volatile compounds were extracted and profiled in GC-MS without derivatization. The GC-MS profiling of MFE showed the presence of more than 30 different compounds (**Figures 1**, **2**). The identified compounds are listed in **Tables 1**, **2**, according to their elution order on a polysiloxane column based on the NIST library. The treated marigold flower effluent (TMFE) contains a complex mixture consisting of mainly esters, alkenes, alcohols, and carboxylic acid groups. The top five compounds in TMFE based on ionization were 1-Octadecene (21.99%), 1-Tetracosene (16.51%), 1-Nonadecene (12.37%), Octadecanal (6.81%), and 1-Hexacosene (5.37%). Similarly, a mixture of carboxylic acid, phenolic, alkenes, and alcoholic groups were noted in the untreated UMFE. The top five compounds in UMFE based on ionization were Resorcinol (24.40%), Catechol (13.02%), n-Hexadecanoic acid (10.75%), Phenol, 2,6-dimethoxy (7.59%), and 2-Furanmethanol, tetrahydro (7.55%). Few similar ester and alkene class compounds were found in the textile industry effluent (Koli et al., 2018). Having noticed more than 20 compounds, research related to the presence of organic compounds in various plant extracts has been reviewed to strengthen the present study.

**Table 1:** Organic compounds detected in TMFE by GC-MS technique.

Sl. No.	Name	Retention time	Peak		Base peak m/z	Functional group classes
			Area %	Height %		
1	1-Butanol, 3-methyl-acetate	2.90	1.16	0.98	43.05	Ester
2	Cetene	15.15	1.86	1.99	41.05	Alkene
3	1-Nonadecene	18.82	12.37	13.40	41.05	Alkene
4	Hexadecane	18.94	0.58	0.58	28.05	Alkane
5	Eicosanoic acid	21.48	1.32	1.27	43.10	Carboxylic acid
6	1-Octadecene	22.05	21.99	22.88	55.05	Alkene
7	7,9-Di-tert-butyl-1-oxaspiro(4,5)deca-6,9-diene-2,8-dione	23.68	0.64	0.87	28.05	Flavonoid (Polyphenol)
8	n-Hexadecanoic acid	24.48	5.78	4.25	28.05	Carboxylic acid
13	1-Tetracosene	24.97	16.51	17.32	43.10	Alkene
9	Carbonic acid, eicosyl prop-1-en-2-yl ester	25.05	0.94	0.70	43.10	Carboxylic acid
10	Octadecanal	25.33	6.81	7.03	43.10	Aldehyde
11	Octadecanenitrile	26.45	1.43	1.52	57.10	Cyanide
12	Octadecanoic acid	27.18	1.04	0.48	57.10	Carboxylic acid
14	1-Hexacosene	30.07	5.37	5.22	57.10	Alkene
15	Octacosanol	32.34	2.66	2.58	57.10	Alcohol
16	Octadecyl trifluoroacetate	34.45	1.33	1.30	57.10	Ester
17	Tricosyl trifluoroacetate	36.35	0.69	0.72	57.10	Ester
18	Tris (2,4-di-tert-butylphenyl) phosphate	40.76	5.14	4.06	57.10	Alcohol

**Table 2:** Organic compounds detected in UMFE by GC-MS technique.

Sl. No.	Name	Retention time	Peak		Base peak m/z	Functional group classes
			Area %	Height %		
1	2-Furanmethanol, tetrahydro-	3.71	7.55	7.07	43.10	Heterocyclic
2	Phenylethyl Alcohol	8.80	0.50	0.85	91.10	Alcohol
3	Cyclohexane carboxylic acid	10.58	3.59	2.74	55.10	Carboxylic acid
4	Catechol	10.85	13.02	9.53	110.10	Phenol (Simple Phenol)
5	Octanoic acid	11.32	1.21	1.57	110.10	Carboxylic acid
6	Resorcinol	13.15	24.40	14.22	110.10	Phenol (Simple Phenol)
7	Phenol, 2,6-dimethoxy-	14.35	7.59	8.68	154.05	Phenol (Simple Phenol)
8	Hydrocinnamic acid	14.45	0.88	2.38	91.10	Carboxylic acid
9	trans-4-Hydroxycyclohexane carboxylic acid	14.62	1.52	1.21	73.05	Carboxylic acid
10	Carbonic acid, ethyl tetradecyl ester	15.16	0.67	1.41	92.10	Ester
11	2-Ethyl-1,3-dimethyl cyclopent-2-ene carboxylic acid	16.87	0.68	0.86	43.10	Carboxylic acid
12	2-Cyclohexen-1-one, 3-(hydroxymethyl)-6-(1-methylethyl)-	18.15	0.97	1.56	98.10	Cyclo alkane
13	1-Pentadecene	18.83	1.76	4.33	43.10	Alkene
14	Benzenepropanoic acid	19.75	3.05	2.19	107.10	Carboxylic acid
15	Tetradecanoic acid	21.61	2.21	3.19	73.05	Carboxylic acid
16	7,7-Dimethoxy-2,3,4,5,6,7-hexahydro-1H-cyclopenta[a]pentalene	22.28	0.49	0.57	198.00	Cyclo alkene
17	Benzoic acid, 4-hydroxy-3,5-dimethoxy-	22.66	4.76	2.99	198.00	Carboxylic acid
18	n-Hexadecanoic acid	24.66	10.75	11.36	73.05	Carboxylic acid
19	Oleic Acid	26.97	5.31	4.70	55.05	Carboxylic acid
20	7-Acetyl-3,6-dihydroxy-8-methyl-3,4-dihydro-2H-naphthalen-1-one	27.16	1.11	1.89	43.05	Polycyclic aromatic
21	Octadecanoic acid	27.29	3.30	5.10	43.10	Carboxylic acid
22	1-Nonadecene	27.63	0.80	2.06	97.15	Alkene
23	Tris (2,4-di-tert-butylphenyl) phosphate	40.75	0.37	0.83	57.10	Alcohol

Most of the plant extracts contain bioactive compounds (Mohy El-Din and Mohyeldin, 2018), and one of the studies provide strong evidence of the presence of polyphenols, such as caffeic acid, gallic acid, acylated flavonoid-O-glycosides, and methoxylated flavonoids, in the marigold extract (De Rijke et al., 2006).

The volatile organic compounds identified in MFE were grouped into 13 different classes based on their functional groups, namely carboxylic acid, phenolic group, esters, alkanes, alkenes, alcohols, cyanide, heterocyclic groups, flavonoids, aldehydes, polycyclic aromatic, cyclo alkanes, and cyclo alkenes. Hence, from this we can infer that most of the agro-based industry effluents possess similar kinds of organic compounds (Djeussi et al., 2013). To better understand the volatile organic compounds distribution in MFE, a principal component analysis (PCA) was carried out using the recorded dataset. PCA with varimax rotation was applied to elucidate the presence of compounds under each class (Zhong et al., 2011).

**Figure 3:**
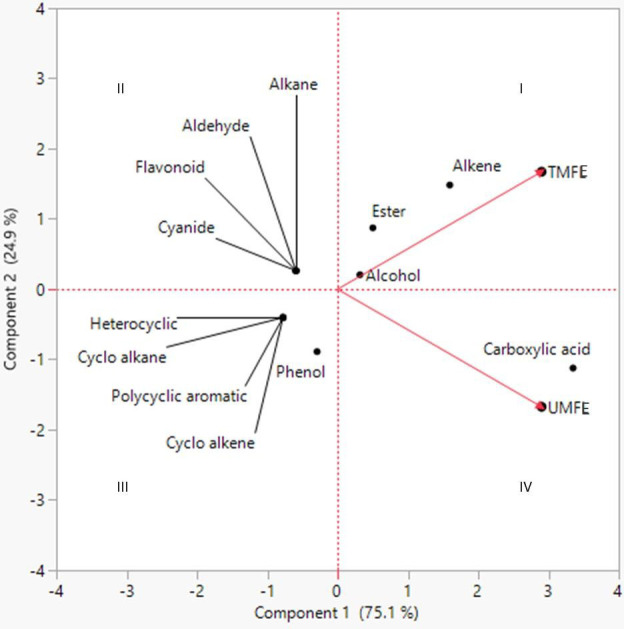
Principal component analysis (PCA) of different classes of volatile and semi-volatile or ganic compounds as evaluated in TMFE and UMFE.

The two-dimensional PCA biplot was generated based on principal components (scores) of 18 and 24 organic compounds identified in TMFE and UMFE, respectively. The scores, scoreplot, eigenvalues and covariance matrix of components is presented in the **Supplementary Data**. The 2-D PCA biplot showed projections of the variables in the factors space of Component 1 (75.1%) and Component 2 (24.9%), and together explained 100% of the total variations in the dataset (**Figure 3**). The responses of the TMFE and UMFE to the presence of organic compounds differed; hence, the separation of their locations on the 2-D PCA biplot is observed. However, the organic compounds of the MFE formed a cluster, and the individual datasets were separated based on the variations in their number and quantity of compounds noticed in the MFE. The variation in the presence of organic compounds due to a difference in effluent quality is clearly represented on the PCA plot.

Firstly, the compounds belonging to classes alkenes, esters, alcohol, and carboxylic acid were grouped and located in quadrants Q_1_ and Q_4_, indicating their presence in both TMFE and UMFE. However, higher carboxylic acid class compounds were found in UMFE, whereas esters and alkenes were found in TMFE. It was observed that alcohols located at origin represents, an equidistributional of compounds belonging to this class in TMFE as well as UMFE. The classes located in quadrant Q_2_ belong to TMFE only, and the classes in quadrant Q_3_ were reported to belong only to UMFE. It was even noticed that there was a higher presence of phenolic class compounds in UMFE than heterocyclic, polycyclic aromatic, cycloalkanes, and cycloalkene groups of compounds. A similar kind of data interpretation was made for the analysis of the metabolites in vegetable plants on treatment with municipal solid waste compost (Abbey et al., 2021). The difference in the composition of compounds in treated and untreated effluent is mainly because the untreated effluent is a crude extract from marigold flowers, whereas treated effluent is obtained after treating the crude effluent by removing the solid particles using different sized filters, followed by the primary and secondary treatment of effluent using flocculation and flotation methods.

### 3.2 Antifungal bioassay

**Figure 4:**
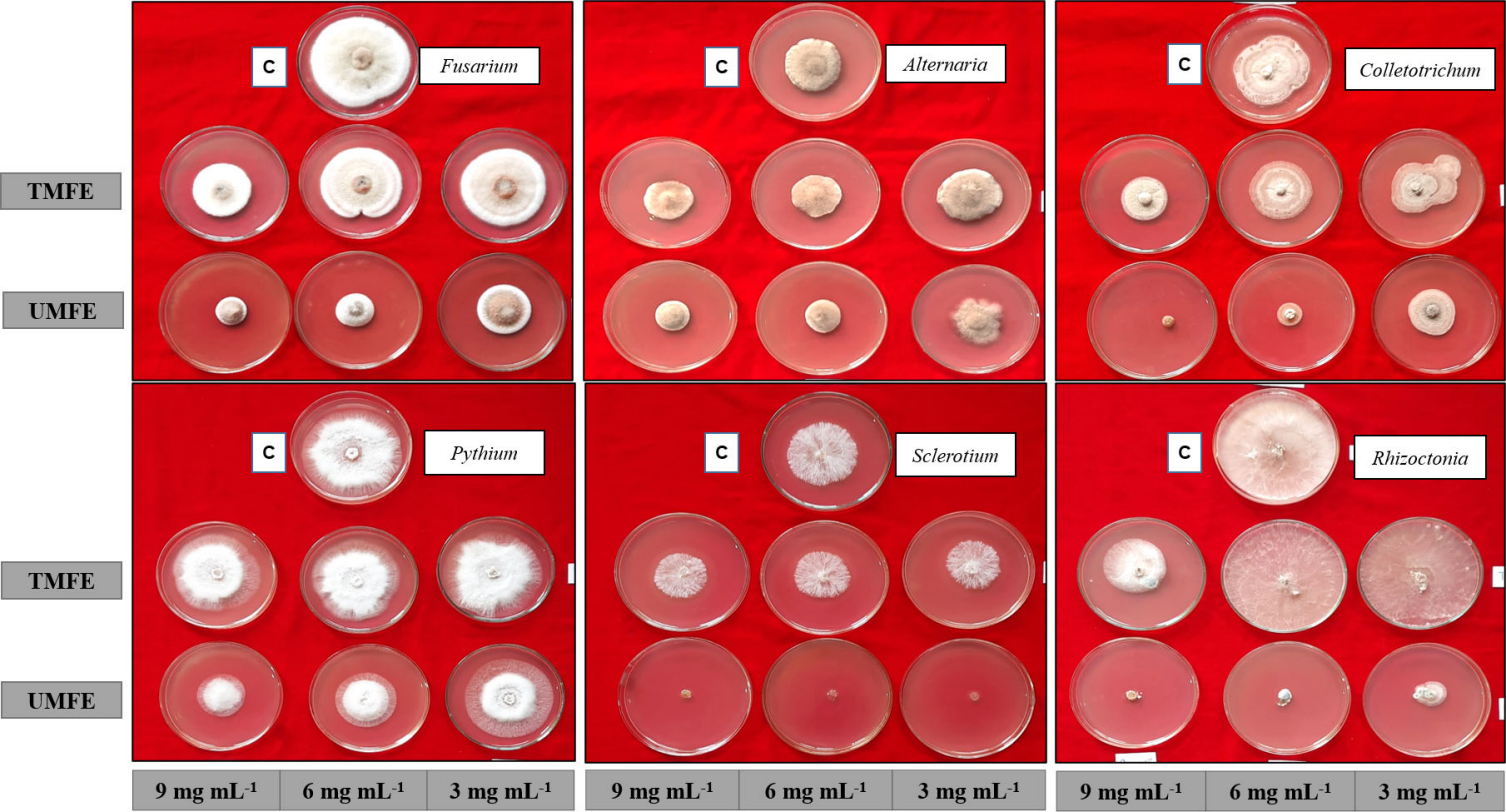
Antifungal activity using ethyl acetate fraction of TMFE and UMFE at different concentrations. TMFE, treated marigold flower effluent; UMFE, untreated marigold flower effluent; C, control.

Some groups of volatile and semi-volatile compounds are reported to possess antifungal activity, namely phenolics (Yang and Jiang, 2015), flavonoids (Orhan et al., 2010), alkene (Gakuubi et al., 2017), carboxylic acid (Varsha et al., 2015), and heterocyclic compounds (Sztanke et al., 2008), and a similar mixture of compounds found in the ethyl acetate fraction of TMFE and UMFE were found to have antifungal activity against plant pathogens (**Figure 4**).

As mentioned above, due to the presence of some antimicrobial compounds in the effluent, an antifungal *in vitro* bioassay was conducted, and the results are as follows. Upon treatment with TMFE at 3 mg mL^−1^, the highest percent inhibition was observed in *Sclerotium rolfsii* (23.39%), followed by *Fusarium oxysporum* (20.42%) and *Colletotrichum gleosporides* (16.11%). Significantly, the lowest inhibition of mycelial growth was observed in *Pythium aphanidermata* (6.99%). At 6 mg mL^−1^, the pathogens *S. rolfsii* and *C. gleosporides* reported the highest percent inhibition of 26.89% and 29.44%, respectively, and were on a par with each other. The next best pathogens that were inhibited were *Alternaria alternata* (24.54%) and *F. oxysporum* (22.64%). The lowest inhibition with 6 mg mL^−1^ was observed in *Rhizoctonia solani* (10.65%) and *P. aphanidermata* (10.33%). With an increased concentration of 9 mg mL^−1^, there was more than a 30% inhibition of all six pathogens, except for *P. aphanidermata* (15.63%). The growth of *F. oxysporum* and *C. gleosporides* were inhibited at 49.58% and 51.11%, respectively (**Table 3**). The data were statistically evaluated by the fractional factorial concept.

**Table 3:** Effect of MFE at different concentrations on inhibition of fungal plant pathogens.

Treated Marigold Flower Effluent (TMFE) – Per cent inhibition (%)				
Interaction (A × B)	B_1_: 3 mg mL^−1^	B_2_: 6 mg mL^−1^	B_3_: 9 mg mL^−1^	Mean inhibition of pathogen (A)
**A_1_:** *Fusarium oxysporum*	20.42 ± 0.72	22.64 ± 0.16	49.58 ± 1.44	30.88
**A_2_:** *Alternaria alternata*	14.39 ± 1.31	24.54 ± 0.60	31.82 ± 2.07	23.58
**A_3_:** *Colletotrichum gleosporides*	16.11 ± 0.96	29.44 ± 1.47	51.11 ± 1.55	32.22
**A_4_:** *Pythium aphanidermata*	6.99 ± 0.71	10.33 ± 0.40	15.63 ± 1.39	10.99
**A_5_:** *Sclerotium rolfsii*	23.39 ± 0.43	26.89 ± 0.91	38.51 ± 1.44	29.60
**A_6_:** *Rhizoctonia solani*	10.27 ± 0.24	10.65 ± 0.68	44.57 ± 1.34	21.83
**Mean inhibition at concentration (B)**	15.26	20.75	38.54	
Untreated Marigold Flower Effluent (UMFE) – Per cent inhibition (%)				
**Interaction (A × B)**	**B_1_: 3 mg mL^−1^**	**B_2_: 6 mg mL^−1^**	**B_3_: 9 mg mL^−1^**	**Mean inhibition of pathogen (A)**
**A_1_:** *Fusarium oxysporum*	53.50 ± 0.33	65.34 ± 1.37	74.87 ± 0.38	64.57
**A_2_:** *Alternaria alternata*	15.08 ± 0.73	47.35 ± 0.47	49.09 ± 0.45	37.17
**A_3_:** *Colletotrichum gleosporides*	42.94 ± 0.42	72.00 ± 0.67	91.22 ± 0.19	68.72
**A_4_:** *Pythium aphanidermata*	17.16 ± 0.33	44.07 ± 0.33	56.54 ± 0.25	39.26
**A_5_:** *Sclerotium rolfsii*	91.78 ± 0.10	92.76 ± 0.34	92.82 ± 0.10	92.45
**A_6_:** *Rhizoctonia solani*	79.81 ± 0.41	90.19 ± 0.24	98.57 ± 0.29	89.52
**Mean inhibition at concentration (B)**	50.04	68.62	77.19	
	TMFE		UMFE	
	SE(m)	CD @ 5%	SE(m)	CD @ 5%
**Factor (A)**	1.71	4.92	0.16	0.48
**Factor (B)**	1.21	3.48	0.12	0.34
**Interaction (A X B)**	2.96	8.53	0.28	0.83

± SD of Means; SE(m), Standard Error Mean; CD @ 5%, Critical Difference at 5% level of significance.

A fractional factorial design provides information on mean percent inhibitions, and on average, the mean inhibition of any selected pathogen by the use of TMFE at 3 mg mL^−1^ was 15.26%; at 6 mg mL^−1^, it was 20.75%, and at 9 mg mL^−1^, it was 38.54%. Irrespective of the concentration, TMFE was found to inhibit different pathogens with a mean inhibition percent in the decreasing order of *F. oxysporum* (30.88%), *A. alternata* (23.59%), *C. gleosporides* (32.22%), *P. aphanidermata* (10.99%), *S. rolfsii* (29.60%), and *R. solani* (21.83%) (**Table 3**). Similar results with a concentration increase were obtained with UMFE against plant pathogens. A percent inhibition of 91.78 was observed in *S. rolfsii* at a concentration of 3 mg mL^−1^ followed by *R. solani* (79.81%). Significantly, the lowest inhibition percentages of 15.08 and 17.16 were observed in pathogens *A. alternata* and *P. aphanidermata*, respectively. With a concentration of 6 mg mL^−1^, more than 40% of inhibition was reported in all the treatments, with the highest percentages of 92.76% (*S. rolfsii*) and 90.19% (*R. solani*) and lowest percentages of 47.35% (*A. alternata*) and 44.07% (*P. aphanidermata*). With an increased concentration of 9 mg mL^−1^ in the media, more than 90% of the inhibition of pathogens such as *R. solani* (98.56%), *S. rolfsii* (92.82%), and *C. gleosporides* (91.22%) was observed. The lowest inhibition percent among the selected pathogens was reported in *A. alternata* (49.09%), *P. aphanidermata* (56.54%), and *F. oxysporum* (74.87%) (**Table 3**).

The interaction effect of the different concentrations of MFE on different plant pathogens was evaluated and presented in **Table 3**. The individual studies using UMFE provide the significant mean inhibition percentage of any pathogen, and it was observed to be 50.04% at 3 mg mL^−1^, 68.62% at 6 mg mL^−1^, and 77.19% at 9 mg mL^−1^. The factorial studies provide evidence that, at almost all the concentrations, UMFE was able to inhibit the growth of all the selected plant pathogens, indicating greater antifungal activity. Regardless of the concentration, UMFE could induce the inhibition of growth in the following fashion: A_5_ > A_6_ > A_3_ > A_1_ > A_4_ > A_2_.

The results infer that there was a decrease in the mycelial diameter in all six pathogens with an increase in the concentration of TMFE and UMFE. There are many reports in the literature of concentration-dependent antifungal activity, whereby the colony diameters increase with a decrease in the concentration (poisoned food bioassay) (Rana et al., 2011; Aguiar et al., 2014). A study was conducted using methanol extract and a 10% decoction of the marigold flowers to assess their activity against anaerobic and facultative aerobic periodontal bacteria, and the results showed inhibition against the selected bacterial with MIC > 2048 mg L^−1^ (Iauk et al., 2003).

In the present study, the observed inhibition of fungal pathogens may be due to the presence of alkenes, esters, and carboxylic acid class compounds in both TMFE and UMFE, as reported in the GC-MS data. Alkene compounds (Cetene, 1-Nonadecene, 1-Tetracosene, 1-Octadecene, and 1-Hexacosene) were observed in the GC-MS profile of TMFE, which might have hindered the mycelial growth of the fungal pathogens. The inhibition due to the bioactive compounds is confirmed by previous findings indicating that alkene compounds were able to inhibit mycelial growth in five *Fusarium* sp. at a concentration of 7–8 µL mL^−1^ (Gakuubi et al., 2017). Certain reports published the effect of flavonoids as an antifungal effect (Orhan et al., 2010; Jin, 2019), and observed a flavonoid, a polyphenol compound (7, 9-Di-tert-butyl-1-oxaspiro (4, 5) deca-6, 9-diene-2, 8-dione) (Rosa et al., 2022), in TMFE. The proposed mechanism of antifungal activity by use of MFE may be due to the inhibition of spore germination by preventing the emergence of a normal germ tube, leading to abnormal swelling and the branching of hyphae (Paul et al., 2022).

Other important phytoconstituents preventing fungal growth are phenolic compounds (Catechol, Resorcinol and phenol, 2, 6-dimethoxy) and carboxylic acid compounds (Cyclohexane carboxylic acid, Octanoic acid, Hydrocinnamic acid, trans-4-Hydroxycyclohexane carboxylic acid, 2-Ethyl-1, 3-dimethyl cyclopent-2-ene carboxylic acid, Benzenepropanoic acid, Tetradecanoic acid, Benzoic acid, n-Hexadecanoic acid, Oleic Acid, and Octadecanoic acid) in UMFE at higher concentrations. Few researchers have reported the association of phenolics (De Rijke et al., 2006) and carboxylic acid (Varsha et al., 2015; Ismun et al., 2018) with antifungal activity. The results obtained are confirmed based on the previous studies on the prevention of the spore germination of *F. oxysporum* with 100 μg mL^−1^ of 2, 4-di-tert-butyl phenol (Dharni et al., 2014). It has been demonstrated that phenols inhibit the assembly of spindle microtubules and disturb the chromosomal alignment at the metaphase plate and microtubule–kinetochore interactions, causing chromatid loss, which may reduce the mycelia growth and germination of spores (Nam et al., 2004; Morais-Braga et al., 2017). The heterocyclic compound (2-Furanmethanol, tetrahydro) may also have promoted antifungal activity in this study, as triazoles, a heterocyclic compound, was reported to have antifungal activity against *Aspergillus niger* and *F. oxysporum* (Sztanke et al., 2008; Shalini et al., 2011).

In a few previous studies, essential oils reported better performance in their antifungal activity, and they contained major compounds such as b-caryophyllene (16.98%), d-cadinene (12.22%), a-cubebene (11.33%), 1,2-benzenedi carboxylic acid (10.17%) and caryophyllene oxide (7.74%) (Iauk et al., 2003; Rana et al., 2011; Aguiar et al., 2014), b-himachalene (4.68%), T-cadinol (3.98%), tetratetracontanal (3.83%), 1H-cyclopropa[a]naphthalene (3.56%), and b-farnesene (3.08%), which contributed to the antifungal activity (Daoubi et al., 2005; Guo et al., 2008). In a study, the oil from the flowers of the Asteraceae family was found to exhibit antifungal activity against the fungal strains of *Candida* spp (Gazim et al., 2008). In addition, the inhibition of the *F. oxysporum* conidium, a soilborne pathogen, by *T. minuta* was reported (Sadia et al., 2013).

The above-discussed studies mainly refer to the organic compounds belonging to the class of alkenes, aldehyde, phenol, and carboxylic acid. These studies coincide with the results obtained in this study, indicating that using MFE as an agro-industry waste possessing numerous beneficial volatile and semi-volatile compounds has a potential role in promoting antifungal activity. Moreover, GC-MS chromatographic data provide stronger evidence to the results obtained in the *in vitro* plate bioassay.

## 4 Conclusions

This study attempted to analyze the properties of agro-industrial wastewater, with the primary goal of conceptualizing the organic compound existing in effluent sources and its significance in promoting antifungal activity against plant pathogens. From the GC-MS findings, the potential for several organic compounds to promote antifungal activity is discussed. Unfortunately, due to the large number of organic compounds found in the effluent, it is practically impossible to separate specific organic compounds. Furthermore, because all the phytoconstituents discovered by GC-MS are organic, sorting them quantitatively and qualitatively is a complex job. Henceforth, a statistical approach, namely 2D-PCA, was applied for a clear understanding of the constituents based on their functional groups.

The present investigation confirms that the marigold flower effluent, an industrial waste, can be used as a plant antifungal agent. The study on marigold flower effluent revealed that it comprises significant phytoconstituents that inhibit common plant pathogens by more than 50%. By conducting preliminary research of its composition, the study expands the possibilities for agro-industry effluents, mainly in the marigold pro-cessing industry, to be used in agriculture as an eco-friendly antifungal agent possessing antimicrobial properties. The study can be deepened by conducting a pot culture experiment wherein plants that are inoculated with a validated pathogen are then treated with different concentrations of effluent. This research not only benefits the industry by reducing the cost of effluent treatment and disposal, but also benefits farming communities by minimizing the use of synthetic fungicides, conserving the health of the soil, and reducing the environmental impact by using the waste marigold industrial effluent as a fungicide.

## Data availability statement

The raw data supporting the conclusions of this article will be made available by the authors, without undue reservation.

## Author contributions

TS: Conceptualization, Formal Analysis, Methodology, Validation, Writing – original draft, Visualization. UN: Supervision, Validation, Visualization, Writing – review & editing. BP: Conceptualization, Investigation, Supervision, Validation, Resources, Writing – review & editing. SN: Software, Supervision, Validation, Writing – review & editing. JR: Conceptualization, Investigation, Resources, Supervision, Validation, Writing – review & editing. KG: Conceptualization, Resources, Software, Supervision, Validation, Writing – review & editing. SV: Methodology, Software, Writing – review & editing.
