# Supplementary material for: Evaluation of phytoconstituents in marigold effluent for their antifungal activity against plant pathogens
Source: Front Fungal Biol. 2024 Apr 4;5:1345543. doi: 10.3389/ffunb.2024.1345543 (PMC11025535; doi:10.3389/ffunb.2024.1345543)

Evaluation of Phytoconstituents in Marigold (*Tagetes erecta* L.) Effluent for Their Antifungal Activity against Plant Pathogens

Tulja Sanam [1,] *, Umashankar Nagaraju [1], Benherlal P. S [2], Sridhar Goud Nerella [3], Jayaramaiah. R [4], Kadalli. G. G [5] and Satya Srii. V [6]

[1]Department of Agricultural Microbiology, University of Agricultural Sciences, Bangalore 560065, India

[2]Department of Plant Biotechnology, University of Agricultural Sciences, Bangalore 560065, India; benherlal@gmail.com

[3]Department of Medicinal Chemistry, National Institute of Pharmaceutical Education and Research (NIPER), Hyderabad 500037, India; sridhar.ku23@gmail.com

[4]Department of Agronomy, University of Agricultural Sciences, Bangalore 560065, India; jayaram.uas@gmail.com

[5]Department of Soil Science and Analytical Chemistry, University of Agricultural Sciences, Bangalore 560065, India; ggkadalli@rediffmail.com

[6]Department of Seed Science and Technology, University of Agricultural Sciences, Bangalore 560065, India; sriinila@gmail.com

*Correspondence: tuljasanam@gmail.com (T.S.)

Sl. No.	Title	Page No.
1.	Figure S1: Processing of Marigold flowers	3
2.	Figure S2: Treatment process of untreated effluent after processing flowers	4
3.	Principal Component Analysis (PCA) Data- PC Scores, Scoreplot, Eigenvalues, Covariance matrix and standardized values of both TMFE and UMFE components (a-f)	5, 6
4.	Identification of compounds based on fragments data using NIST library	7

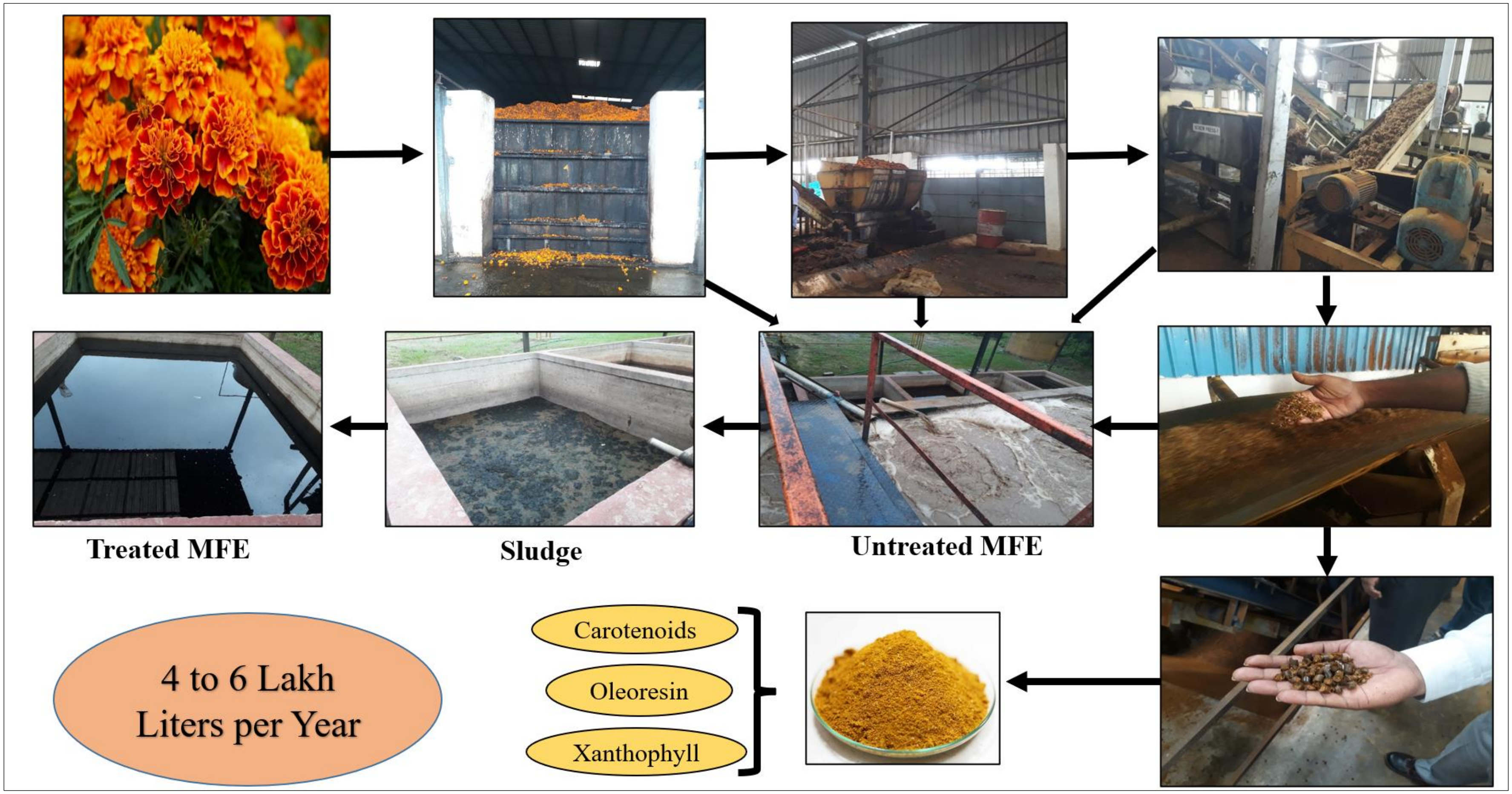

Fig. S1: Processing of marigold flowers in the industry and collection of treated and untreated marigold flower effluent (TMFE and UMFE) during the process –
Flow chart: The process is as follows collecting marigold flowers, silage preparation, pressing, drying, pelletization and extraction of carotenoids, oleoresins and xanthophylls.

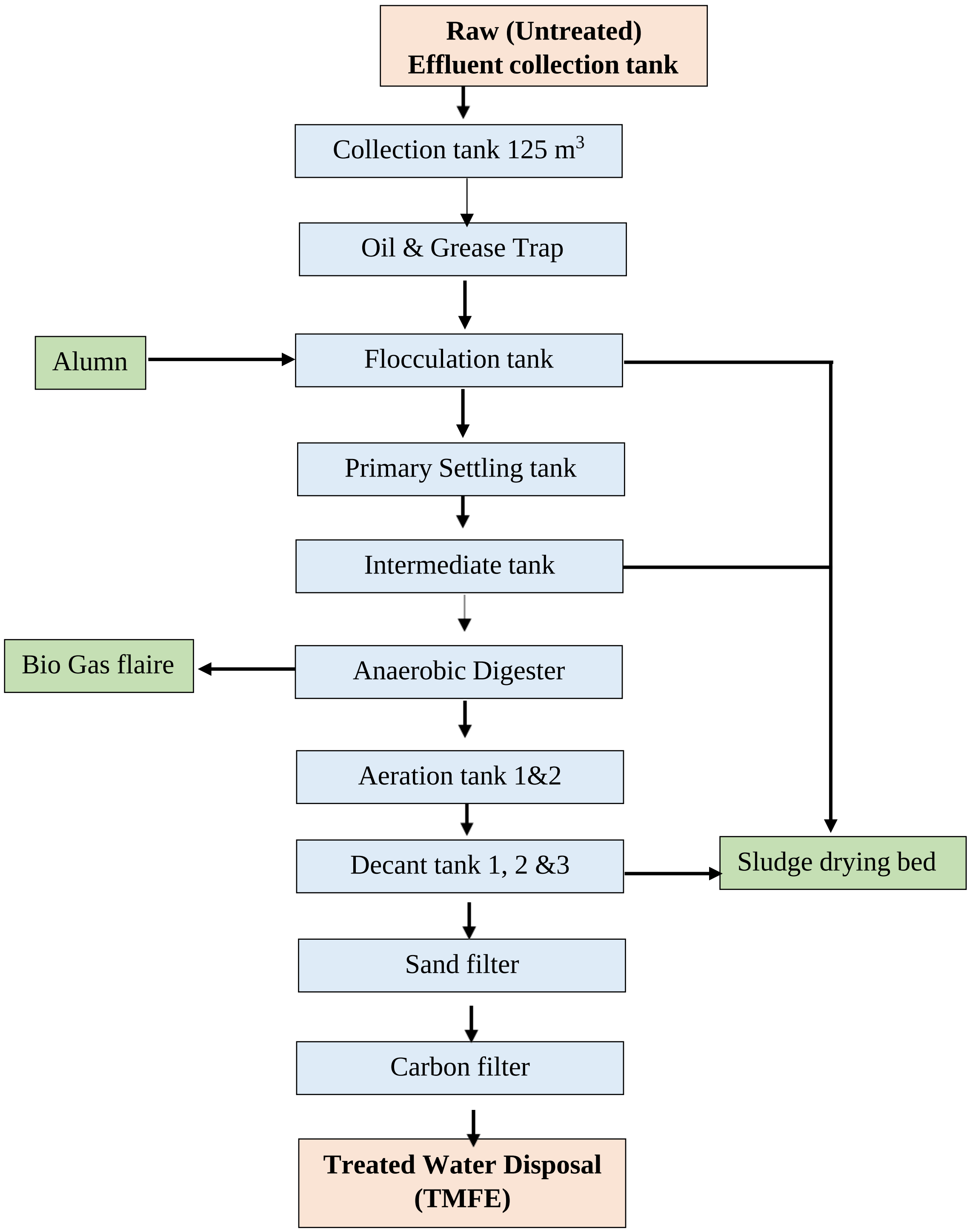

Fig. S2: Flow chart for treatment of untreated marigold flower effluent (UMFE) to formTreated marigold flower effluent (TMFE)

- The collected untreated effluent at different stages is collected into a collection tankGrease and oil is removed using different sized sieve traps
- Further moved to Flocculation tank where alum is added for sedimentation
- Later transferred to Primary settling tank, Aeration tank, Decant tank, During this process Biogas is collected for other purposesand the Effluent free of solid particles, oil and grease are passed through Sand and Carbon Filters for safe Disposal

Principle Component Analysis (PCA) Raw Data

a) **Functional group compounds in TMFE and UMFE**

Organic Compounds group	TMFE	UMFE
Carboxylic acid	4	11
Alkene	5	2
Ester	3	1
Alcohol	2	2
Alkane	1	0
Aldehyde	1	0
Flavonoid	1	0
Cyanide	1	0
Polycyclic aromatic	0	1
Cyclo alkane	0	1
Cyclo alkene	0	1
Phenol	0	3
Heterocyclic	0	1

b) **Eigenvalues**

Parameter	PC_1	PC_2
Eigenvalue	1.5012	0.4988
% of Variance	75.0582	24.9418
Cumulative (%)	75.0582	100

c) **Scores**

Compounds	PC_1	PC_2
Carboxylic acid	3.3493	-1.1215
Alkene	1.5957	1.4839
Ester	0.5017	0.8743
Alcohol	0.318	0.2062
Alkane	-0.5923	0.2646
Aldehyde	-0.5923	0.2646
Flavonoid	-0.5923	0.2646
Cyanide	-0.5923	0.2646
Polycyclic aromatic	-0.776	-0.4034
Cyclo alkane	-0.776	-0.4034
Cyclo alkene	-0.776	-0.4034
Phenol	-0.2917	-0.8878
Heterocyclic	-0.776	-0.4034

d) **The covariance matrix**

Group\Group	TMFE	UMFE
TMFE	1	0.5012
UMFE	0.5012	1

e) The standardized table

Compounds	TMFE	UMFE
Carboxylic acid	1.5753	3.1614
Alkene	2.1776	0.07903
Ester	0.973	-0.2634
Alcohol	0.3707	0.07903
Alkane	-0.2317	-0.6059
Aldehyde	-0.2317	-0.6059
Flavonoid	-0.2317	-0.6059
Cyanide	-0.2317	-0.6059
Polycyclic aromatic	-0.834	-0.2634
Cyclo alkane	-0.834	-0.2634
Cyclo alkene	-0.834	-0.2634
Phenol	-0.834	0.4215
Heterocyclic	-0.834	-0.2634

f) Scoreplot

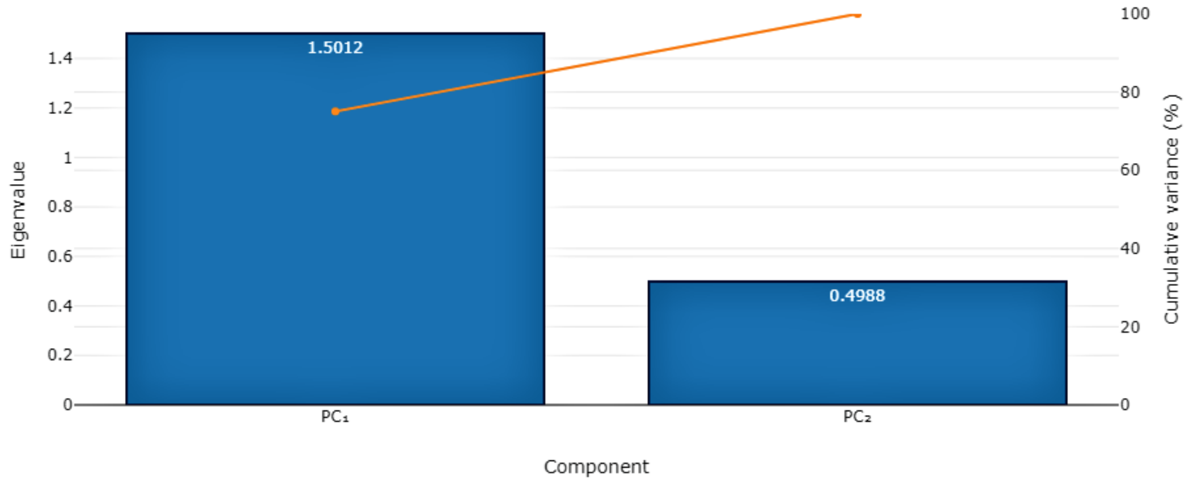

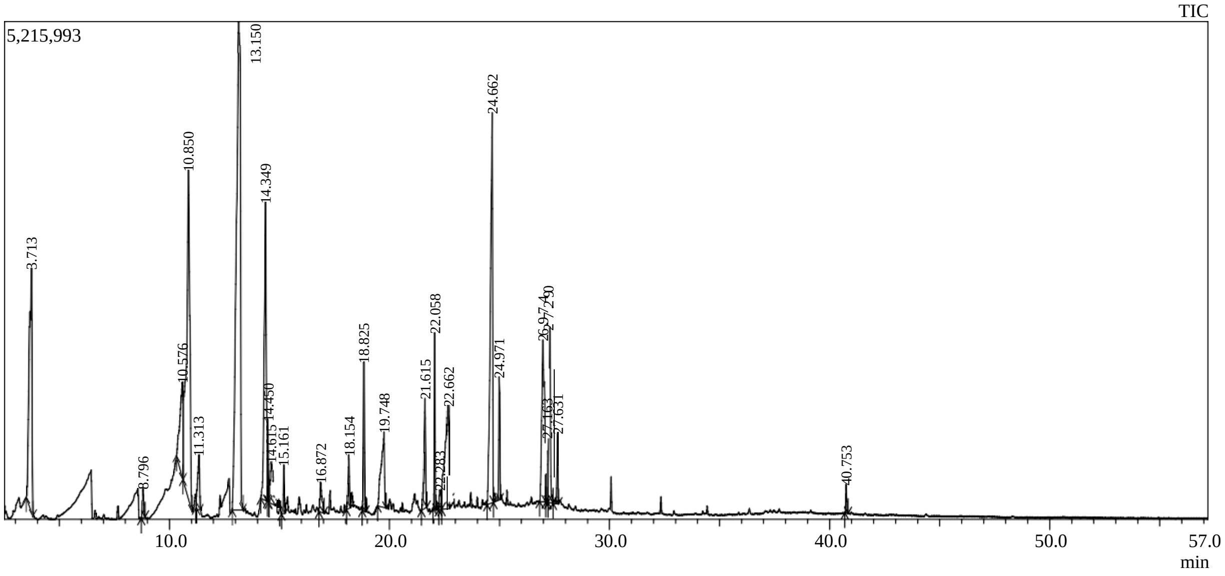

Peak Report TIC

Peak#	R.Time	I.Time	F.Time	Area	Area%	Height	Height%	A/H	Mark	Name
1	3.713	3.500	3.800	20136833	7.55	2544150	7.07	7.91		2-Furanmethanol, tetrahydro-
2	8.796	8.717	8.900	1333784	0.50	306887	0.85	4.35		Phenylethyl Alcohol
3	10.576	10.317	10.617	9580474	3.59	984436	2.74	9.73		Cyclohexanecarboxylic acid
4	10.850	10.617	11.033	34759682	13.02	3430057	9.53	10.13	V	Catechol
5	11.313	11.200	11.367	3236274	1.21	566791	1.57	5.71	V	Octanoic acid
6	13.150	12.833	13.333	65106687	24.40	5117352	14.22	12.72		Resorcinol
7	14.349	14.150	14.433	20242856	7.59	3122647	8.68	6.48		Phenol, 2,6-dimethoxy-
8	14.450	14.433	14.517	2338227	0.88	857900	2.38	2.73	V	Hydrocinnamic acid
9	14.615	14.517	14.817	4069238	1.52	434269	1.21	9.37	V	trans-4-Hydroxycyclohexanecar
10	15.161	15.100	15.250	1798824	0.67	506725	1.41	3.55		Carbonic acid, ethyl tetradecyl e
11	16.872	16.800	17.033	1821960	0.68	310183	0.86	5.87		2-Ethyl-1,3-dimethylcyclopent-2
12	18.154	18.050	18.233	2595443	0.97	559768	1.56	4.64		2-Cyclohexen-1-one, 3-(hydroxy
13	18.825	18.767	18.917	4692184	1.76	1559786	4.33	3.01		1-Pentadecene
14	19.748	19.467	19.850	8150945	3.05	787788	2.19	10.35		Benzenepropanoic acid, 4-hydro
15	21.615	21.450	21.700	5895152	2.21	1149838	3.19	5.13		Tetradecanoic acid
16	22.058	21.983	22.117	5400650	2.02	1849183	5.14	2.92		1-Nonadecene
17	22.283	22.233	22.367	1311174	0.49	204343	0.57	6.42	V	7,7-Dimethoxy-2,3,4,5,6,7-hexa
18	22.662	22.367	22.750	12710493	4.76	1074594	2.99	11.83	V	Benzoic acid, 4-hydroxy-3,5-dim
19	24.662	24.433	24.733	28680690	10.75	4088328	11.36	7.02		n-Hexadecanoic acid
20	24.971	24.733	25.017	3932359	1.47	1288963	3.58	3.05	V	1-Nonadecene
21	26.974	26.817	27.100	14167420	5.31	1693162	4.70	8.37		Oleic Acid
22	27.163	27.100	27.200	2973354	1.11	680279	1.89	4.37	V	7-Acetyl-3,6-dihydroxy-8-methy
23	27.290	27.200	27.433	8816407	3.30	1835572	5.10	4.80	V	Octadecanoic acid
24	27.631	27.433	27.683	2135418	0.80	742336	2.06	2.88	V	1-Nonadecene
25	40.753	40.683	40.833	986787	0.37	298114	0.83	3.31		Tris(2,4-di-tert-butylphenyl) pho
				266873315	100.00	35993451	100.00			

Library

<< Target >>
Line#:1 R.Time:3.717(Scan#:74) MassPeaks:358
RawMode:Averaged 3.700-3.733(73-75) BasePeak:71.10(460218)
BG Mode:Calc. from Peak Group 1 - Event 1 Scan

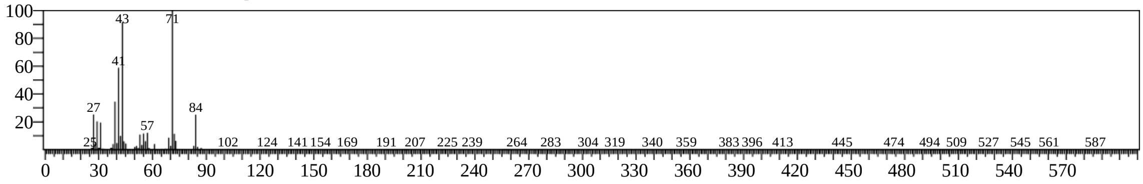

Hit#:1 Entry:2429 Library:NIST20R.lib
SI:89 Formula:C5H10O2 CAS:97-99-4 MolWeight:102 RetIndex:892
CompName:2-Furanmethanol, tetrahydro- $$ Furfuryl alcohol, tetrahydro- $$ QO THFA $$ Tetrahydro-2-furancarbinol $$ Tetrahydro-2-furanmethanol $$ T

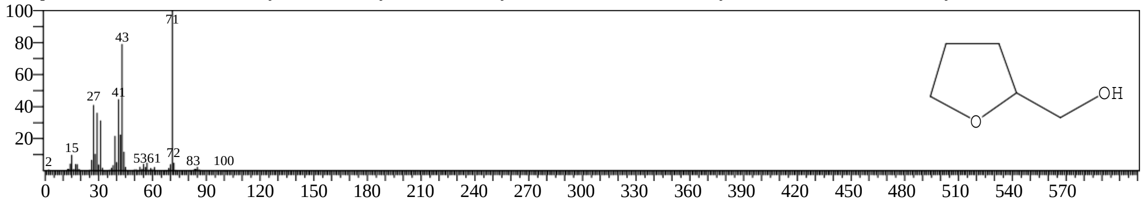

Hit#:2 Entry:1076 Library:NIST20R.lib
SI:88 Formula:C5H10O CAS:96-47-9 MolWeight:86 RetIndex:650
CompName:Furan, tetrahydro-2-methyl- $$ Tetrahydro-2-methylfuran $$ Tetrahydrosylvan $$ 2-Methyltetrahydrofuran $$ Furan, 2-methyl-tetrahydro- $$ M

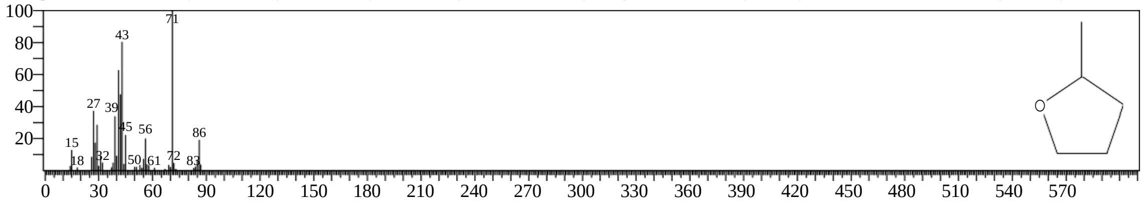

Hit#:3 Entry:2430 Library:NIST20R.lib
SI:88 Formula:C5H10O2 CAS:97-99-4 MolWeight:102 RetIndex:892
CompName:2-Furanmethanol, tetrahydro- $$ Furfuryl alcohol, tetrahydro- $$ QO THFA $$ Tetrahydro-2-furancarbinol $$ Tetrahydro-2-furanmethanol $$ T

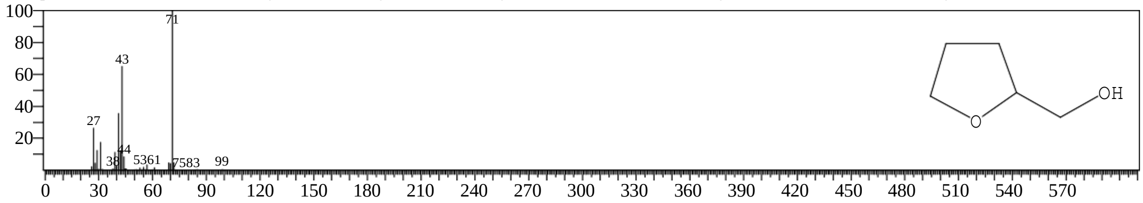

Hit#:4 Entry:2392 Library:NIST20M1.lib
SI:88 Formula:C5H10O2 CAS:15833-61-1 MolWeight:102 RetIndex:892
CompName:.+/-.-Tetrahydro-3-furanmethanol $$ Tetrahydro-3-furanylmethanol #

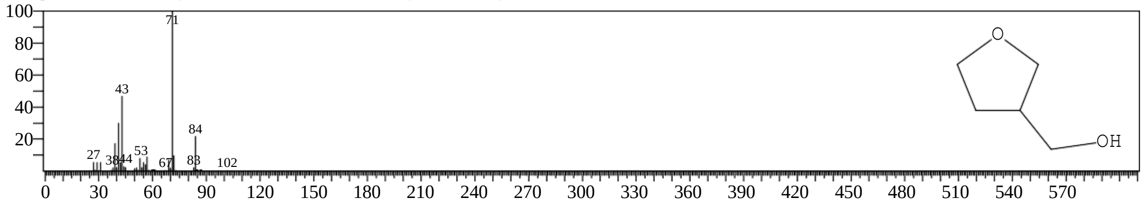

Hit#:5 Entry:2471 Library:NIST20M1.lib
SI:88 Formula:C6H14O CAS:19550-30-2 MolWeight:102 RetIndex:732
CompName:1-Butanol, 2,3-dimethyl- $$ 2,3-Dimethyl-1-butanol $$ 2,3-Methylbutanol $$ 2,3-Dimethylbutan-1-ol

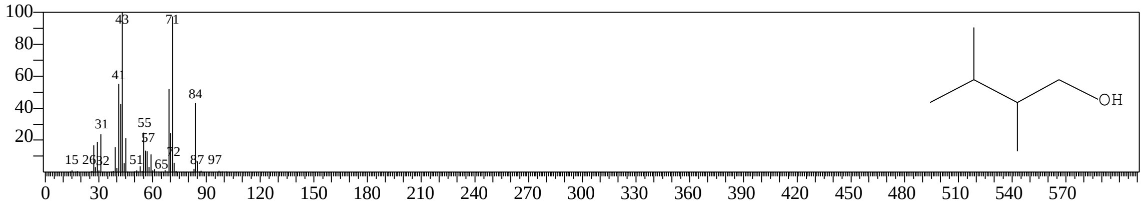

<< Target >>
Line#:2 R.Time:8.800(Scan#:379) MassPeaks:294
RawMode:Averaged 8.783-8.817(378-380) BasePeak:91.10(91471)
BG Mode:Calc. from Peak Group 1 - Event 1 Scan

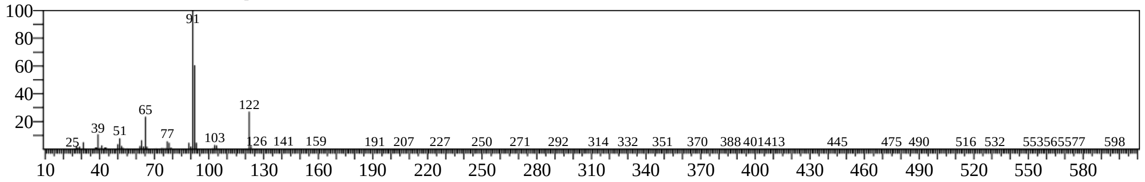

Hit#:1 Entry:6061 Library:NIST20M1.lib
SI:97 Formula:C8H10O CAS:60-12-8 MolWeight:122 RetIndex:1136
CompName:Phenylethyl Alcohol $$ Benzeneethanol $$ Phenethyl alcohol $$ .beta.-Hydroxyethylbenzene $$ .beta.-Phenethyl alcohol $$ .beta.-Phenylethan

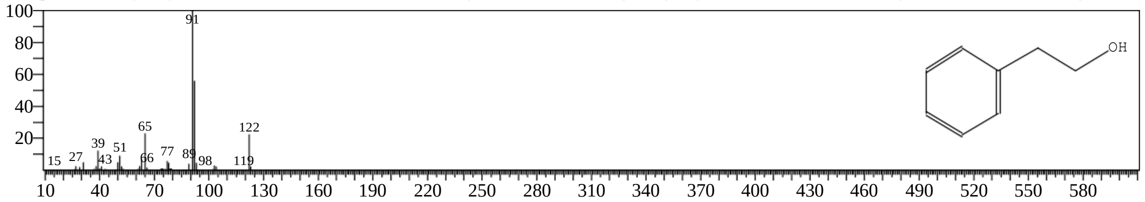

Hit#:2 Entry:5343 Library:NIST20R.lib
SI:96 Formula:C8H10O CAS:60-12-8 MolWeight:122 RetIndex:1136
CompName:Phenylethyl Alcohol $$ Benzeneethanol $$ Phenethyl alcohol $$ .beta.-Hydroxyethylbenzene $$ .beta.-Phenethyl alcohol $$ .beta.-Phenylethan

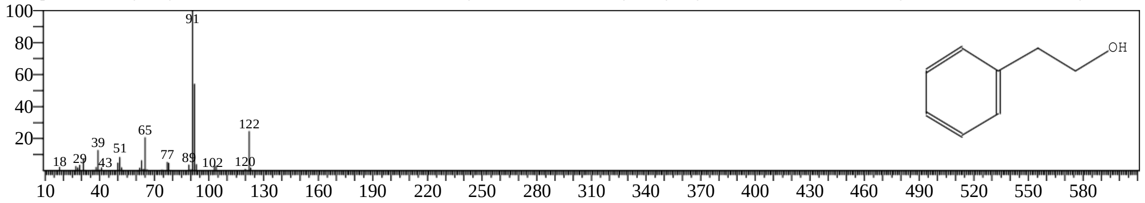

Hit#:3 Entry:5341 Library:NIST20R.lib
SI:96 Formula:C8H10O CAS:60-12-8 MolWeight:122 RetIndex:1136
CompName:Phenylethyl Alcohol $$ Benzeneethanol $$ Phenethyl alcohol $$ .beta.-Hydroxyethylbenzene $$ .beta.-Phenethyl alcohol $$ .beta.-Phenylethan

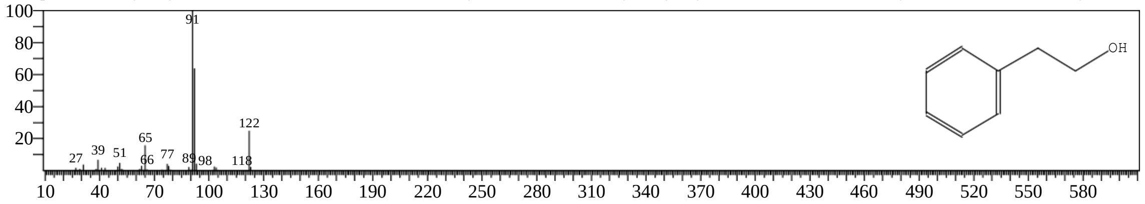

Hit#:4 Entry:5342 Library:NIST20R.lib
SI:96 Formula:C8H10O CAS:60-12-8 MolWeight:122 RetIndex:1136
CompName:Phenylethyl Alcohol $$ Benzeneethanol $$ Phenethyl alcohol $$ .beta.-Hydroxyethylbenzene $$ .beta.-Phenethyl alcohol $$ .beta.-Phenylethan

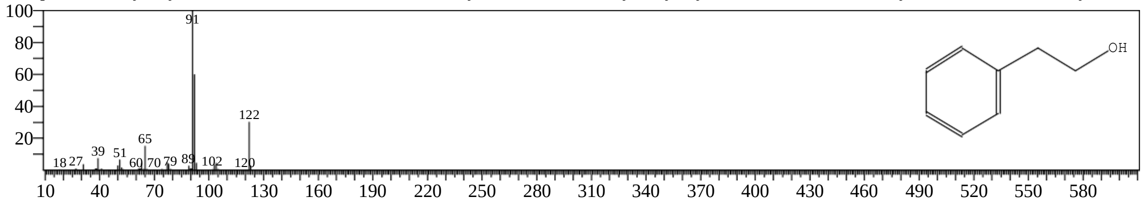

Hit#:5 Entry:5247 Library:NIST20R.lib
SI:89 Formula:C7H10N2 CAS:555-96-4 MolWeight:122 RetIndex:1235
CompName:Hydrazine, (phenylmethyl)- $$ Hydrazine, benzyl- $$ Benzylhydrazine $$ Benzyl hydrazide $$ 1-Benzylhydrazine # $$ (Phenylmethyl)hydrazi

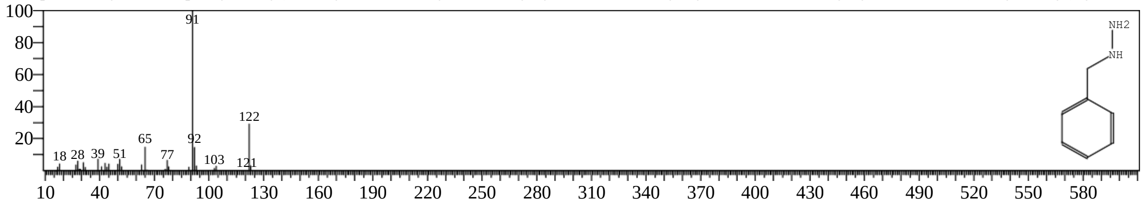

<< Target >>
Line#:3 R.Time:10.583(Scan#:486) MassPeaks:285
RawMode:Averaged 10.567-10.600(485-487) BasePeak:55.10(26496)
BG Mode:Calc. from Peak Group 1 - Event 1 Scan

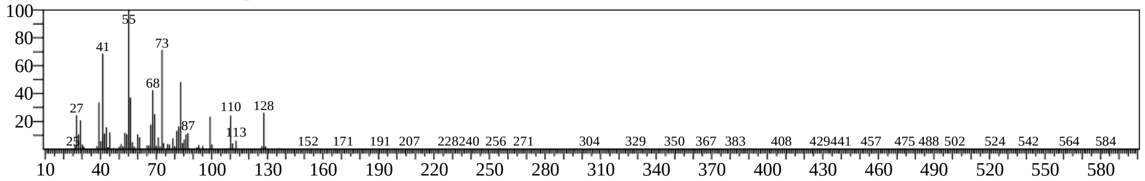

Hit#:1 Entry:7814 Library:NIST20M1.lib
SI:93 Formula:C7H12O2 CAS:98-89-5 MolWeight:128 RetIndex:1137
CompName:Cyclohexanecarboxylic acid $$ Benzoic acid, hexahydro- $$ Cyclohexylcarboxylic acid $$ Hexahydrobenzoic acid $$ Carboxycyclohexane $$

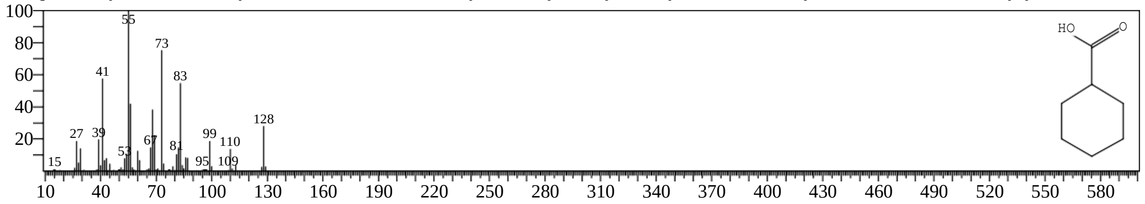

Hit#:2 Entry:6263 Library:NIST20R.lib
SI:93 Formula:C7H12O2 CAS:98-89-5 MolWeight:128 RetIndex:1137
CompName:Cyclohexanecarboxylic acid $$ Benzoic acid, hexahydro- $$ Cyclohexylcarboxylic acid $$ Hexahydrobenzoic acid $$ Carboxycyclohexane $$

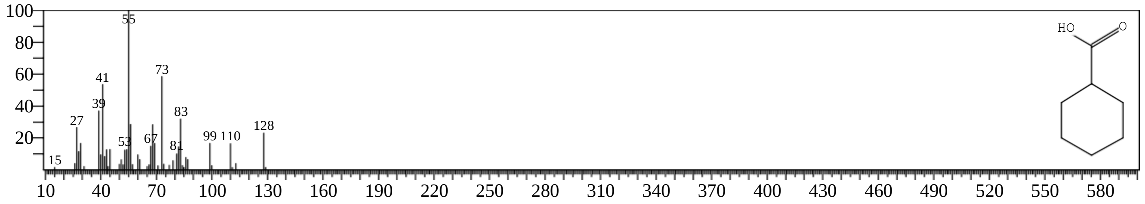

Hit#:3 Entry:6262 Library:NIST20R.lib
SI:92 Formula:C7H12O2 CAS:98-89-5 MolWeight:128 RetIndex:1137
CompName:Cyclohexanecarboxylic acid $$ Benzoic acid, hexahydro- $$ Cyclohexylcarboxylic acid $$ Hexahydrobenzoic acid $$ Carboxycyclohexane $$

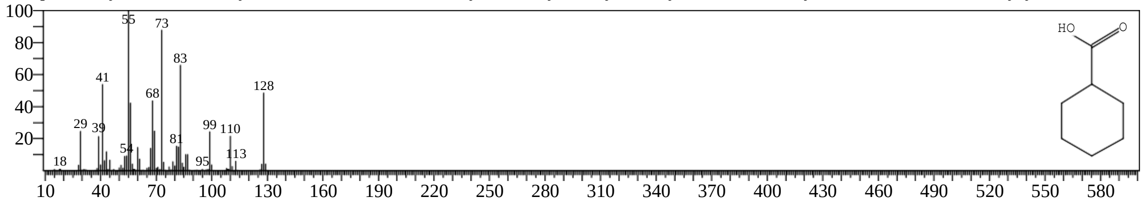

Hit#:4 Entry:7864 Library:NIST20M1.lib
SI:83 Formula:C7H12O2 CAS:18999-28-5 MolWeight:128 RetIndex:1081
CompName:2-Heptenoic acid $$ Ethyl 2-heptenoate $$ (2E)-2-Heptenoic acid #

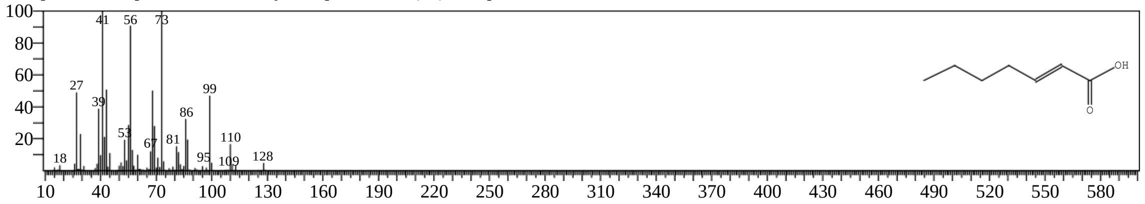

Hit#:5 Entry:7865 Library:NIST20M1.lib
SI:82 Formula:C7H12O2 CAS:10352-88-2 MolWeight:128 RetIndex:1081
CompName:Trans-2-heptenoic acid $$ 2-Heptenoic acid, (E)- $$ 2-Heptenoic acid, trans-

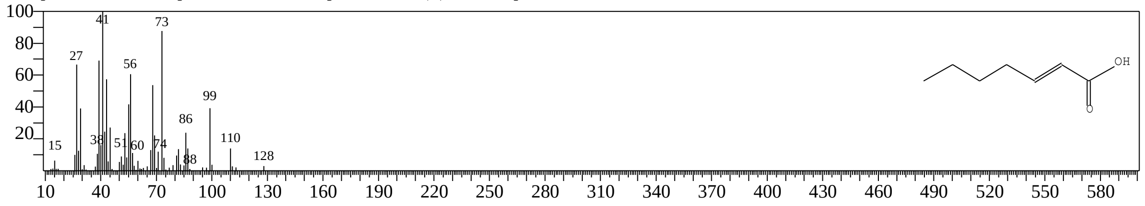

<< Target >>
Line#:4 R.Time:10.850(Scan#:502) MassPeaks:405
RawMode:Averaged 10.833-10.867(501-503) BasePeak:110.10(529944)
BG Mode:Calc. from Peak Group 1 - Event 1 Scan

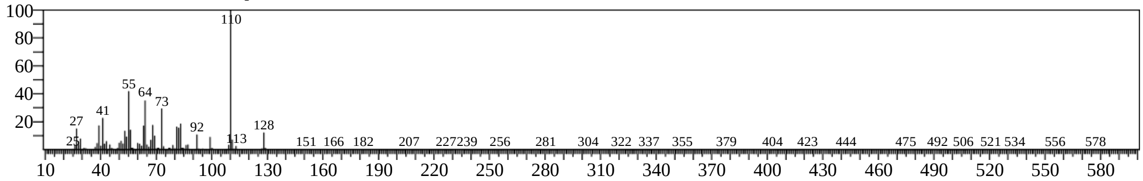

Hit#:1 Entry:3235 Library:NIST20R.lib
SI:81 Formula:C6H6O2 CAS:120-80-9 MolWeight:110 RetIndex:1122
CompName:Catechol $$ 1,2-Benzenediol $$ Pyrocatechol $$ o-Benzenediol $$ o-Dihydroxybenzene $$ o-Dioxybenzene $$ o-Hydroxyphenol $$ o-Phenyl

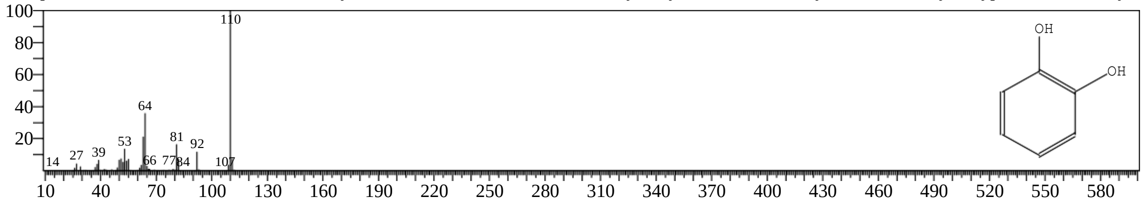

Hit#:2 Entry:3234 Library:NIST20R.lib
SI:80 Formula:C6H6O2 CAS:120-80-9 MolWeight:110 RetIndex:1122
CompName:Catechol $$ 1,2-Benzenediol $$ Pyrocatechol $$ o-Benzenediol $$ o-Dihydroxybenzene $$ o-Dioxybenzene $$ o-Hydroxyphenol $$ o-Phenyl

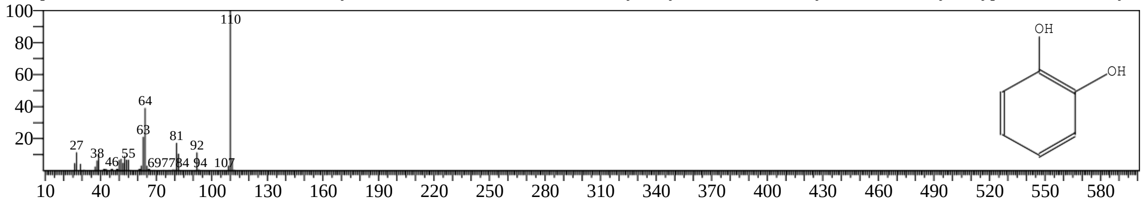

Hit#:3 Entry:3218 Library:NIST20R.lib
SI:79 Formula:C6H6O2 CAS:108-46-3 MolWeight:110 RetIndex:1122
CompName:Resorcinol $$ 1,3-Benzenediol $$ .alpha.-Resorcinol $$ m-Benzenediol $$ m-Dihydroxybenzene $$ m-Dioxybenzene $$ m-Hydroquinone $$

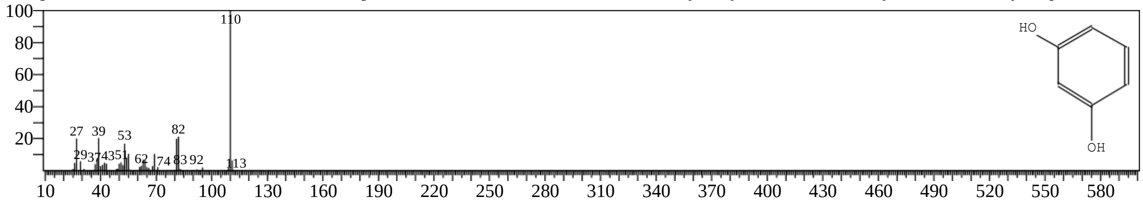

Hit#:4 Entry:3230 Library:NIST20R.lib
SI:79 Formula:C6H6O2 CAS:123-31-9 MolWeight:110 RetIndex:1122
CompName:Hydroquinone $$ 1,4-Benzenediol $$ p-Benzenediol $$ p-Dihydroxybenzene $$ p-Dioxybenzene $$ p-Hydroquinone $$ p-Hydroxyphenol $$

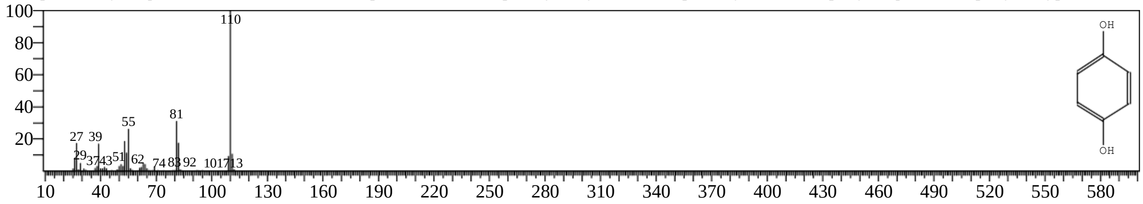

Hit#:5 Entry:3219 Library:NIST20R.lib
SI:78 Formula:C6H6O2 CAS:108-46-3 MolWeight:110 RetIndex:1122
CompName:Resorcinol $$ 1,3-Benzenediol $$ .alpha.-Resorcinol $$ m-Benzenediol $$ m-Dihydroxybenzene $$ m-Dioxybenzene $$ m-Hydroquinone $$

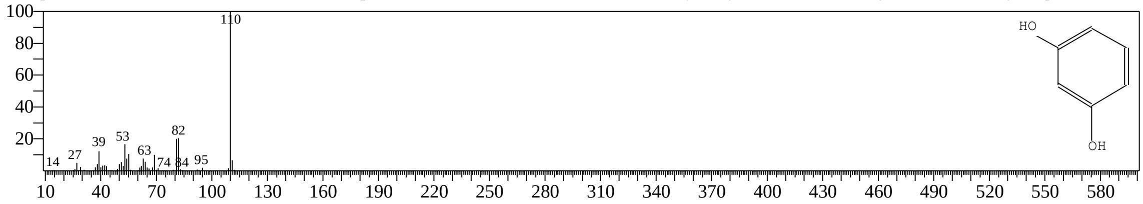

<< Target >>
Line#:5 R.Time:11.317(Scan#:530) MassPeaks:270
RawMode:Averaged 11.300-11.333(529-531) BasePeak:60.05(86420)
BG Mode:Calc. from Peak Group 1 - Event 1 Scan

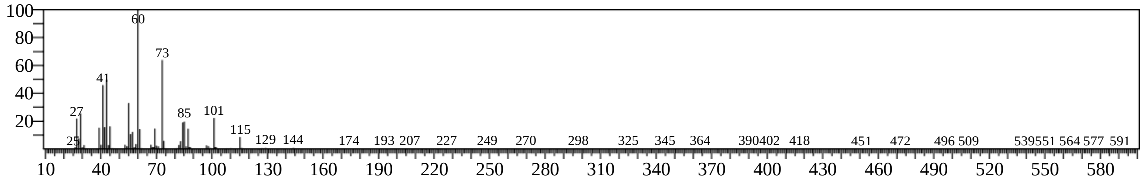

Hit#:1 Entry:14424 Library:NIST20M1.lib
SI:97 Formula:C8H16O2 CAS:124-07-2 MolWeight:144 RetIndex:1173
CompName:Octanoic acid $$ n-Caprylic acid $$ n-Octanoic acid $$ n-Octoic acid $$ n-Octylic acid $$ Neo-Fat 8 $$ Caprylic acid $$ Enantic acid $$ Octyl

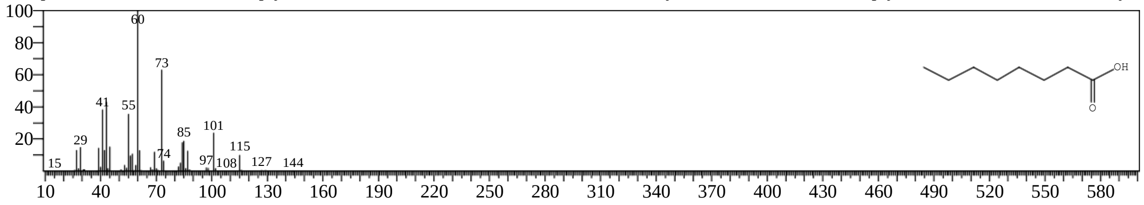

Hit#:2 Entry:9790 Library:NIST20R.lib
SI:95 Formula:C8H16O2 CAS:124-07-2 MolWeight:144 RetIndex:1173
CompName:Octanoic acid $$ n-Caprylic acid $$ n-Octanoic acid $$ n-Octoic acid $$ n-Octylic acid $$ Neo-Fat 8 $$ Caprylic acid $$ Enantic acid $$ Octyl

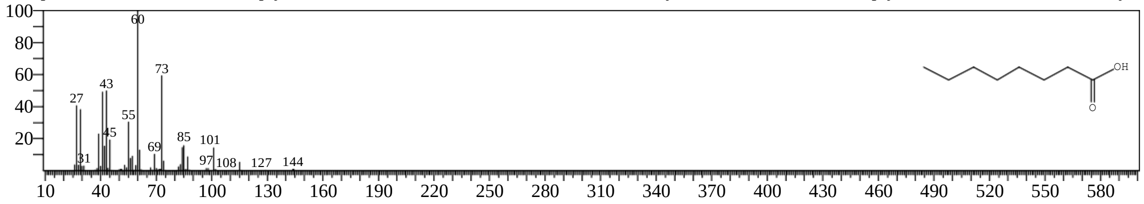

Hit#:3 Entry:9791 Library:NIST20R.lib
SI:94 Formula:C8H16O2 CAS:124-07-2 MolWeight:144 RetIndex:1173
CompName:Octanoic acid $$ n-Caprylic acid $$ n-Octanoic acid $$ n-Octoic acid $$ n-Octylic acid $$ Neo-Fat 8 $$ Caprylic acid $$ Enantic acid $$ Octyl

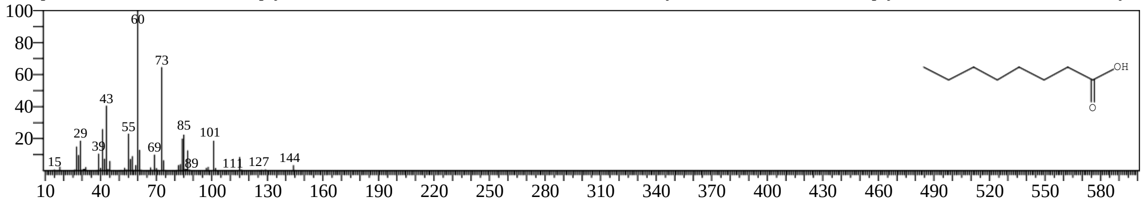

Hit#:4 Entry:9792 Library:NIST20R.lib
SI:93 Formula:C8H16O2 CAS:124-07-2 MolWeight:144 RetIndex:1173
CompName:Octanoic acid $$ n-Caprylic acid $$ n-Octanoic acid $$ n-Octoic acid $$ n-Octylic acid $$ Neo-Fat 8 $$ Caprylic acid $$ Enantic acid $$ Octyl

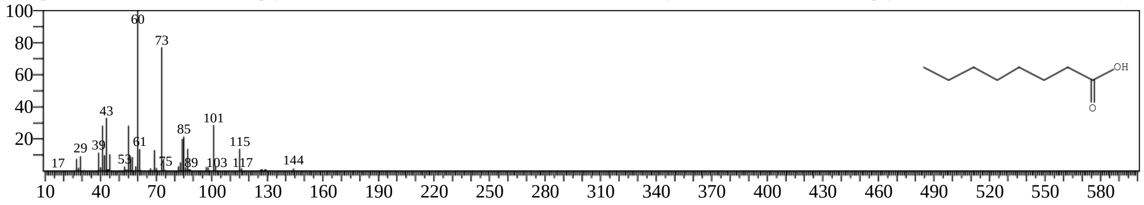

Hit#:5 Entry:13187 Library:NIST20R.lib
SI:90 Formula:C9H18O2 CAS:112-05-0 MolWeight:158 RetIndex:1272
CompName:Nonanoic acid $$ n-Nonanoic acid $$ n-Nonoic acid $$ n-Nonylic acid $$ Nonoic acid $$ Nonylic acid $$ Pelargic acid $$ Pelargonic acid $$

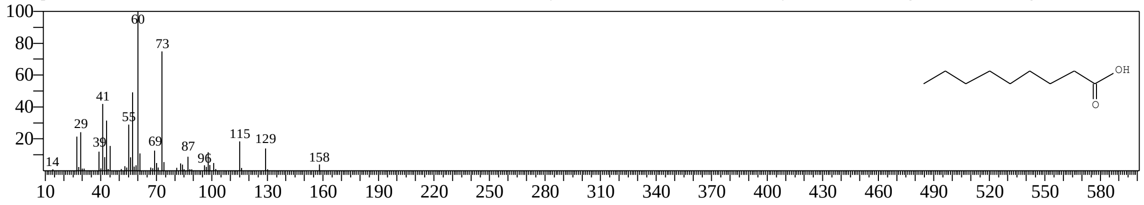

<< Target >>
Line#:6 R.Time:13.150(Scan#:640) MassPeaks:423
RawMode:Averaged 13.133-13.167(639-641) BasePeak:110.10(1845796)
BG Mode:Calc. from Peak Group 1 - Event 1 Scan

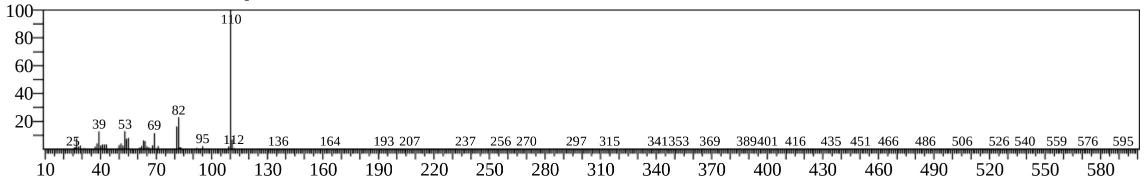

Hit#:1 Entry:3219 Library:NIST20R.lib
SI:97 Formula:C6H6O2 CAS:108-46-3 MolWeight:110 RetIndex:1122
CompName:Resorcinol $$ 1,3-Benzenediol $$ .alpha.-Resorcinol $$ m-Benzenediol $$ m-Dihydroxybenzene $$ m-Dioxybenzene $$ m-Hydroquinone $$

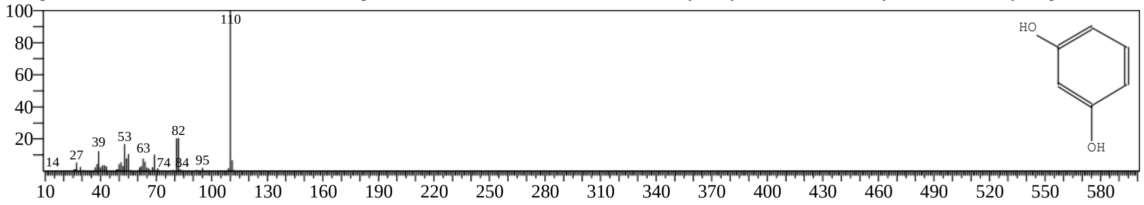

Hit#:2 Entry:3218 Library:NIST20R.lib
SI:94 Formula:C6H6O2 CAS:108-46-3 MolWeight:110 RetIndex:1122
CompName:Resorcinol $$ 1,3-Benzenediol $$ .alpha.-Resorcinol $$ m-Benzenediol $$ m-Dihydroxybenzene $$ m-Dioxybenzene $$ m-Hydroquinone $$

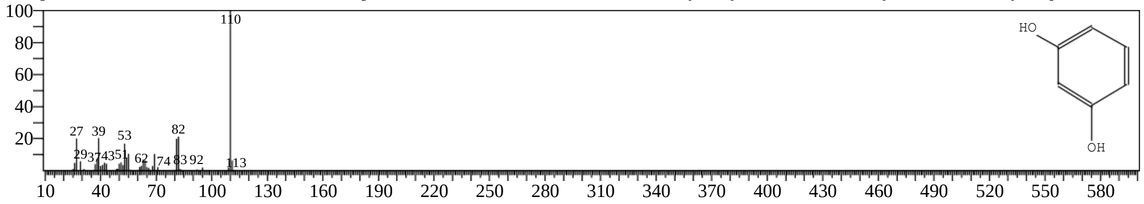

Hit#:3 Entry:3258 Library:NIST20M1.lib
SI:94 Formula:C6H6O2 CAS:108-46-3 MolWeight:110 RetIndex:1122
CompName:Resorcinol $$ 1,3-Benzenediol $$ .alpha.-Resorcinol $$ m-Benzenediol $$ m-Dihydroxybenzene $$ m-Dioxybenzene $$ m-Hydroquinone $$

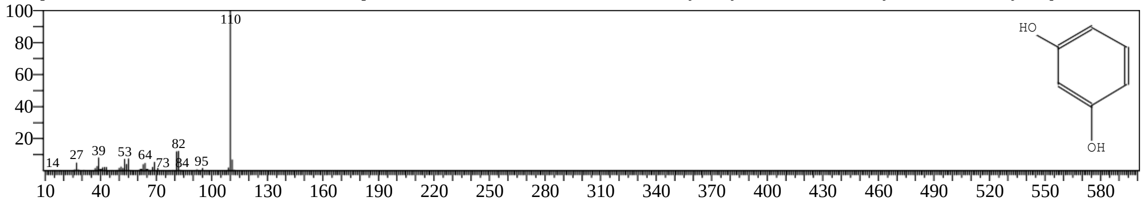

Hit#:4 Entry:3231 Library:NIST20R.lib
SI:92 Formula:C6H6O2 CAS:123-31-9 MolWeight:110 RetIndex:1122
CompName:Hydroquinone $$ 1,4-Benzenediol $$ p-Benzenediol $$ p-Dihydroxybenzene $$ p-Dioxybenzene $$ p-Hydroquinone $$ p-Hydroxyphenol $$

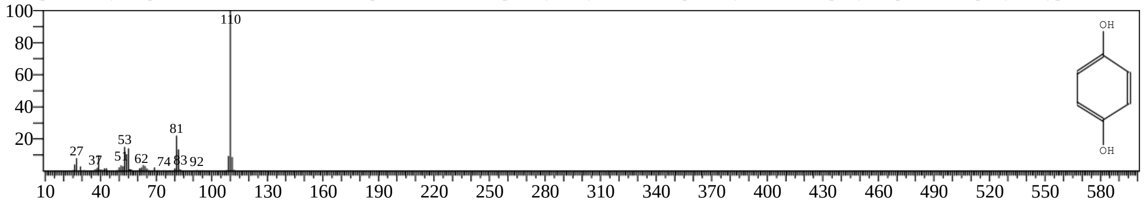

Hit#:5 Entry:3220 Library:NIST20R.lib
SI:91 Formula:C6H6O2 CAS:108-46-3 MolWeight:110 RetIndex:1122
CompName:Resorcinol $$ 1,3-Benzenediol $$ .alpha.-Resorcinol $$ m-Benzenediol $$ m-Dihydroxybenzene $$ m-Dioxybenzene $$ m-Hydroquinone $$

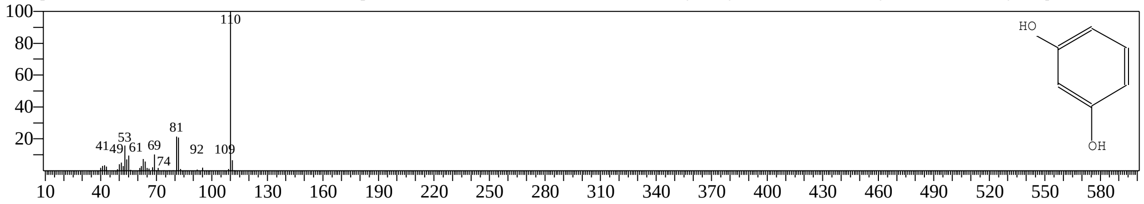

<< Target >>
Line#:7 R.Time:14.350(Scan#:712) MassPeaks:367
RawMode:Averaged 14.333-14.367(711-713) BasePeak:154.05(470494)
BG Mode:Calc. from Peak Group 1 - Event 1 Scan

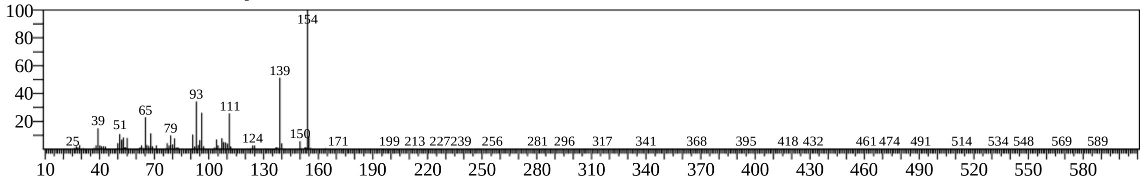

Hit#:1 Entry:12178 Library:NIST20R.lib
SI:93 Formula:C8H10O3 CAS:91-10-1 MolWeight:154 RetIndex:1279
CompName:Phenol, 2,6-dimethoxy- $$ Pyrogallol 1,3-dimethyl ether $$ Syringol $$ 1,3-Dimethoxy-2-hydroxybenzene $$ 2-Hydroxy-1,3-dimethoxybenzen

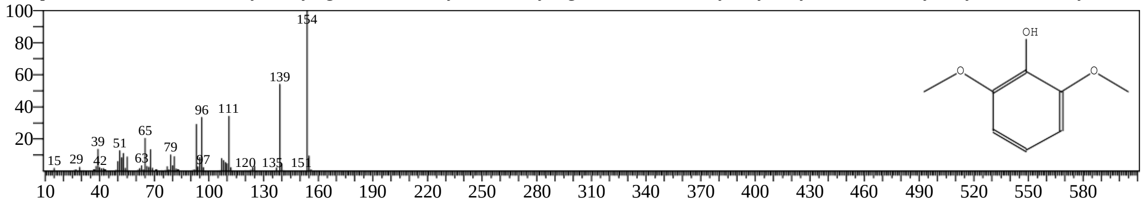

Hit#:2 Entry:12179 Library:NIST20R.lib
SI:91 Formula:C8H10O3 CAS:91-10-1 MolWeight:154 RetIndex:1279
CompName:Phenol, 2,6-dimethoxy- $$ Pyrogallol 1,3-dimethyl ether $$ Syringol $$ 1,3-Dimethoxy-2-hydroxybenzene $$ 2-Hydroxy-1,3-dimethoxybenzen

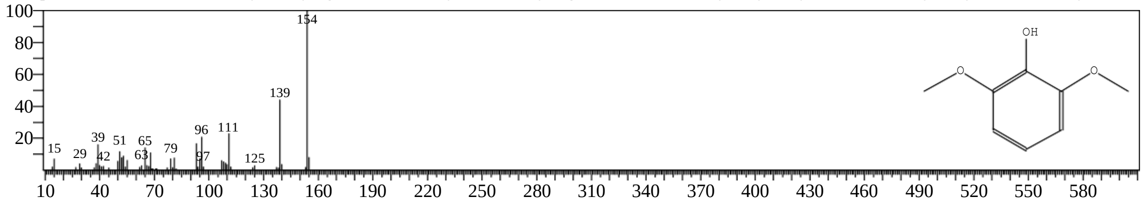

Hit#:3 Entry:19192 Library:NIST20M1.lib
SI:91 Formula:C8H10O3 CAS:91-10-1 MolWeight:154 RetIndex:1279
CompName:Phenol, 2,6-dimethoxy- $$ Pyrogallol 1,3-dimethyl ether $$ Syringol $$ 1,3-Dimethoxy-2-hydroxybenzene $$ 2-Hydroxy-1,3-dimethoxybenzen

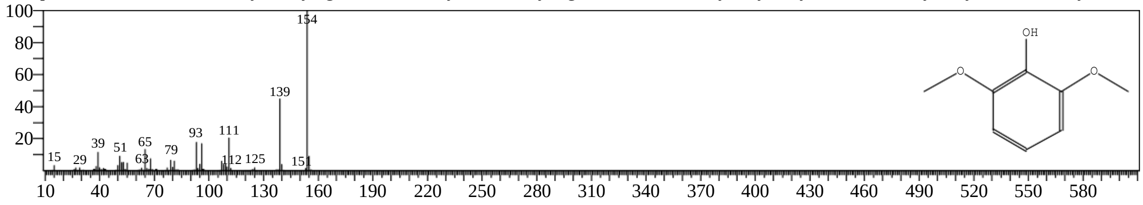

Hit#:4 Entry:19211 Library:NIST20M1.lib
SI:89 Formula:C8H10O3 CAS:5150-42-5 MolWeight:154 RetIndex:1279
CompName:2,3-Dimethoxyphenol $$ Phenol, 2,3-dimethoxy- $$ 1-Hydroxy-2,3-dimethoxybenzene

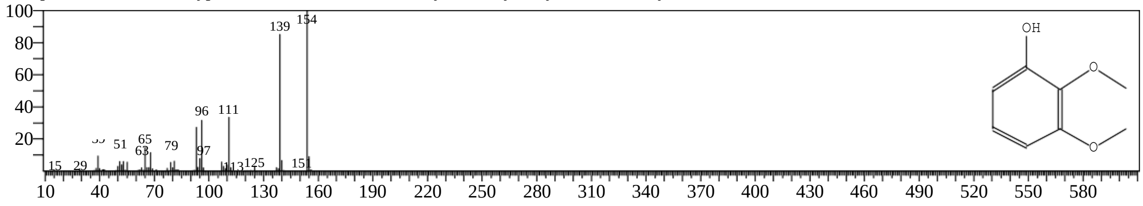

Hit#:5 Entry:12185 Library:NIST20R.lib
SI:85 Formula:C8H10O3 CAS:2033-89-8 MolWeight:154 RetIndex:1279
CompName:Phenol, 3,4-dimethoxy- $$ 3,4-Dimethoxyphenol

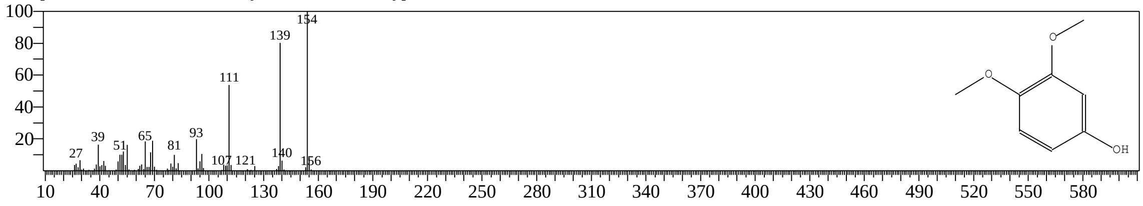

<< Target >>
Line#:8 R.Time:14.450(Scan#:718) MassPeaks:289
RawMode:Averaged 14.433-14.467(717-719) BasePeak:91.10(41635)
BG Mode:Calc. from Peak Group 1 - Event 1 Scan

After the flowers have

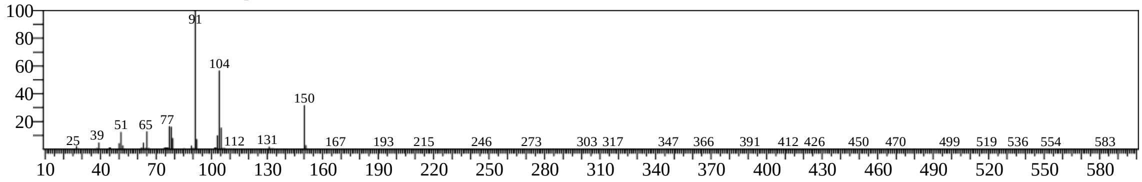

Hit#:1 Entry:16997 Library:NIST20M1.lib
SI:96 Formula:C9H10O2 CAS:501-52-0 MolWeight:150 RetIndex:1349
CompName:Hydrocinnamic acid $$ Benzenepropanoic acid $$ .beta.-Phenylpropionic acid $$ Benzenepropionic acid $$ Benzylacetic acid $$ Dihydrocinna

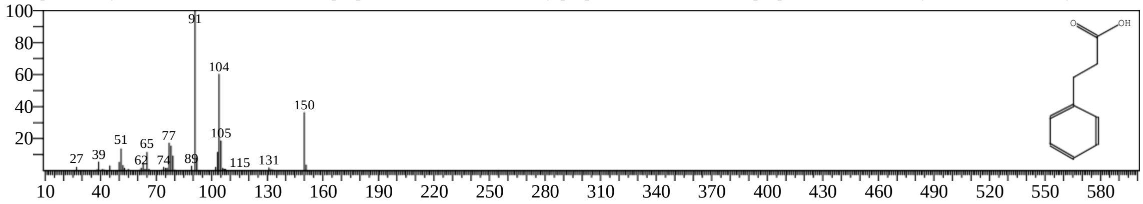

Hit#:2 Entry:11031 Library:NIST20R.lib
SI:95 Formula:C9H10O2 CAS:501-52-0 MolWeight:150 RetIndex:1349
CompName:Hydrocinnamic acid $$ Benzenepropanoic acid $$ .beta.-Phenylpropionic acid $$ Benzenepropionic acid $$ Benzylacetic acid $$ Dihydrocinna

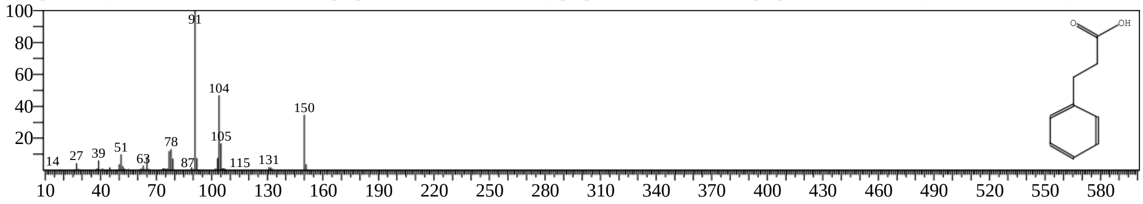

Hit#:3 Entry:11032 Library:NIST20R.lib
SI:95 Formula:C9H10O2 CAS:501-52-0 MolWeight:150 RetIndex:1349
CompName:Hydrocinnamic acid $$ Benzenepropanoic acid $$ .beta.-Phenylpropionic acid $$ Benzenepropionic acid $$ Benzylacetic acid $$ Dihydrocinna

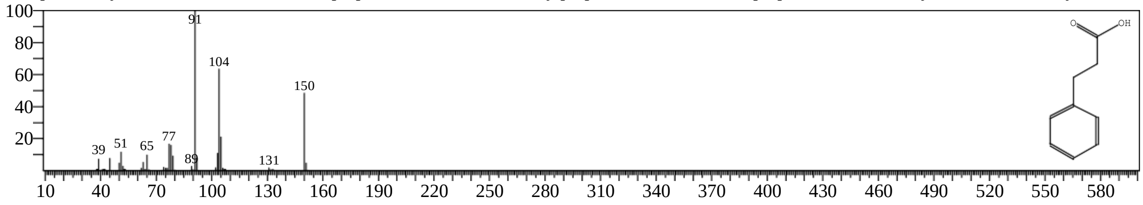

Hit#:4 Entry:11030 Library:NIST20R.lib
SI:95 Formula:C9H10O2 CAS:501-52-0 MolWeight:150 RetIndex:1349
CompName:Hydrocinnamic acid $$ Benzenepropanoic acid $$ .beta.-Phenylpropionic acid $$ Benzenepropionic acid $$ Benzylacetic acid $$ Dihydrocinna

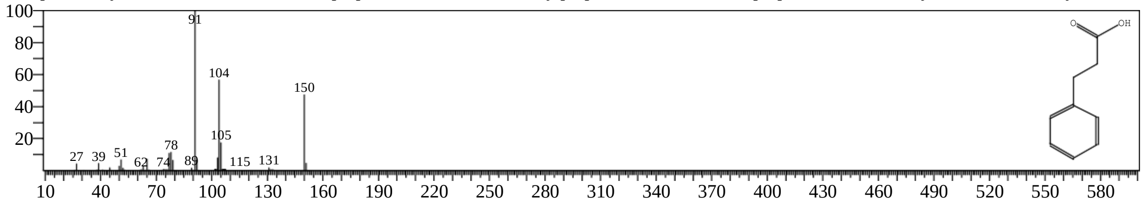

Hit#:5 Entry:11029 Library:NIST20R.lib
SI:93 Formula:C9H10O2 CAS:501-52-0 MolWeight:150 RetIndex:1349
CompName:Hydrocinnamic acid $$ Benzenepropanoic acid $$ .beta.-Phenylpropionic acid $$ Benzenepropionic acid $$ Benzylacetic acid $$ Dihydrocinna

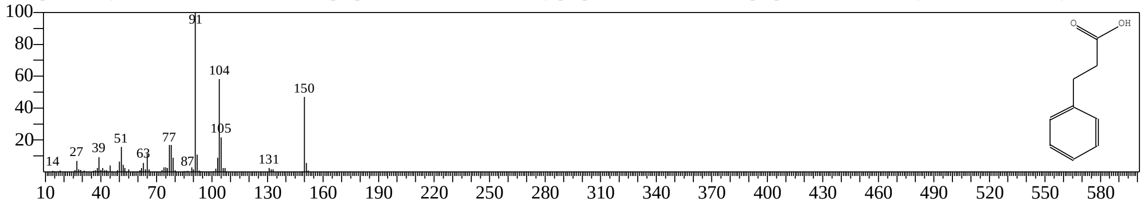

<< Target >>
Line#:9 R.Time:14.617(Scan#:728) MassPeaks:303
RawMode:Averaged 14.600-14.633(727-729) BasePeak:73.05(22314)
BG Mode:Calc. from Peak Group 1 - Event 1 Scan

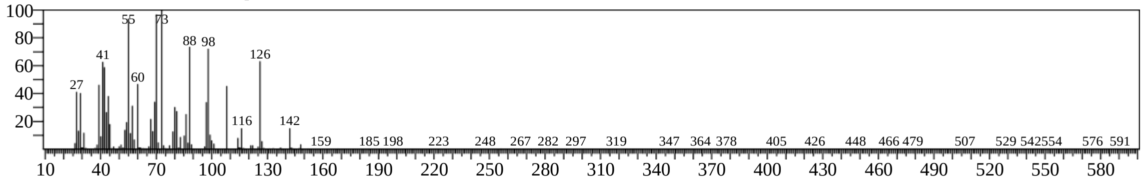

Hit#:1 Entry:14216 Library:NIST20M1.lib
SI:91 Formula:C7H12O3 CAS:3685-26-5 MolWeight:144 RetIndex:1325
CompName:trans-4-Hydroxycyclohexanecarboxylic acid

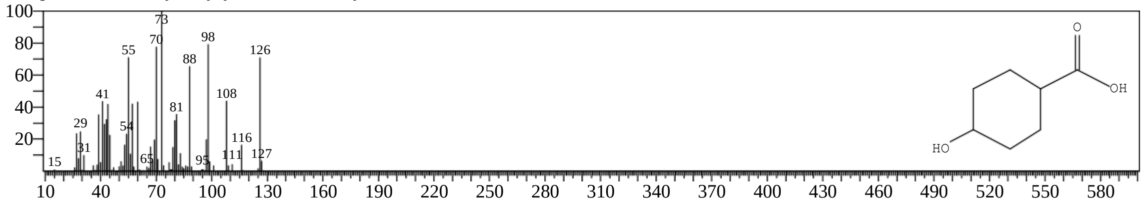

Hit#:2 Entry:14215 Library:NIST20M1.lib
SI:87 Formula:C7H12O3 CAS:3685-22-1 MolWeight:144 RetIndex:1325
CompName:cis-4-Hydroxycyclohexanecarboxylic acid

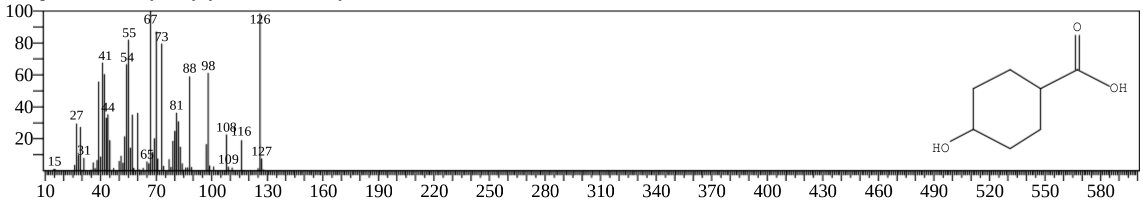

Hit#:3 Entry:14217 Library:NIST20M1.lib
SI:84 Formula:C7H12O3 CAS:17419-81-7 MolWeight:144 RetIndex:1325
CompName:4-Hydroxycyclohexanecarboxylic acid,c&t

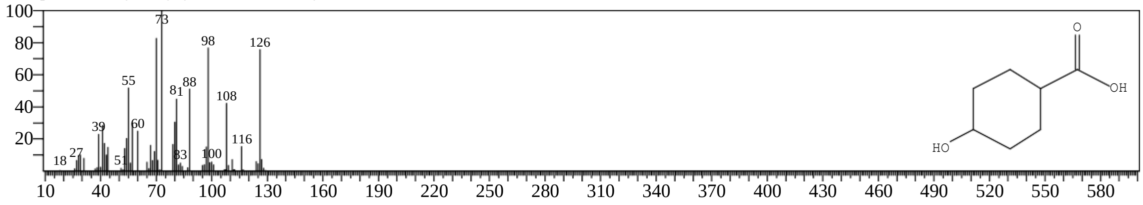

Hit#:4 Entry:4911 Library:NIST20M1.lib
SI:76 Formula:C6H12O2 CAS:823-18-7 MolWeight:116 RetIndex:1096
CompName:1,3-Cyclohexanediol, cis- $$ cis-1,3-Cyclohexanediol $$ 1,3-Cyclohexanediol, (Z)- $$ cis-1,3-Cyclohexandiol

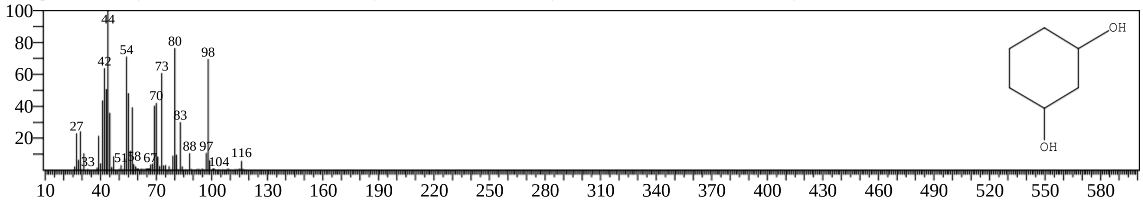

Hit#:5 Entry:4893 Library:NIST20M1.lib
SI:75 Formula:C6H12O2 CAS:73959-42-9 MolWeight:116 RetIndex:999
CompName:1-(1-Hydroxyethyl),1-(hydroxymethyl)cyclopropane $$ 1-[1-(Hydroxymethyl)cyclopropyl]ethanol #

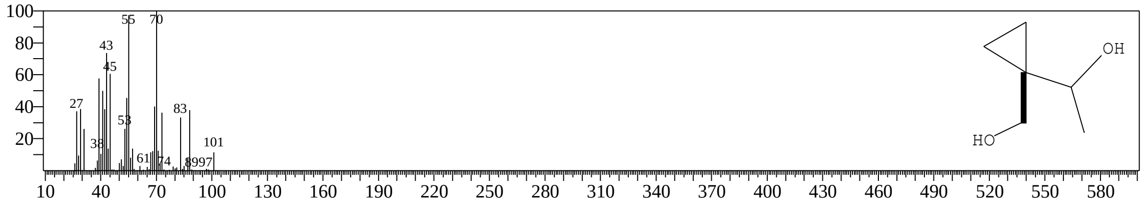

<< Target >>
Line#:10 R.Time:15.167(Scan#:761) MassPeaks:334
RawMode:Averaged 15.150-15.183(760-762) BasePeak:92.10(42013)
BG Mode:Calc. from Peak Group 1 - Event 1 Scan

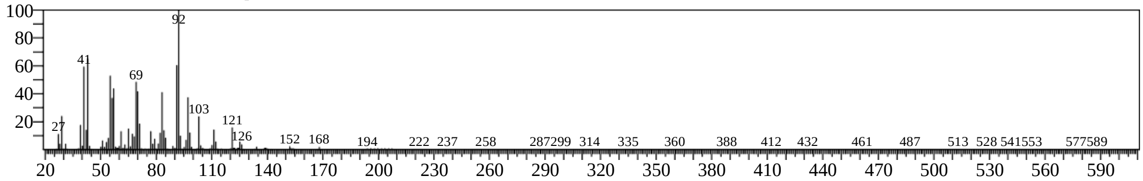

Hit#:1 Entry:147133 Library:NIST20M1.lib
SI:83 Formula:C17H34O3 CAS:0-00-0 MolWeight:286 RetIndex:1954
CompName:Carbonic acid, ethyl tetradecyl ester

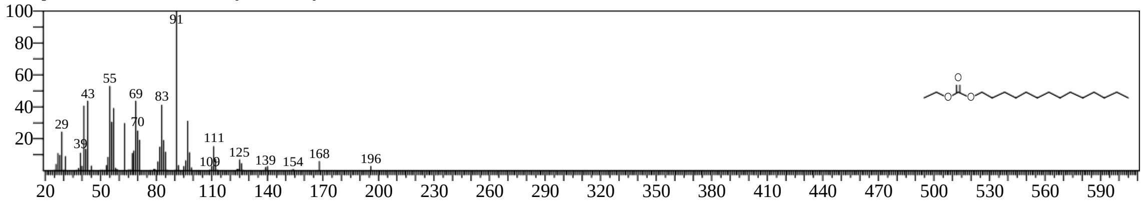

Hit#:2 Entry:114264 Library:NIST20M1.lib
SI:83 Formula:C15H30O3 CAS:0-00-0 MolWeight:258 RetIndex:1755
CompName:Carbonic acid, dodecyl ethyl ester

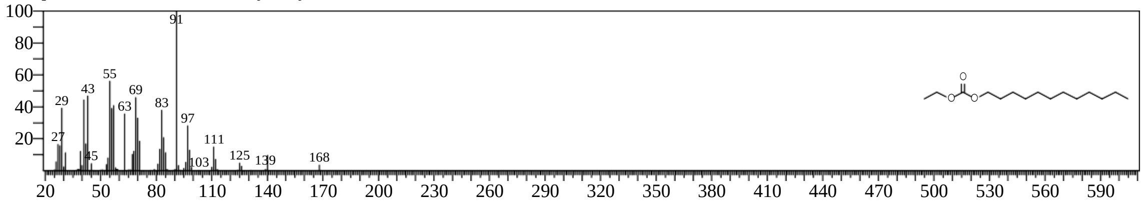

Hit#:3 Entry:83487 Library:NIST20M1.lib
SI:83 Formula:C13H26O3 CAS:0-00-0 MolWeight:230 RetIndex:1557
CompName:Carbonic acid, decyl ethyl ester

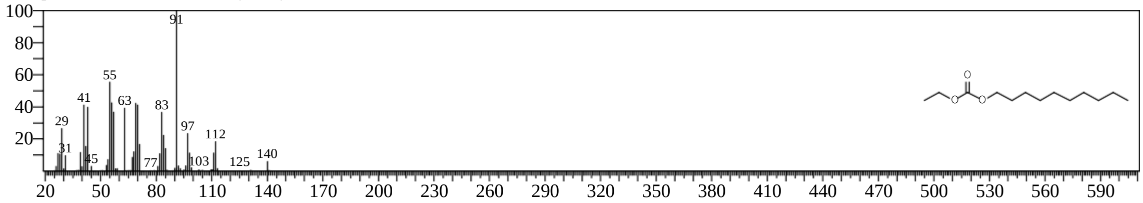

Hit#:4 Entry:130578 Library:NIST20M1.lib
SI:83 Formula:C16H32O3 CAS:0-00-0 MolWeight:272 RetIndex:1855
CompName:Carbonic acid, ethyl tridecyl ester

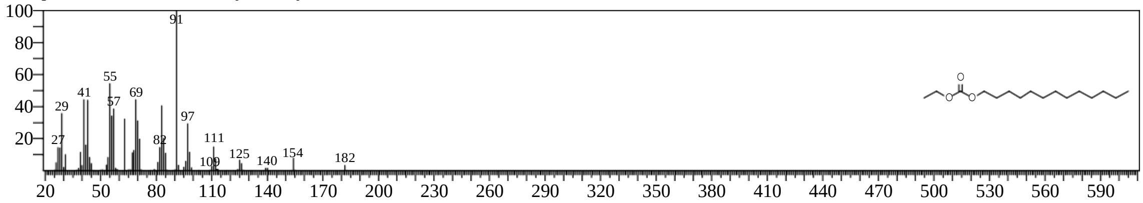

Hit#:5 Entry:168308 Library:NIST20M1.lib
SI:80 Formula:C17H33ClO2 CAS:0-00-0 MolWeight:304 RetIndex:2039
CompName:3-Chloropropionic acid, 3-tetradecyl ester $$ 1-Ethyldodecyl 3-chloropropanoate #

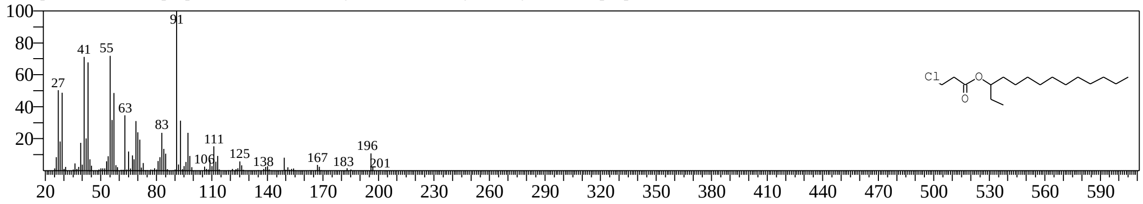

<< Target >>
Line#:11 R.Time:16.867(Scan#:863) MassPeaks:340
RawMode:Averaged 16.850-16.883(862-864) BasePeak:123.10(21938)
BG Mode:Calc. from Peak Group 1 - Event 1 Scan

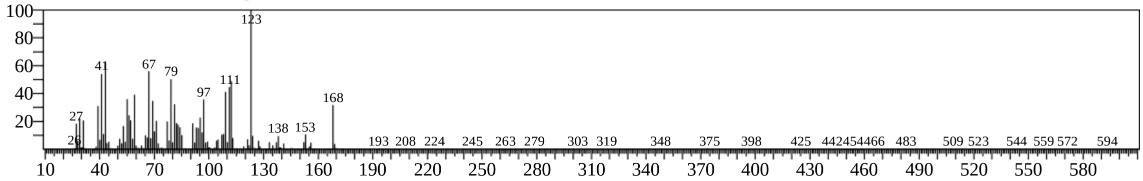

Hit#:1 Entry:28082 Library:NIST20M1.lib
SI:78 Formula:C10H16O2 CAS:0-00-0 MolWeight:168 RetIndex:1351
CompName:2-Ethyl-1,3-dimethylcyclopent-2-enecarboxylic acid $$ 2-Ethyl-1,3-dimethyl-2-cyclopentene-1-carboxylic acid #

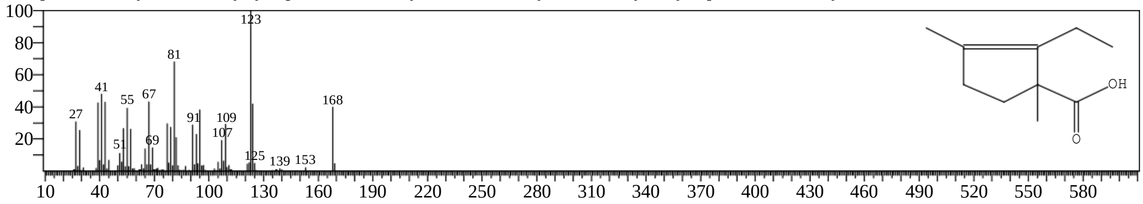

Hit#:2 Entry:205575 Library:NIST20M1.lib
SI:78 Formula:C22H40O2 CAS:69502-96-1 MolWeight:336 RetIndex:2453
CompName:2H-Pyran, 2-(2-heptadecynyloxy)tetrahydro- $$ 2-(2-Heptadecynyloxy)tetrahydro-2H-pyran #

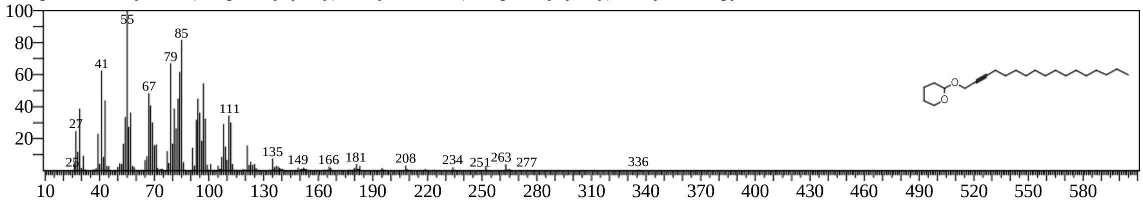

Hit#:3 Entry:28275 Library:NIST20M1.lib
SI:78 Formula:C10H16O2 CAS:705-16-8 MolWeight:168 RetIndex:1256
CompName:dl-trans-Chrysanthemic acid $$ Cyclopropanecarboxylic acid, 2,2-dimethyl-3-(2-methyl-1-propenyl)-, trans-(.+/-.)- $$ (.+/-.)-trans-Chrysanthem

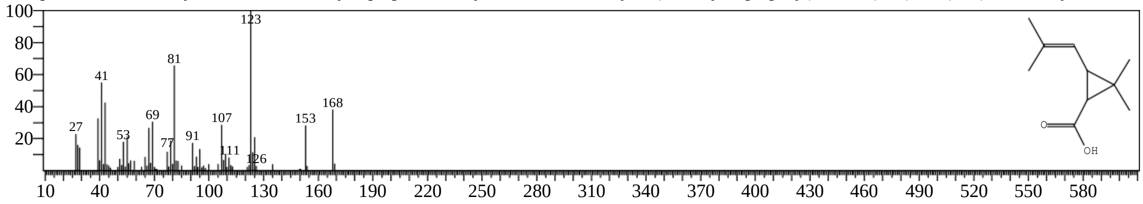

Hit#:4 Entry:28277 Library:NIST20M1.lib
SI:77 Formula:C10H16O2 CAS:10453-89-1 MolWeight:168 RetIndex:1256
CompName:Chrysanthemic acid $$ Cyclopropanecarboxylic acid, 2,2-dimethyl-3-(2-methyl-1-propenyl)- $$ Cyclopropanecarboxylic acid, 2,2-dimethyl-3-(

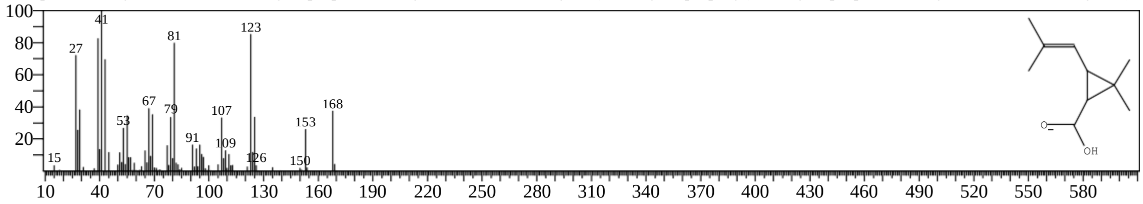

Hit#:5 Entry:12305 Library:NIST20R.lib
SI:77 Formula:C10H18O CAS:18383-58-9 MolWeight:154 RetIndex:1143
CompName:trans-Chrysanthemol $$ [2,2-Dimethyl-3-(2-methyl-1-propenyl)cyclopropyl]methanol #

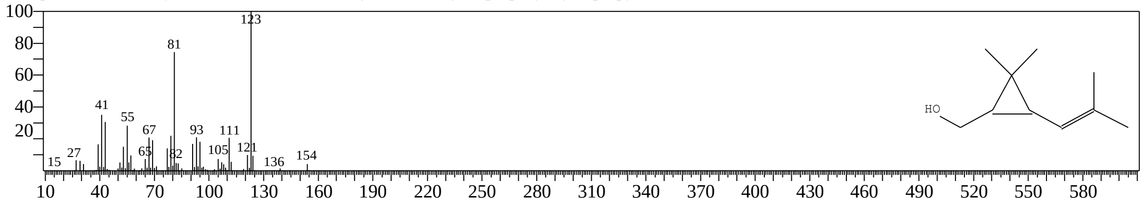

<< Target >>
Line#:12 R.Time:18.150(Scan#:940) MassPeaks:319
RawMode:Averaged 18.133-18.167(939-941) BasePeak:107.10(38967)
BG Mode:Calc. from Peak Group 1 - Event 1 Scan

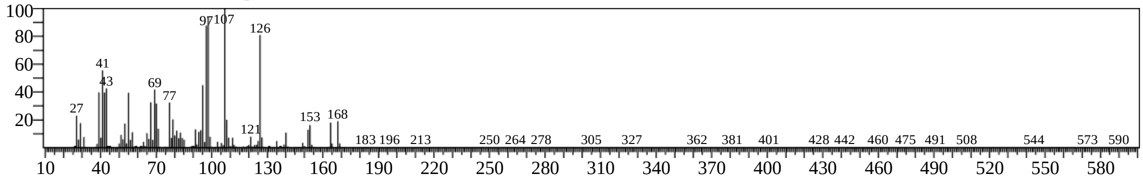

Hit#:1 Entry:28134 Library:NIST20M1.lib
SI:86 Formula:C10H16O2 CAS:55955-54-9 MolWeight:168 RetIndex:1401
CompName:2-Cyclohexen-1-one, 3-(hydroxymethyl)-6-(1-methylethyl)- $$ 3-(Hydroxymethyl)-6-isopropyl-2-cyclohexen-1-one #

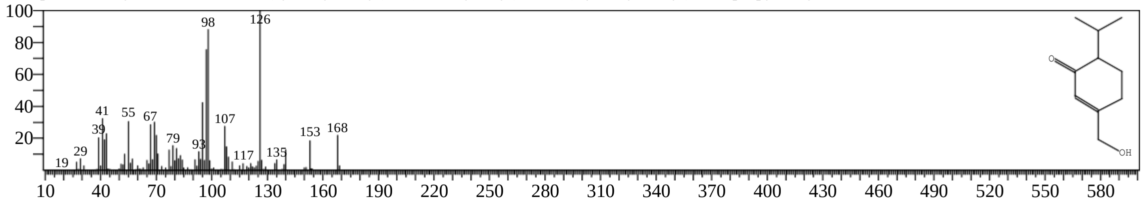

Hit#:2 Entry:15631 Library:NIST20R.lib
SI:83 Formula:C10H16O2 CAS:55955-54-9 MolWeight:168 RetIndex:1401
CompName:2-Cyclohexen-1-one, 3-(hydroxymethyl)-6-(1-methylethyl)- $$ 3-(Hydroxymethyl)-6-isopropyl-2-cyclohexen-1-one #

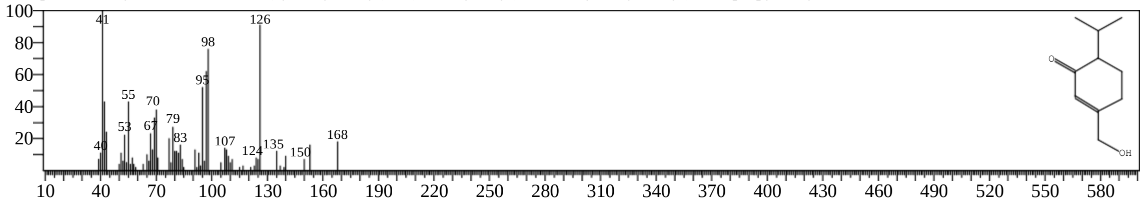

Hit#:3 Entry:28204 Library:NIST20M1.lib
SI:77 Formula:C10H16O2 CAS:490-03-9 MolWeight:168 RetIndex:1288
CompName:2-Cyclohexen-1-one, 2-hydroxy-3-methyl-6-(1-methylethyl)- $$ Diosphenol $$ Barosma camphor $$ Buccocamphor $$ Buchu camphor $$ 2-H

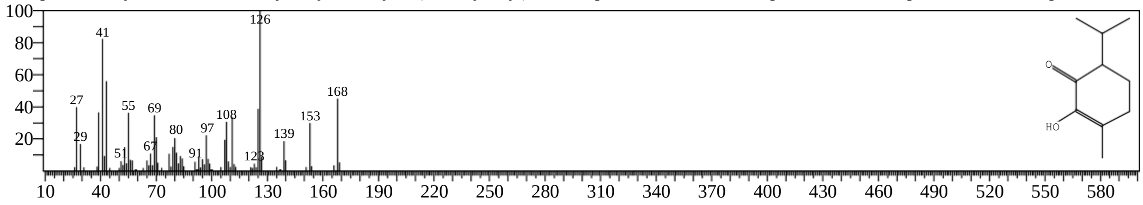

Hit#:4 Entry:5917 Library:NIST20R.lib
SI:77 Formula:C8H14O CAS:645-62-5 MolWeight:126 RetIndex:990
CompName:2-Hexenal, 2-ethyl- $$ .alpha.-Ethyl-.beta.-propylacrolein $$ 2-Ethyl-2-hexen-1-al $$ 2-Ethyl-2-hexenal $$ 2-Ethyl-3-propylacrolein $$ .alpha.-

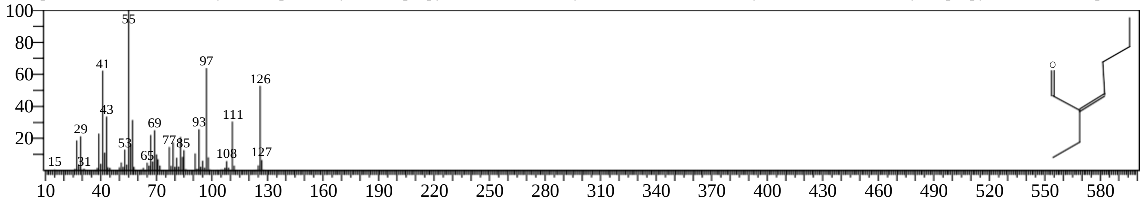

Hit#:5 Entry:28441 Library:NIST20M1.lib
SI:76 Formula:C11H20O CAS:61203-83-6 MolWeight:168 RetIndex:1349
CompName:4-Pentylcyclohexanone

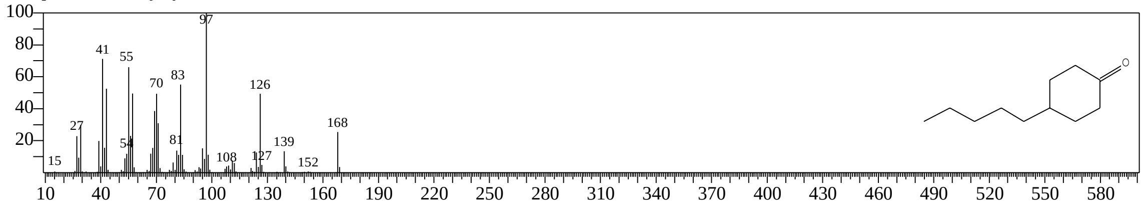

<< Target >>
Line#:13 R.Time:18.833(Scan#:981) MassPeaks:343
RawMode:Averaged 18.817-18.850(980-982) BasePeak:43.10(101059)
BG Mode:Calc. from Peak Group 1 - Event 1 Scan

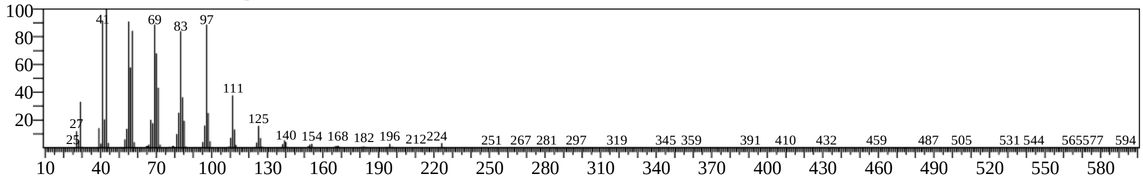

Hit#:1 Entry:24890 Library:NIST20R.lib
SI:96 Formula:C15H30 CAS:13360-61-7 MolWeight:210 RetIndex:1502
CompName:1-Pentadecene $$ Pentadecene,1- $$ Pentadec-1-ene

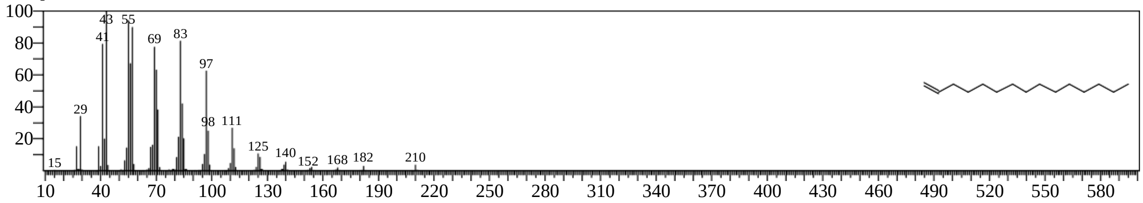

Hit#:2 Entry:29353 Library:NIST20R.lib
SI:96 Formula:C17H34 CAS:6765-39-5 MolWeight:238 RetIndex:1701
CompName:1-Heptadecene $$ Hexahydroaplotaxene $$ Heptadec-1-ene $$ NSC 77132 $$ Heptadecene (Related)

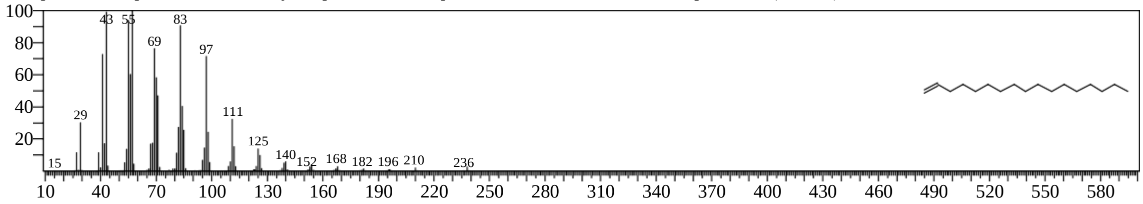

Hit#:3 Entry:107593 Library:NIST20M1.lib
SI:96 Formula:C18H36 CAS:112-88-9 MolWeight:252 RetIndex:1801
CompName:1-Octadecene $$ .alpha.-Octadecene $$ Octadecylene, .alpha.- $$ Neodene 18 $$ Octadec-1-ene $$ Octadecene-1 $$ Octadeca-1-ene

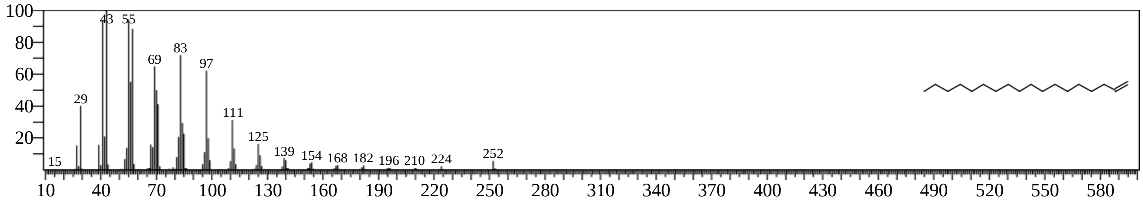

Hit#:4 Entry:107601 Library:NIST20M1.lib
SI:96 Formula:C18H36 CAS:7206-19-1 MolWeight:252 RetIndex:1818
CompName:3-Octadecene, (E)- $$ (3E)-3-Octadecene $$ (E)-3-Octadecene $$ trans-3-Octadecene

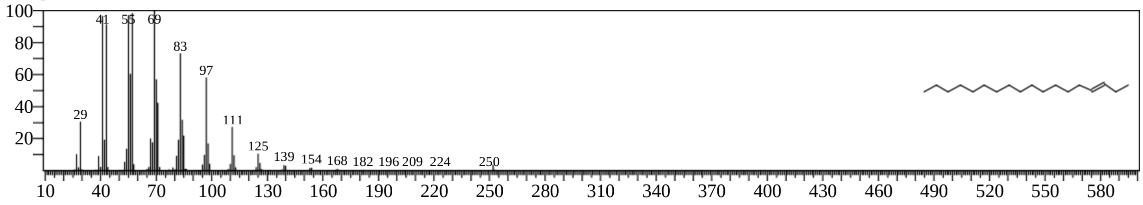

Hit#:5 Entry:27375 Library:NIST20R.lib
SI:95 Formula:C16H32 CAS:629-73-2 MolWeight:224 RetIndex:1602
CompName:Cetene $$ 1-Hexadecene $$ .alpha.-Hexadecene $$ n-Hexadec-1-ene $$ 1-Cetene $$ Hexadecylene-1 $$ Hexadec-1-ene $$ Hexadecene-1 $$ N

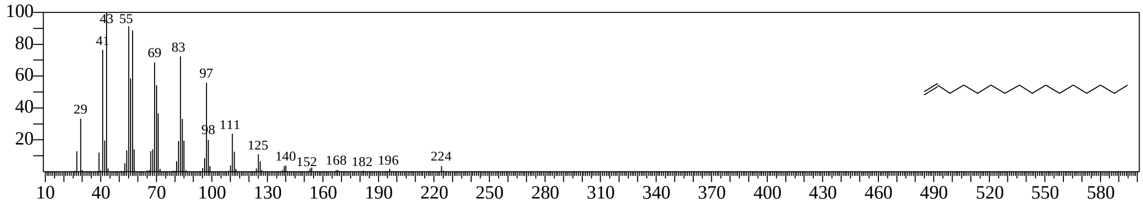

<< Target >>
Line#:14 R.Time:19.750(Scan#:1036) MassPeaks:376
RawMode:Averaged 19.733-19.767(1035-1037) BasePeak:107.10(159234)
BG Mode:Calc. from Peak Group 1 - Event 1 Scan

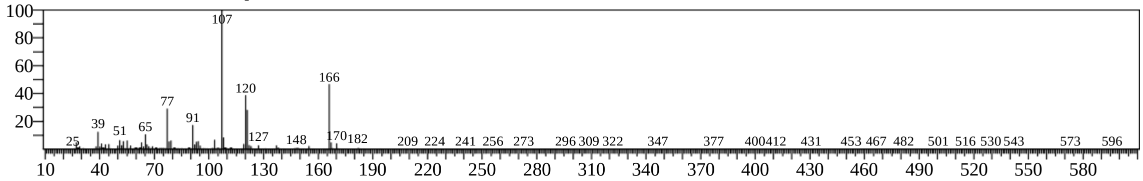

Hit#:1 Entry:15068 Library:NIST20R.lib
SI:88 Formula:C9H10O3 CAS:501-97-3 MolWeight:166 RetIndex:1569
CompName:Benzenepropanoic acid, 4-hydroxy- $$ Phloretic acid $$ Hydrocinnamic acid, p-hydroxy- $$ .beta.-(p-Hydroxyphenyl)propionic acid $$ p-Hydr

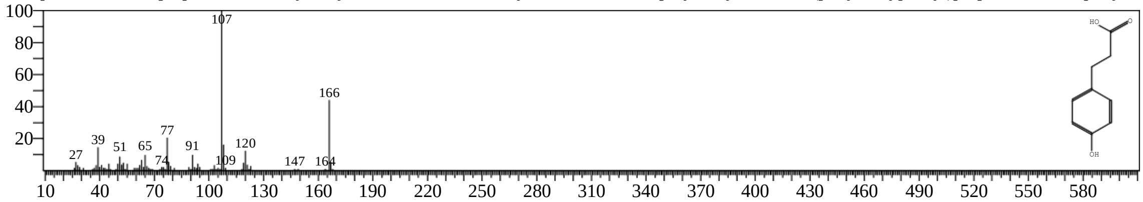

Hit#:2 Entry:15095 Library:NIST20R.lib
SI:84 Formula:C9H10O3 CAS:621-54-5 MolWeight:166 RetIndex:1569
CompName:3-(3-Hydroxyphenyl)propionic acid $$ .beta.-(m-Hydroxyphenyl)propionic acid $$ Benzenepropanoic acid, 3-hydroxy- $$ .beta.-(3-Hydroxyph

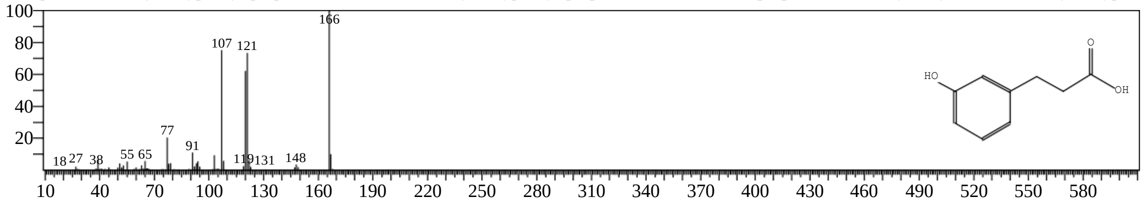

Hit#:3 Entry:26604 Library:NIST20M1.lib
SI:81 Formula:C9H10O3 CAS:501-97-3 MolWeight:166 RetIndex:1569
CompName:Benzenepropanoic acid, 4-hydroxy- $$ Phloretic acid $$ Hydrocinnamic acid, p-hydroxy- $$ .beta.-(p-Hydroxyphenyl)propionic acid $$ p-Hydr

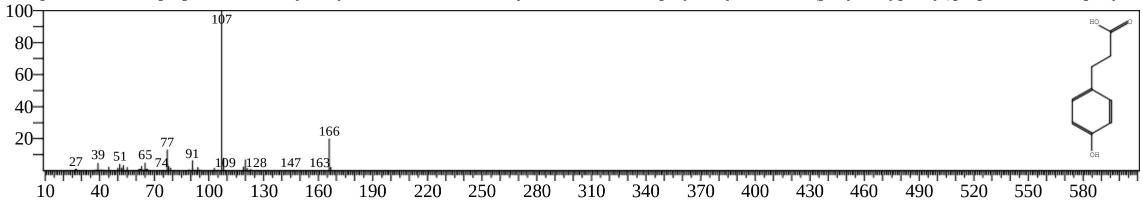

Hit#:4 Entry:15069 Library:NIST20R.lib
SI:80 Formula:C9H10O3 CAS:501-97-3 MolWeight:166 RetIndex:1569
CompName:Benzenepropanoic acid, 4-hydroxy- $$ Phloretic acid $$ Hydrocinnamic acid, p-hydroxy- $$ .beta.-(p-Hydroxyphenyl)propionic acid $$ p-Hydr

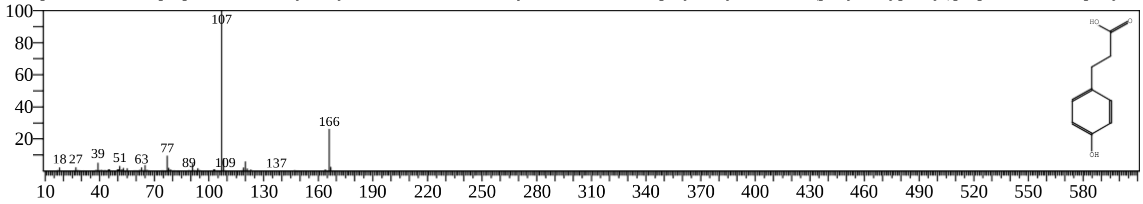

Hit#:5 Entry:15093 Library:NIST20R.lib
SI:80 Formula:C9H10O3 CAS:1878-49-5 MolWeight:166 RetIndex:1439
CompName:Acetic acid, (2-methylphenoxy)- $$ 2-Methylphenoxyacetic acid $$ ortho-Tolyloxyacetic acid $$ o-Methyl phenoxy acetic acid $$ Acetic acid, (

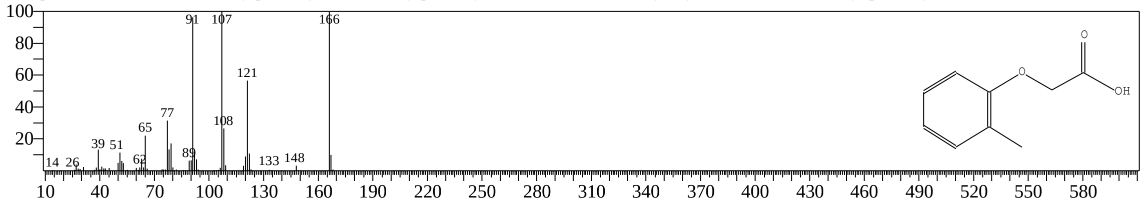

<< Target >>
Line#:15 R.Time:21.617(Scan#:1148) MassPeaks:332
RawMode:Averaged 21.600-21.633(1147-1149) BasePeak:73.05(86063)
BG Mode:Calc. from Peak Group 1 - Event 1 Scan

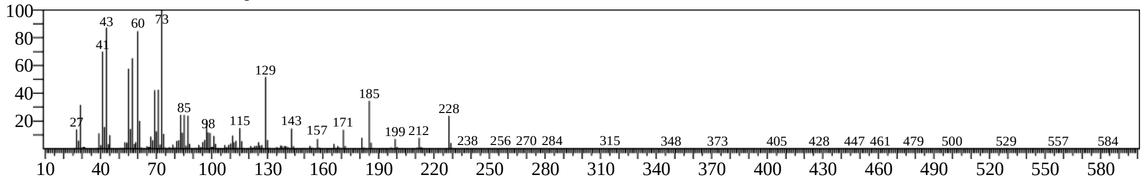

Hit#:1 Entry:81713 Library:NIST20M1.lib
SI:95 Formula:C14H28O2 CAS:544-63-8 MolWeight:228 RetIndex:1769
CompName:Tetradecanoic acid $$ Myristic acid $$ n-Tetradecanoic acid $$ n-Tetradecoic acid $$ Neo-Fat 14 $$ Univol U 316S $$ 1-Tridecanecarboxylic

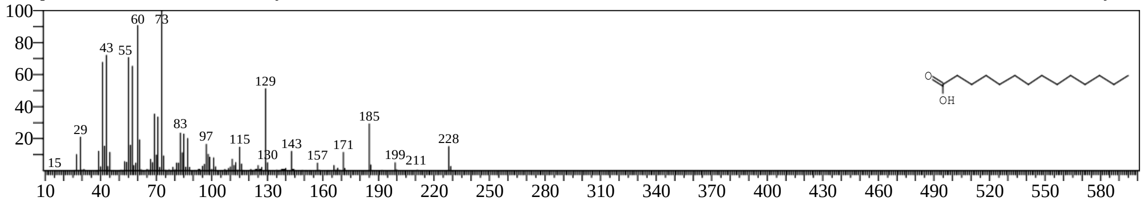

Hit#:2 Entry:28009 Library:NIST20R.lib
SI:94 Formula:C14H28O2 CAS:544-63-8 MolWeight:228 RetIndex:1769
CompName:Tetradecanoic acid $$ Myristic acid $$ n-Tetradecanoic acid $$ n-Tetradecoic acid $$ Neo-Fat 14 $$ Univol U 316S $$ 1-Tridecanecarboxylic

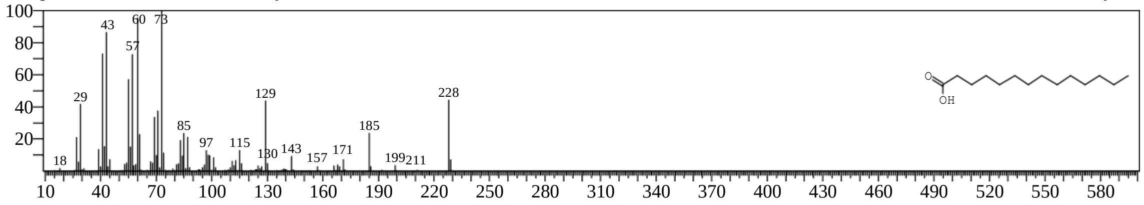

Hit#:3 Entry:28011 Library:NIST20R.lib
SI:93 Formula:C14H28O2 CAS:544-63-8 MolWeight:228 RetIndex:1769
CompName:Tetradecanoic acid $$ Myristic acid $$ n-Tetradecanoic acid $$ n-Tetradecoic acid $$ Neo-Fat 14 $$ Univol U 316S $$ 1-Tridecanecarboxylic

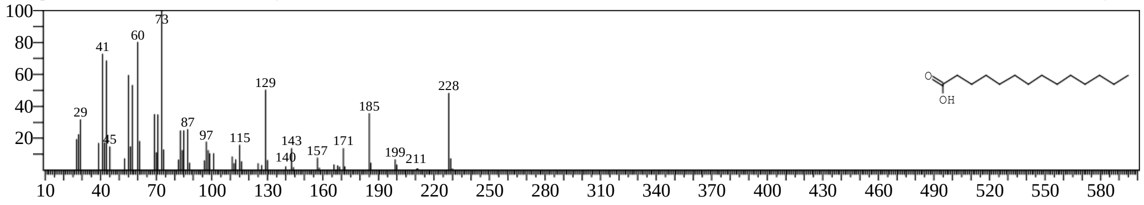

Hit#:4 Entry:96603 Library:NIST20M1.lib
SI:93 Formula:C15H30O2 CAS:1002-84-2 MolWeight:242 RetIndex:1869
CompName:Pentadecanoic acid $$ Pentadecylic acid $$ n-Pentadecanoic acid $$ n-Pentadecylic acid

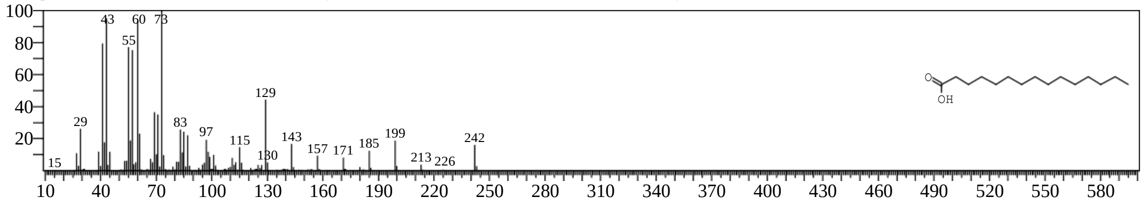

Hit#:5 Entry:29890 Library:NIST20R.lib
SI:92 Formula:C15H30O2 CAS:1002-84-2 MolWeight:242 RetIndex:1869
CompName:Pentadecanoic acid $$ Pentadecylic acid $$ n-Pentadecanoic acid $$ n-Pentadecylic acid

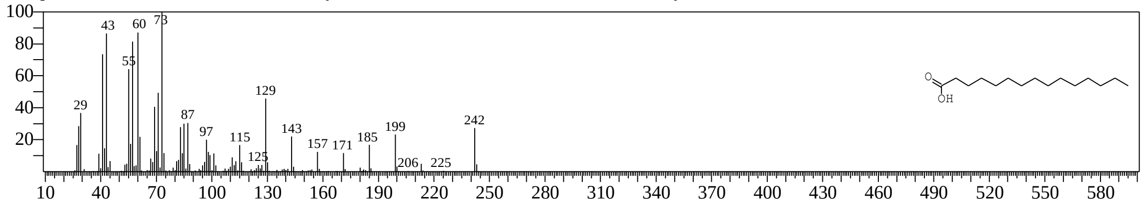

<< Target >>
Line#:16 R.Time:22.050(Scan#:1174) MassPeaks:410
RawMode:Averaged 22.033-22.067(1173-1175) BasePeak:97.15(122363)
BG Mode:Calc. from Peak Group 1 - Event 1 Scan

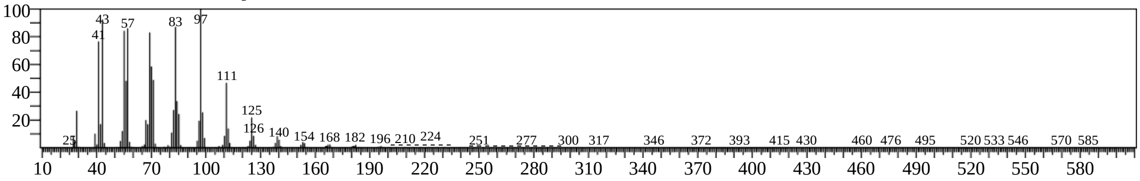

Hit#:1 Entry:32651 Library:NIST20R.lib
SI:98 Formula:C19H38 CAS:18435-45-5 MolWeight:266 RetIndex:1900
CompName:1-Nonadecene $$ Nonadec-1-ene

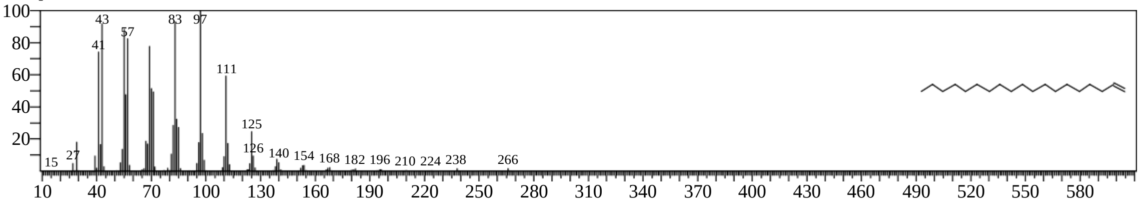

Hit#:2 Entry:29353 Library:NIST20R.lib
SI:96 Formula:C17H34 CAS:6765-39-5 MolWeight:238 RetIndex:1701
CompName:1-Heptadecene $$ Hexahydroaplotaxene $$ Heptadec-1-ene $$ NSC 77132 $$ Heptadecene (Related)

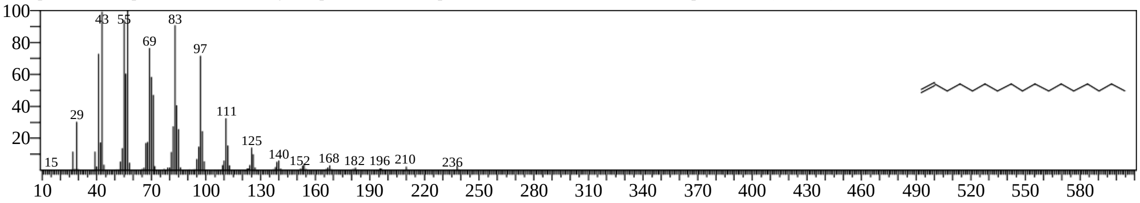

Hit#:3 Entry:38649 Library:NIST20R.lib
SI:96 Formula:C24H48 CAS:10192-32-2 MolWeight:336 RetIndex:2397
CompName:1-Tetracosene

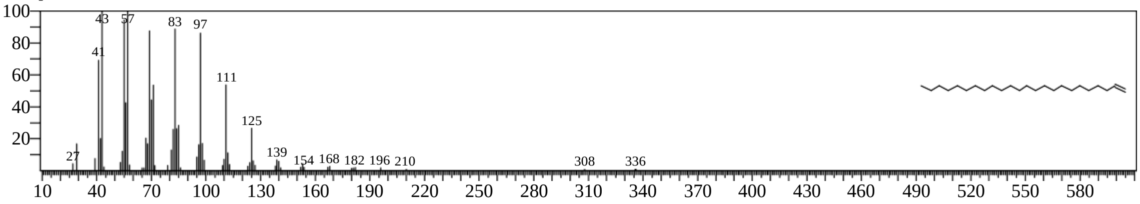

Hit#:4 Entry:40183 Library:NIST20R.lib
SI:95 Formula:C26H52 CAS:18835-33-1 MolWeight:364 RetIndex:2596
CompName:1-Hexacosene

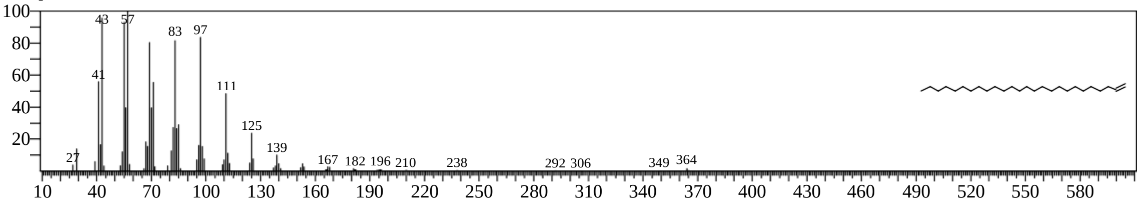

Hit#:5 Entry:31116 Library:NIST20R.lib
SI:95 Formula:C18H36 CAS:112-88-9 MolWeight:252 RetIndex:1801
CompName:1-Octadecene $$ .alpha.-Octadecene $$ Octadecylene, .alpha.- $$ Neodene 18 $$ Octadec-1-ene $$ Octadecene-1 $$ Octadeca-1-ene

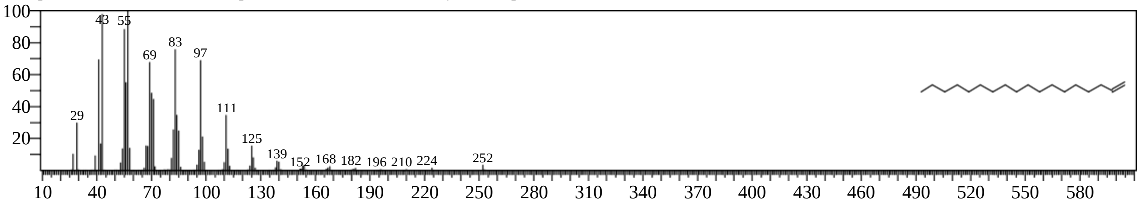

<< Target >>
Line#:17 R.Time:22.283(Scan#:1188) MassPeaks:327
RawMode:Averaged 22.267-22.300(1187-1189) BasePeak:206.05(9820)
BG Mode:Calc. from Peak Group 1 - Event 1 Scan

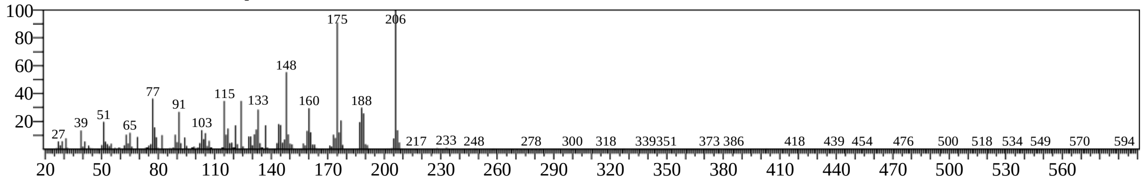

Hit#:1 Entry:58934 Library:NIST20M1.lib
SI:66 Formula:C13H18O2 CAS:58866-66-3 MolWeight:206 RetIndex:1488
CompName:7,7-Dimethoxy-2,3,4,5,6,7-hexahydro-1H-cyclopenta[a]pentalene

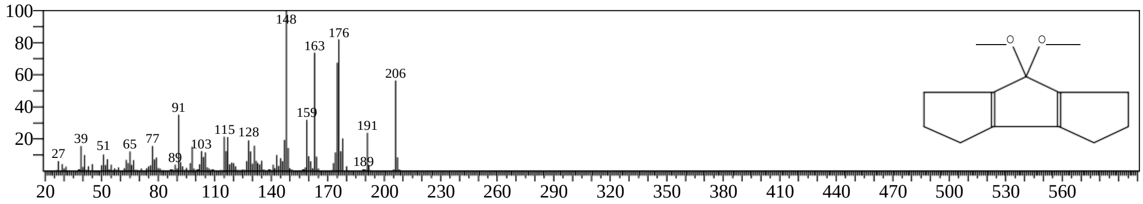

Hit#:2 Entry:23994 Library:NIST20R.lib
SI:63 Formula:C12H14O3 CAS:61227-52-9 MolWeight:206 RetIndex:1657
CompName:4,7-Dimethoxy-2-methylindan-1-one $$ 4,7-Dimethoxy-2-methyl-1-indanone #

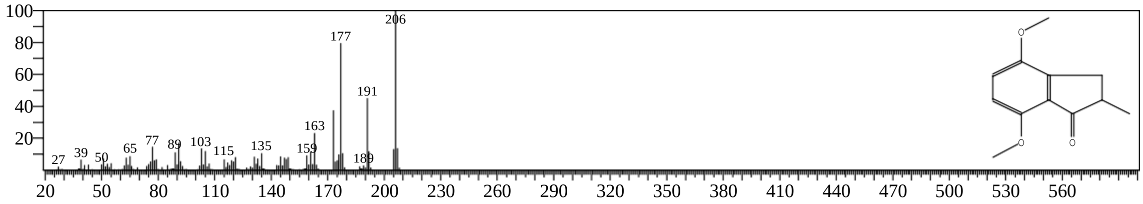

Hit#:3 Entry:58715 Library:NIST20M1.lib
SI:63 Formula:C12H14O3 CAS:61227-52-9 MolWeight:206 RetIndex:1657
CompName:4,7-Dimethoxy-2-methylindan-1-one $$ 4,7-Dimethoxy-2-methyl-1-indanone #

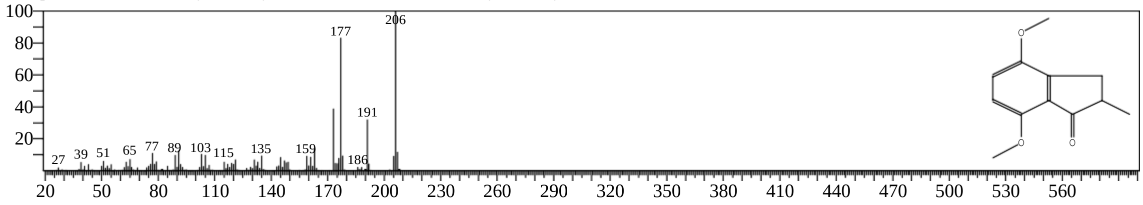

Hit#:4 Entry:59676 Library:NIST20M1.lib
SI:60 Formula:C11H13NO3 CAS:0-00-0 MolWeight:207 RetIndex:1801
CompName:(1E,2Z)-3-(4-hydroxy-3-methoxyphenyl)acrylaldehyde O-methyl oxime

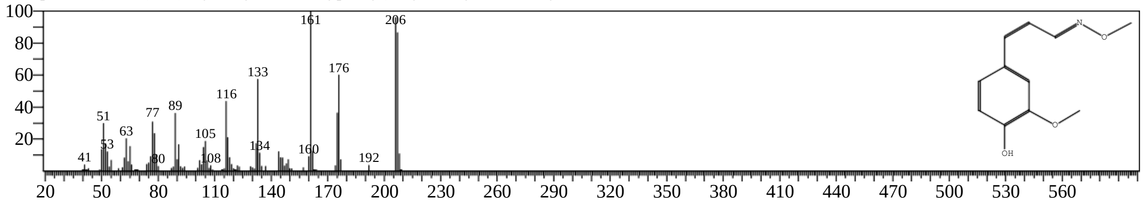

Hit#:5 Entry:58570 Library:NIST20M1.lib
SI:60 Formula:C11H14N2O2 CAS:0-00-0 MolWeight:206 RetIndex:1827
CompName:3-pyrazolidinone, 4-(hydroxymethyl)-4-methyl-1-phenyl- $$ 4-(hydroxymethyl)-4-methyl-1-phenylpyrazolidin-3-one

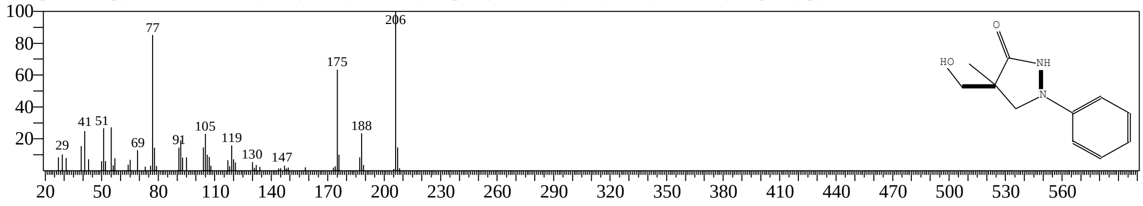

<< Target >>
Line#:18 R.Time:22.667(Scan#:1211) MassPeaks:406
RawMode:Averaged 22.650-22.683(1210-1212) BasePeak:198.00(217338)
BG Mode:Calc. from Peak Group 1 - Event 1 Scan

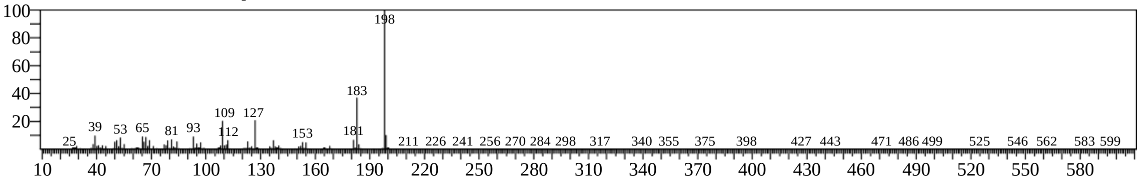

Hit#:1 Entry:22265 Library:NIST20R.lib
SI:96 Formula:C9H10O5 CAS:530-57-4 MolWeight:198 RetIndex:1749
CompName:Benzoic acid, 4-hydroxy-3,5-dimethoxy- $$ Syringic acid $$ 3,5-Dimethoxy-4-hydroxybenzoic acid $$ 4-Hydroxy-3,5-dimethoxybenzoic acid

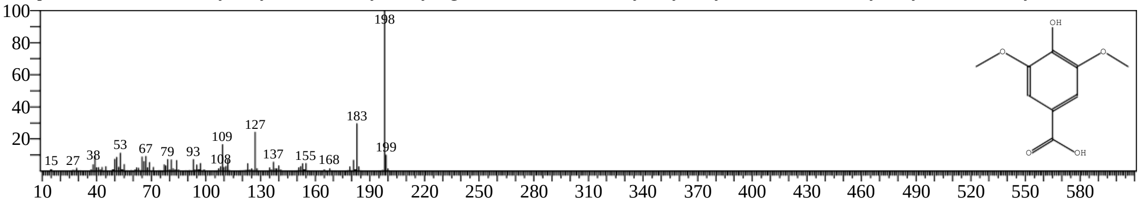

Hit#:2 Entry:22264 Library:NIST20R.lib
SI:95 Formula:C9H10O5 CAS:530-57-4 MolWeight:198 RetIndex:1749
CompName:Benzoic acid, 4-hydroxy-3,5-dimethoxy- $$ Syringic acid $$ 3,5-Dimethoxy-4-hydroxybenzoic acid $$ 4-Hydroxy-3,5-dimethoxybenzoic acid

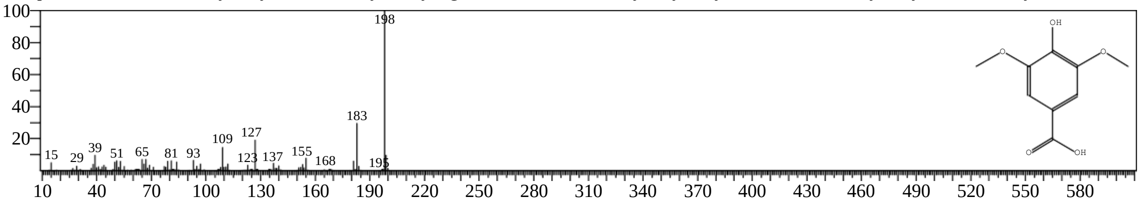

Hit#:3 Entry:22266 Library:NIST20R.lib
SI:94 Formula:C9H10O5 CAS:530-57-4 MolWeight:198 RetIndex:1749
CompName:Benzoic acid, 4-hydroxy-3,5-dimethoxy- $$ Syringic acid $$ 3,5-Dimethoxy-4-hydroxybenzoic acid $$ 4-Hydroxy-3,5-dimethoxybenzoic acid

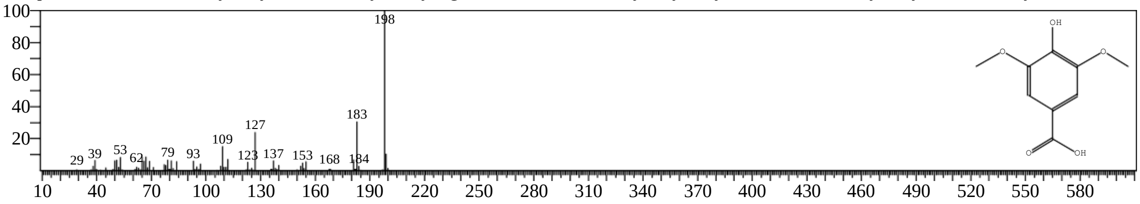

Hit#:4 Entry:51196 Library:NIST20M1.lib
SI:94 Formula:C9H10O5 CAS:530-57-4 MolWeight:198 RetIndex:1749
CompName:Benzoic acid, 4-hydroxy-3,5-dimethoxy- $$ Syringic acid $$ 3,5-Dimethoxy-4-hydroxybenzoic acid $$ 4-Hydroxy-3,5-dimethoxybenzoic acid

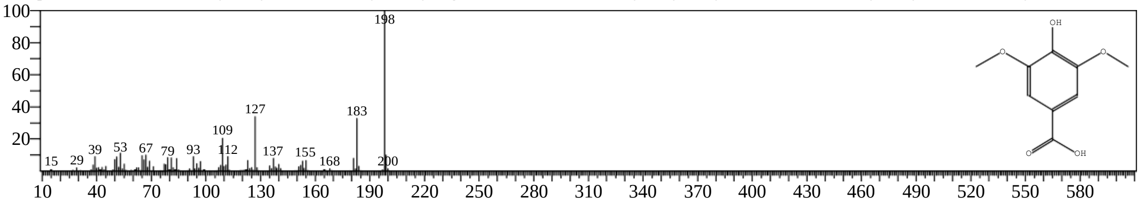

Hit#:5 Entry:22263 Library:NIST20R.lib
SI:89 Formula:C9H10O5 CAS:530-57-4 MolWeight:198 RetIndex:1749
CompName:Benzoic acid, 4-hydroxy-3,5-dimethoxy- $$ Syringic acid $$ 3,5-Dimethoxy-4-hydroxybenzoic acid $$ 4-Hydroxy-3,5-dimethoxybenzoic acid

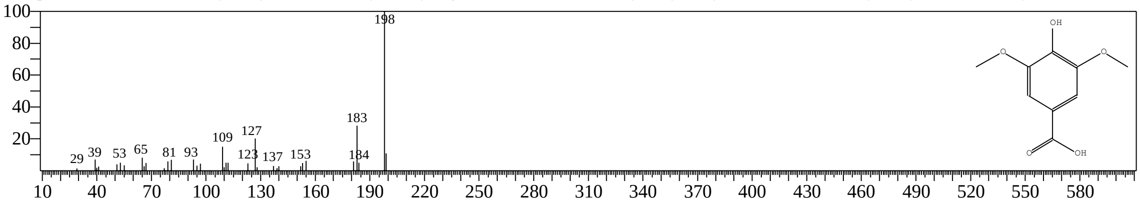

<< Target >>
Line#:19 R.Time:24.667(Scan#:1331) MassPeaks:432
RawMode:Averaged 24.650-24.683(1330-1332) BasePeak:73.05(302431)
BG Mode:Calc. from Peak Group 1 - Event 1 Scan

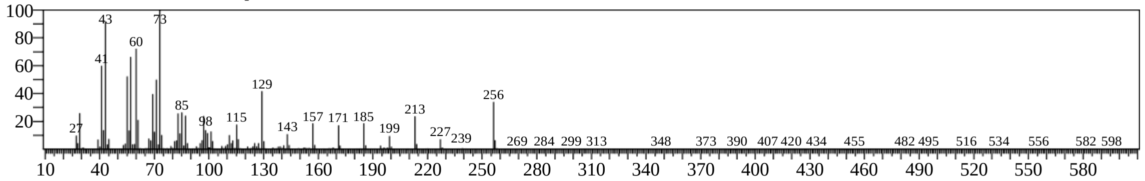

Hit#:1 Entry:31600 Library:NIST20R.lib
SI:95 Formula:C16H32O2 CAS:57-10-3 MolWeight:256 RetIndex:1968
CompName:n-Hexadecanoic acid $$ Hexadecanoic acid $$ n-Hexadecoic acid $$ Palmitic acid $$ Pentadecanecarboxylic acid $$ 1-Pentadecanecarboxylic

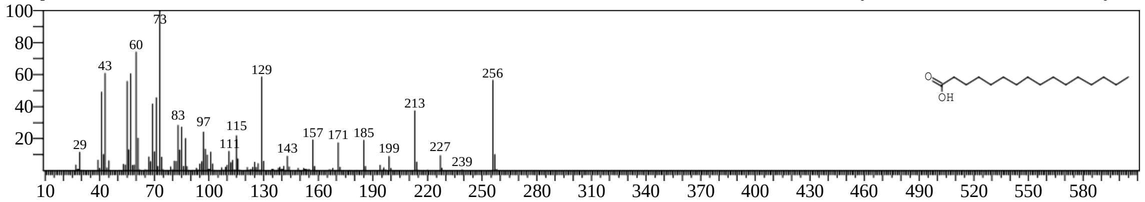

Hit#:2 Entry:31598 Library:NIST20R.lib
SI:92 Formula:C16H32O2 CAS:57-10-3 MolWeight:256 RetIndex:1968
CompName:n-Hexadecanoic acid $$ Hexadecanoic acid $$ n-Hexadecoic acid $$ Palmitic acid $$ Pentadecanecarboxylic acid $$ 1-Pentadecanecarboxylic

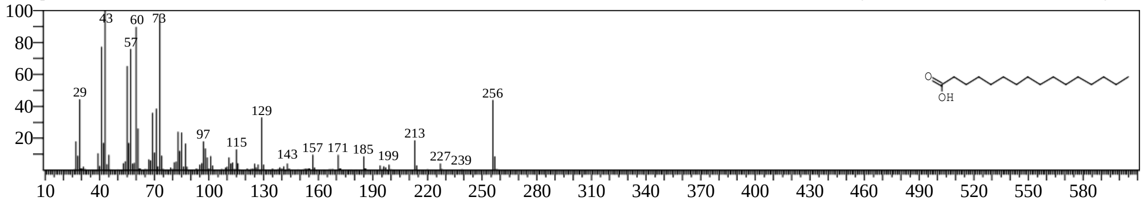

Hit#:3 Entry:44286 Library:NIST20M2.lib
SI:92 Formula:C38H68O8 CAS:28474-90-0 MolWeight:652 RetIndex:4765
CompName:l-(+)-Ascorbic acid 2,6-dihexadecanoate

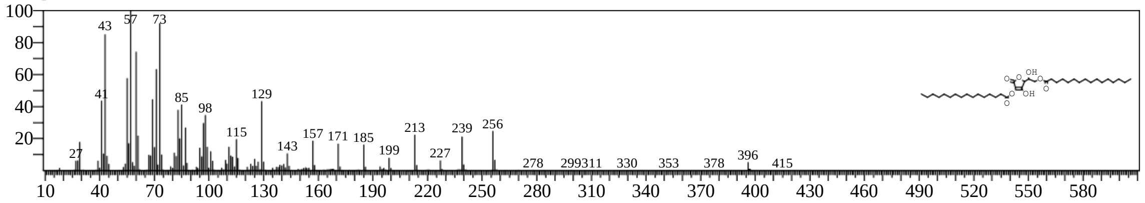

Hit#:4 Entry:31599 Library:NIST20R.lib
SI:92 Formula:C16H32O2 CAS:57-10-3 MolWeight:256 RetIndex:1968
CompName:n-Hexadecanoic acid $$ Hexadecanoic acid $$ n-Hexadecoic acid $$ Palmitic acid $$ Pentadecanecarboxylic acid $$ 1-Pentadecanecarboxylic

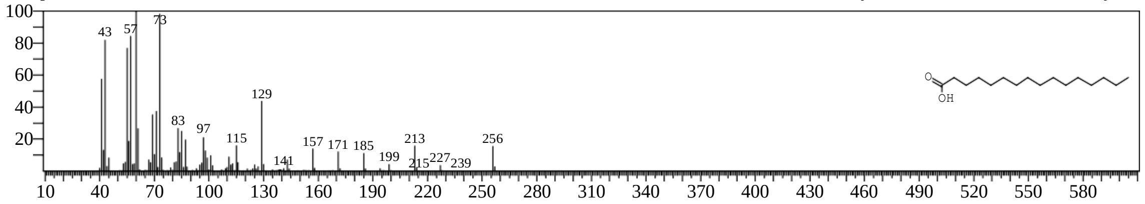

Hit#:5 Entry:96603 Library:NIST20M1.lib
SI:91 Formula:C15H30O2 CAS:1002-84-2 MolWeight:242 RetIndex:1869
CompName:Pentadecanoic acid $$ Pentadecylic acid $$ n-Pentadecanoic acid $$ n-Pentadecylic acid

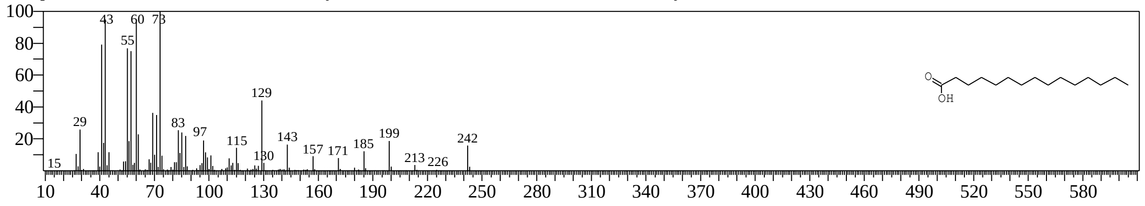

<< Target >>
Line#:20 R.Time:24.967(Scan#:1349) MassPeaks:403
RawMode:Averaged 24.950-24.983(1348-1350) BasePeak:97.15(84912)
BG Mode:Calc. from Peak Group 1 - Event 1 Scan

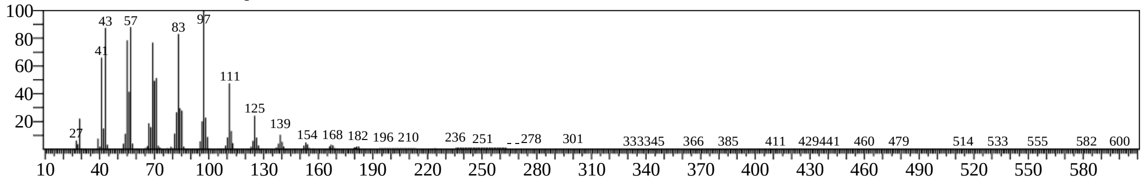

Hit#:1 Entry:32651 Library:NIST20R.lib
SI:97 Formula:C19H38 CAS:18435-45-5 MolWeight:266 RetIndex:1900
CompName:1-Nonadecene $$ Nonadec-1-ene

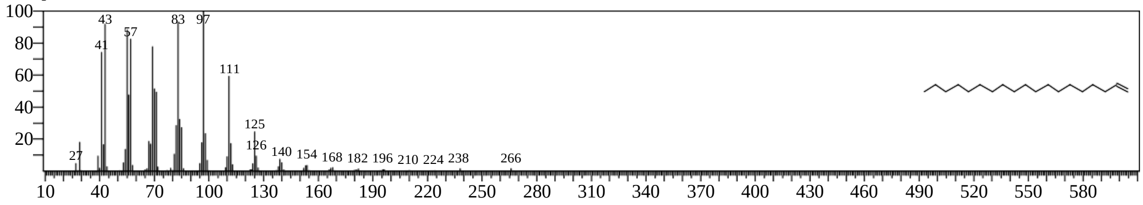

Hit#:2 Entry:40183 Library:NIST20R.lib
SI:96 Formula:C26H52 CAS:18835-33-1 MolWeight:364 RetIndex:2596
CompName:1-Hexacosene

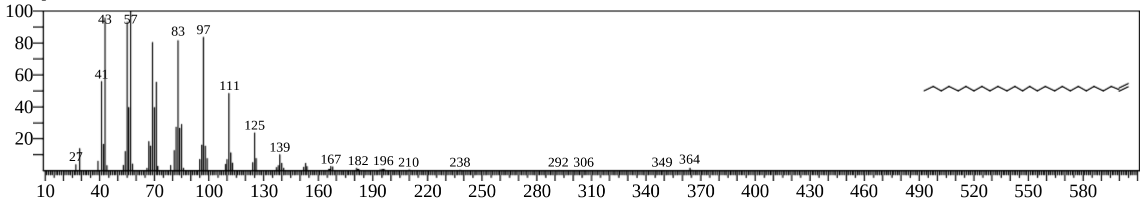

Hit#:3 Entry:38649 Library:NIST20R.lib
SI:96 Formula:C24H48 CAS:10192-32-2 MolWeight:336 RetIndex:2397
CompName:1-Tetracosene

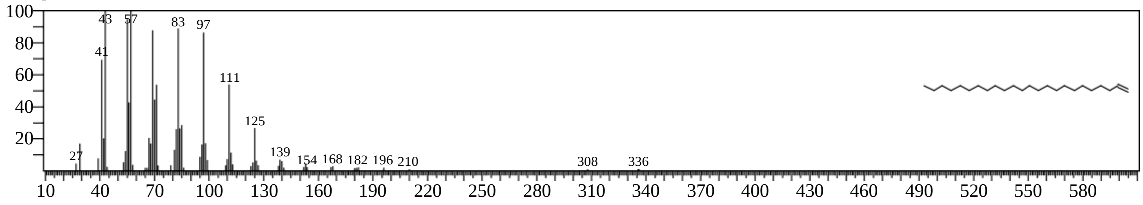

Hit#:4 Entry:37965 Library:NIST20R.lib
SI:96 Formula:C22H46O CAS:661-19-8 MolWeight:326 RetIndex:2451
CompName:Behenic alcohol $$ 1-Docosanol $$ Docosyl alcohol $$ Docosanol-(1) $$ Cachalot BE-22 $$ Loxiol VPG 1451 $$ Docosan-1-ol $$ Behenyl a

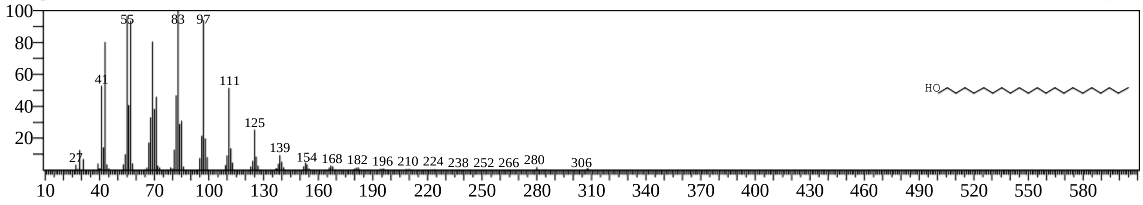

Hit#:5 Entry:178229 Library:NIST20M1.lib
SI:95 Formula:C21H44O CAS:15594-90-8 MolWeight:312 RetIndex:2351
CompName:1-Heneicosanol $$ Henicosan-1-ol $$ Heneicosyl alcohol

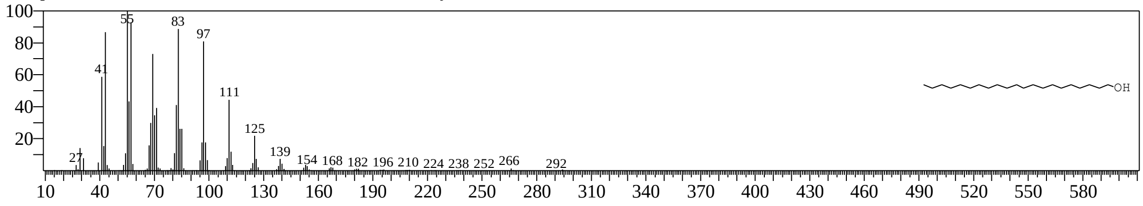

<< Target >>
Line#:21 R.Time:26.967(Scan#:1469) MassPeaks:413
RawMode:Averaged 26.950-26.983(1468-1470) BasePeak:55.05(100651)
BG Mode:Calc. from Peak Group 1 - Event 1 Scan

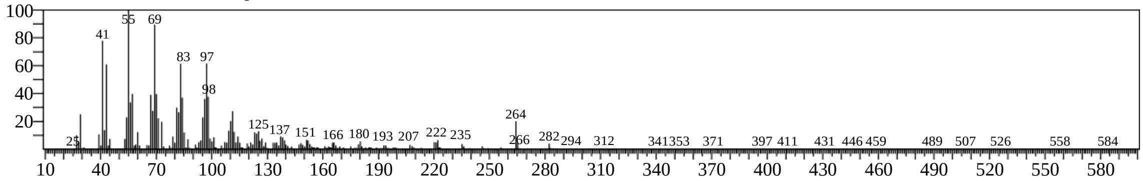

Hit#:1 Entry:34244 Library:NIST20R.lib
SI:95 Formula:C18H34O2 CAS:112-80-1 MolWeight:282 RetIndex:2175
CompName:Oleic Acid $$ 9-Octadecenoic acid (Z)- $$ .DELTA.9-cis-Oleic acid $$ cis-Oleic Acid $$ cis-9-Octadecenoic Acid $$ Emersol 211 $$ Emersol

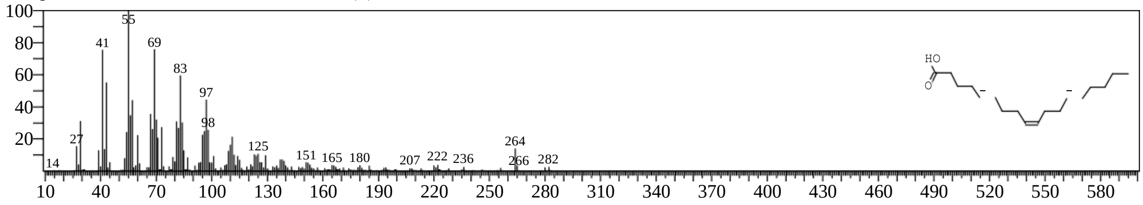

Hit#:2 Entry:34243 Library:NIST20R.lib
SI:94 Formula:C18H34O2 CAS:112-79-8 MolWeight:282 RetIndex:2175
CompName:9-Octadecenoic acid, (E)- $$ trans-.delta.(sup 9)-Octadecenoic acid $$ trans-.delta.9-Octadecenoic acid $$ trans-Octadec-9-enoic acid $$ trans-

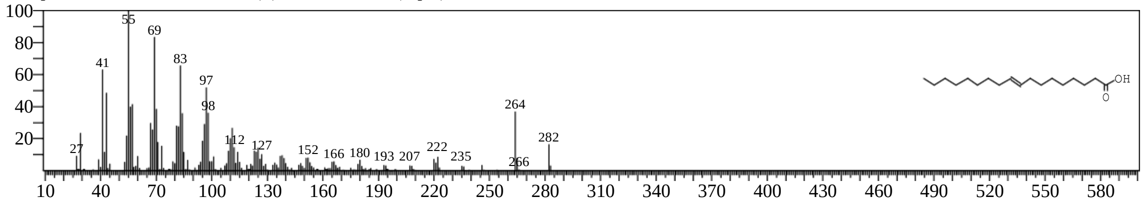

Hit#:3 Entry:142103 Library:NIST20M1.lib
SI:93 Formula:C18H34O2 CAS:593-39-5 MolWeight:282 RetIndex:2175
CompName:6-Octadecenoic acid, (Z)- $$ (6Z)-6-Octadecenoic acid # $$ cis- -6-Octadecenoic acid $$ (Z)-Octadec-6-enoic acid $$ Petroselinic acid

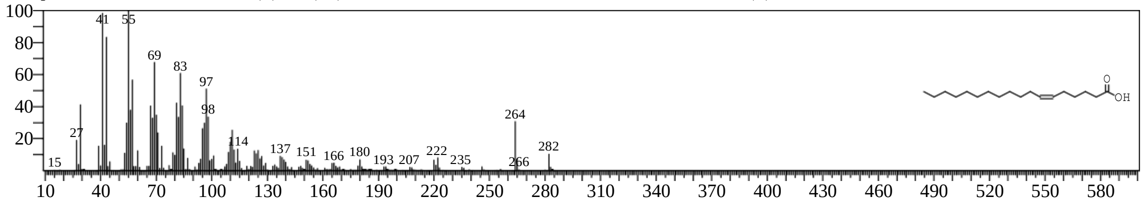

Hit#:4 Entry:142173 Library:NIST20M1.lib
SI:93 Formula:C18H34O2 CAS:112-79-8 MolWeight:282 RetIndex:2175
CompName:9-Octadecenoic acid, (E)- $$ trans-.delta.(sup 9)-Octadecenoic acid $$ trans-.delta.9-Octadecenoic acid $$ trans-Octadec-9-enoic acid $$ trans-

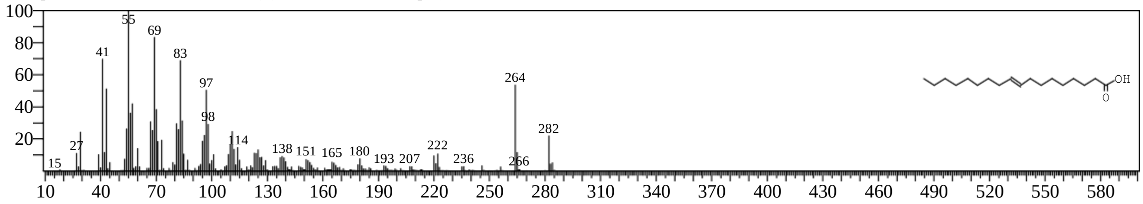

Hit#:5 Entry:142172 Library:NIST20M1.lib
SI:93 Formula:C18H34O2 CAS:2027-47-6 MolWeight:282 RetIndex:2175
CompName:9-Octadecenoic acid

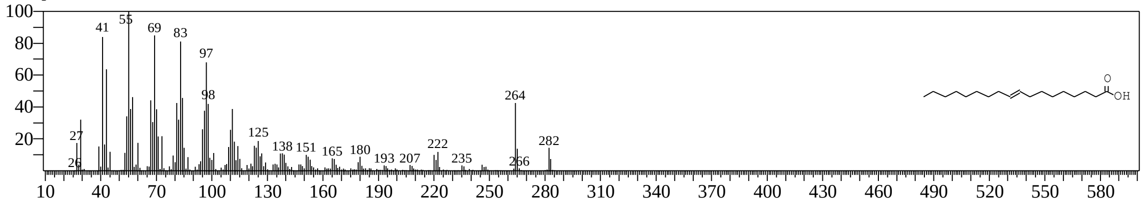

<< Target >>
Line#:22 R.Time:27.167(Scan#:1481) MassPeaks:334
RawMode:Averaged 27.150-27.183(1480-1482) BasePeak:234.05(28505)
BG Mode:Calc. from Peak Group 1 - Event 1 Scan

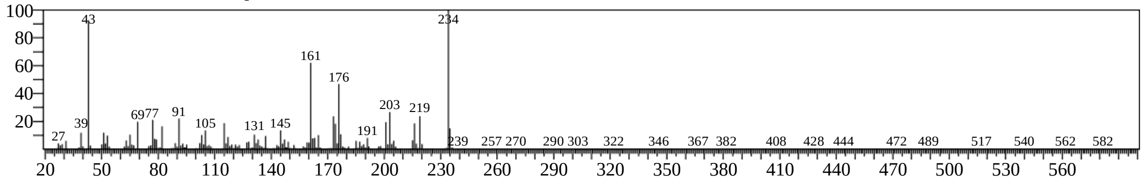

Hit#:1 Entry:87407 Library:NIST20M1.lib
SI:65 Formula:C13H14O4 CAS:1396554-24-7 MolWeight:234 RetIndex:2208
CompName:7-Acetyl-3,6-dihydroxy-8-methyl-3,4-dihydro-2H-naphthalen-1-one $$ 1(2H)-Naphthalenone, 7-acetyl-3,4-dihydro-3,6-dihydroxy-8-methyl-

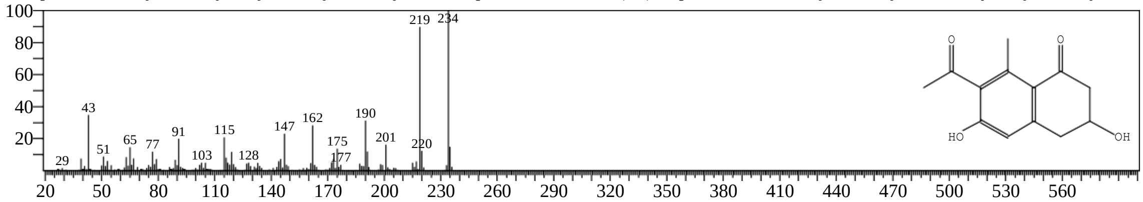

Hit#:2 Entry:87382 Library:NIST20M1.lib
SI:60 Formula:C13H14O4 CAS:349496-09-9 MolWeight:234 RetIndex:1711
CompName:2,4-Pentadienoic acid, 2-acetyl-4-methyl-5-(2-furyl)-, methyl ester $$ Methyl (2E,4E)-2-acetyl-5-(2-furyl)-4-methyl-2,4-pentadienoate #

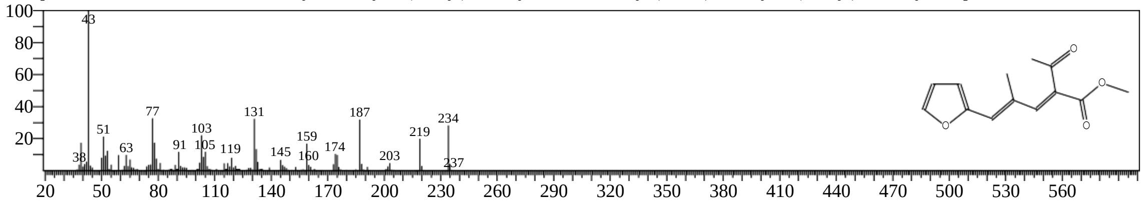

Hit#:3 Entry:87734 Library:NIST20M1.lib
SI:60 Formula:C15H22O2 CAS:469-67-0 MolWeight:234 RetIndex:1866
CompName:Isopetasol $$ 2(3H)-Naphthalenone, 4,4a,5,6,7,8-hexahydro-6-hydroxy-4a,5-dimethyl-3-(1-methylethylidene)-, (4aR,5R,6R)- $$ 2(3H)-Naphth

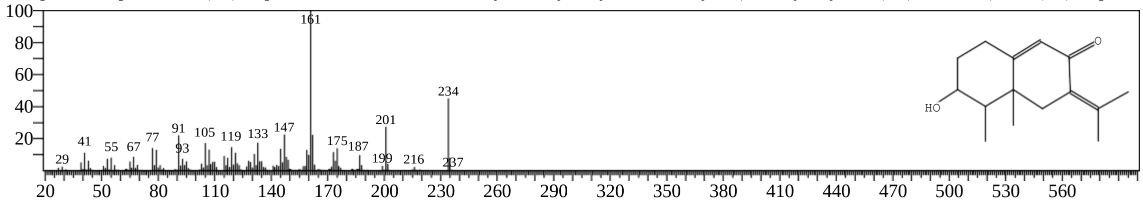

Hit#:4 Entry:33932 Library:NIST20M1.lib
SI:59 Formula:C11H12O2 CAS:5910-25-8 MolWeight:176 RetIndex:1399
CompName:2,4-Pentanedione, 3-phenyl- $$ 3-Phenyl-2,4-pentanedione $$ 3-Phenylacetylacetone

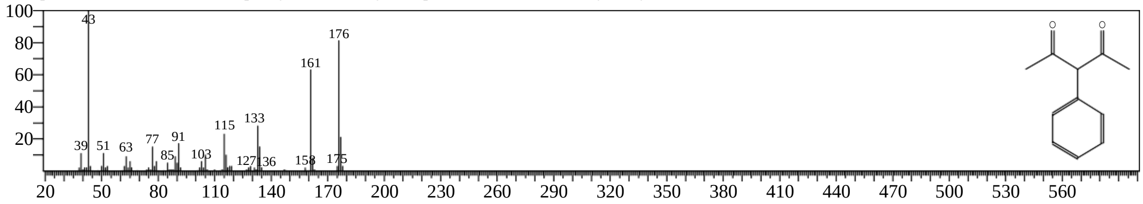

Hit#:5 Entry:87420 Library:NIST20M1.lib
SI:58 Formula:C13H14O4 CAS:21987-07-5 MolWeight:234 RetIndex:2100
CompName:Ethanone, 1-(4,6-dihydroxy-2,3,5-trimethyl-7-benzofuranyl)- $$ Ketone, 4,6-dihydroxy-2,3,5-trimethyl-7-benzofuranyl methyl $$ Usnetol $$ 1-

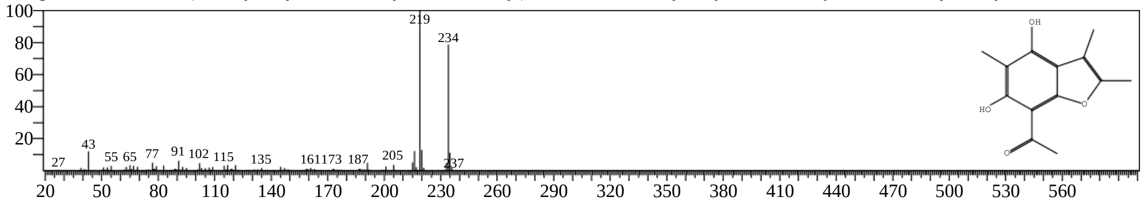

<< Target >>
Line#:23 R.Time:27.283(Scan#:1488) MassPeaks:339
RawMode:Averaged 27.267-27.300(1487-1489) BasePeak:43.10(99968)
BG Mode:Calc. from Peak Group 1 - Event 1 Scan

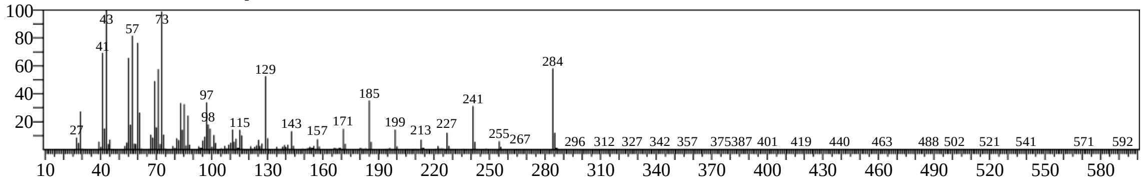

Hit#:1 Entry:34462 Library:NIST20R.lib
SI:94 Formula:C18H36O2 CAS:57-11-4 MolWeight:284 RetIndex:2167
CompName:Octadecanoic acid $$ Stearic acid $$ n-Octadecanoic acid $$ Humko Industrene R $$ Hydrofol Acid 150 $$ Hystrene S-97 $$ Hystrene T-70 $

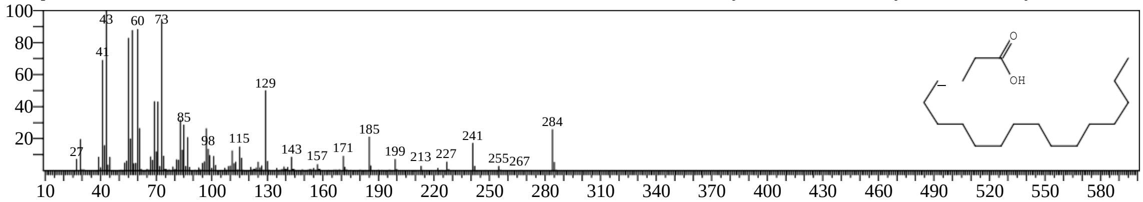

Hit#:2 Entry:144781 Library:NIST20M1.lib
SI:92 Formula:C18H36O2 CAS:57-11-4 MolWeight:284 RetIndex:2167
CompName:Octadecanoic acid $$ Stearic acid $$ n-Octadecanoic acid $$ Humko Industrene R $$ Hydrofol Acid 150 $$ Hystrene S-97 $$ Hystrene T-70 $

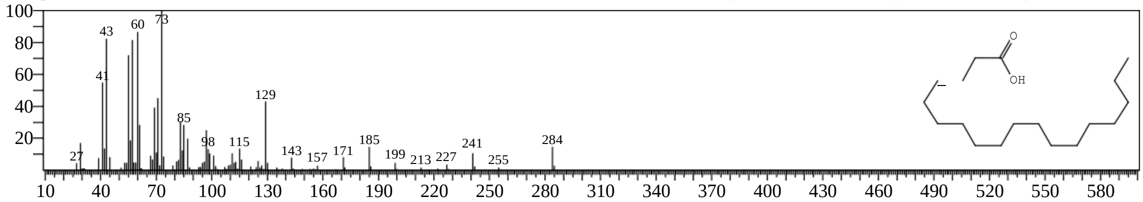

Hit#:3 Entry:34463 Library:NIST20R.lib
SI:91 Formula:C18H36O2 CAS:57-11-4 MolWeight:284 RetIndex:2167
CompName:Octadecanoic acid $$ Stearic acid $$ n-Octadecanoic acid $$ Humko Industrene R $$ Hydrofol Acid 150 $$ Hystrene S-97 $$ Hystrene T-70 $

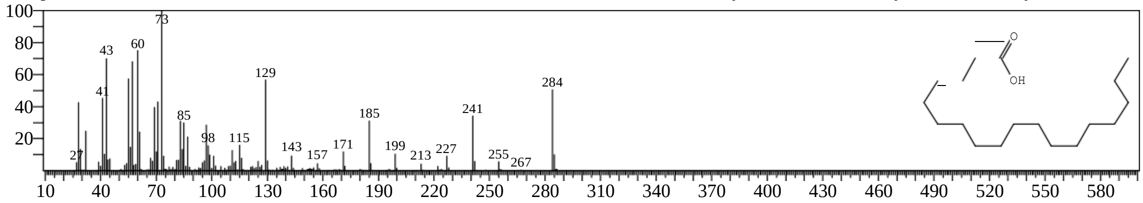

Hit#:4 Entry:34460 Library:NIST20R.lib
SI:91 Formula:C18H36O2 CAS:57-11-4 MolWeight:284 RetIndex:2167
CompName:Octadecanoic acid $$ Stearic acid $$ n-Octadecanoic acid $$ Humko Industrene R $$ Hydrofol Acid 150 $$ Hystrene S-97 $$ Hystrene T-70 $

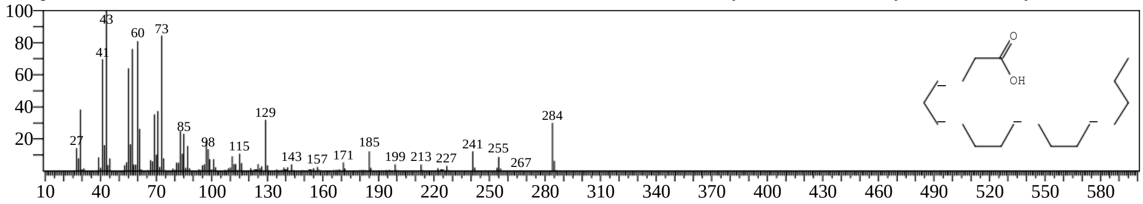

Hit#:5 Entry:96603 Library:NIST20M1.lib
SI:88 Formula:C15H30O2 CAS:1002-84-2 MolWeight:242 RetIndex:1869
CompName:Pentadecanoic acid $$ Pentadecylic acid $$ n-Pentadecanoic acid $$ n-Pentadecylic acid

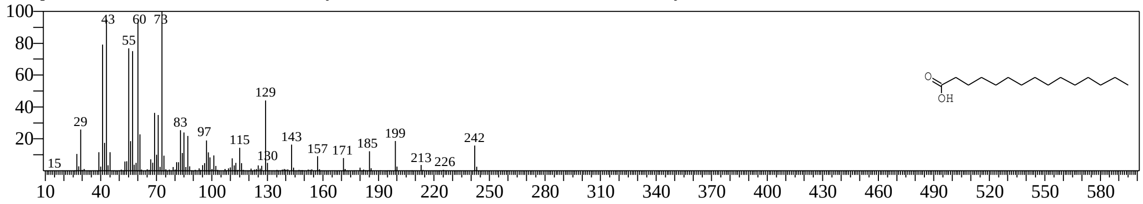

<< Target >>
Line#:24 R.Time:27.633(Scan#:1509) MassPeaks:291
RawMode:Averaged 27.617-27.650(1508-1510) BasePeak:97.15(49524)
BG Mode:Calc. from Peak Group 1 - Event 1 Scan

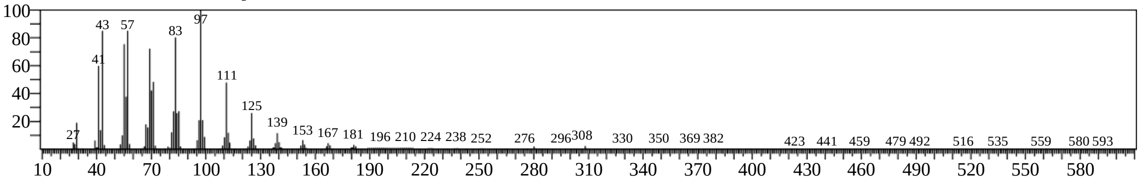

Hit#:1 Entry:32651 Library:NIST20R.lib
SI:96 Formula:C19H38 CAS:18435-45-5 MolWeight:266 RetIndex:1900
CompName:1-Nonadecene $$ Nonadec-1-ene

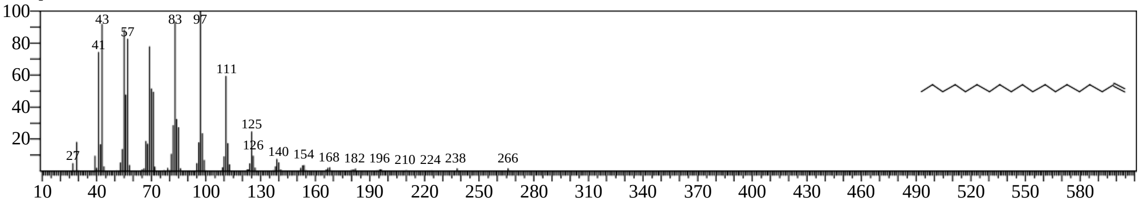

Hit#:2 Entry:40183 Library:NIST20R.lib
SI:96 Formula:C26H52 CAS:18835-33-1 MolWeight:364 RetIndex:2596
CompName:1-Hexacosene

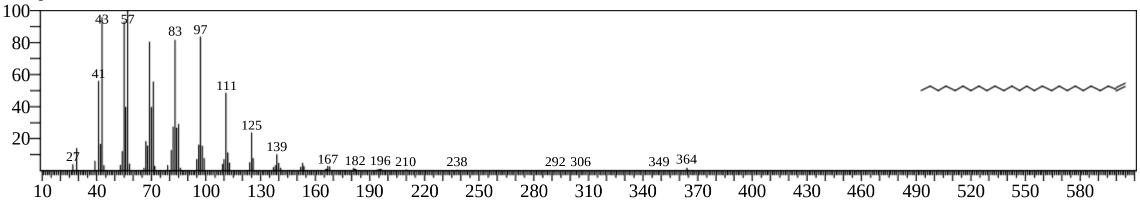

Hit#:3 Entry:38649 Library:NIST20R.lib
SI:96 Formula:C24H48 CAS:10192-32-2 MolWeight:336 RetIndex:2397
CompName:1-Tetracosene

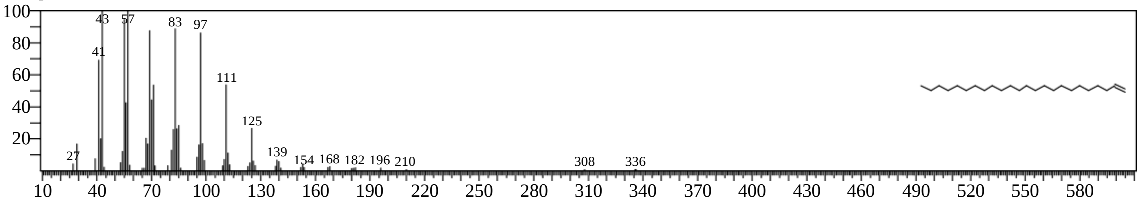

Hit#:4 Entry:37965 Library:NIST20R.lib
SI:96 Formula:C22H46O CAS:661-19-8 MolWeight:326 RetIndex:2451
CompName:Behenic alcohol $$ 1-Docosanol $$ Docosyl alcohol $$ Docosanol-(1) $$ Cachalot BE-22 $$ Loxiol VPG 1451 $$ Docosan-1-ol $$ Behenyl a

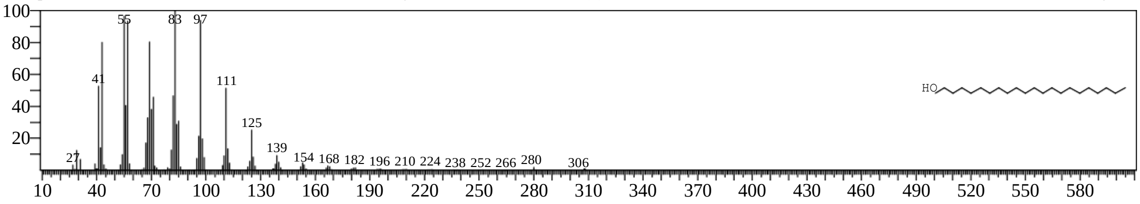

Hit#:5 Entry:224634 Library:NIST20M1.lib
SI:96 Formula:C24H50O CAS:506-51-4 MolWeight:354 RetIndex:2650
CompName:n-Tetracosanol-1 $$ Lignoceric alcohol $$ Lignoceryl alcohol $$ 1-Tetracosanol $$ Tetracosan-1-ol $$ Tetracosyl alcohol $$ Lignocerol $$ Tet

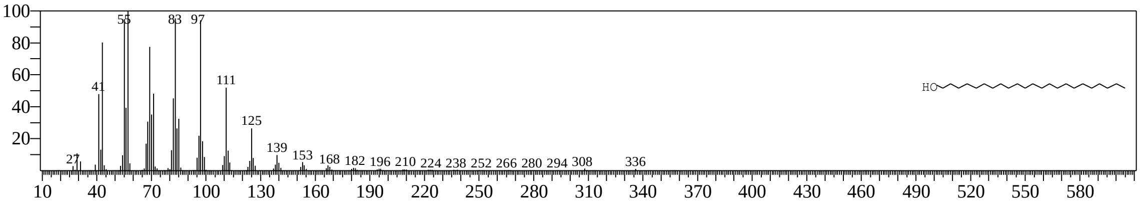

<< Target >>
Line#:25 R.Time:40.750(Scan#:2296) MassPeaks:531
RawMode:Averaged 40.733-40.767(2295-2297) BasePeak:57.10(44856)
BG Mode:Calc. from Peak Group 1 - Event 1 Scan

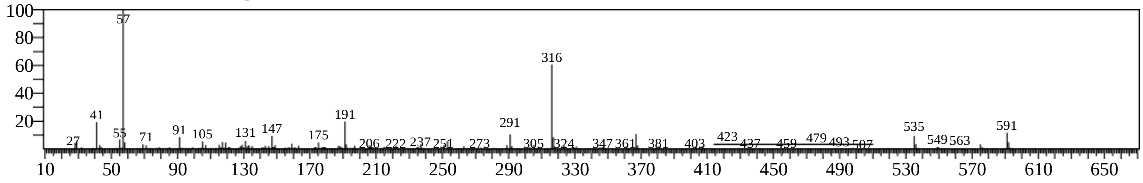

Hit#:1 Entry:44526 Library:NIST20M2.lib
SI:66 Formula:C42H63O4P CAS:95906-11-9 MolWeight:662 RetIndex:0
CompName:Tris(2,4-di-tert-butylphenyl) phosphate $$ Phenol, 2,4-bis(1,1-dimethylethyl)-, 1,1',1''-phosphate $$ Phenol, 2,4-bis(1,1-dimethylethyl)-, phosph

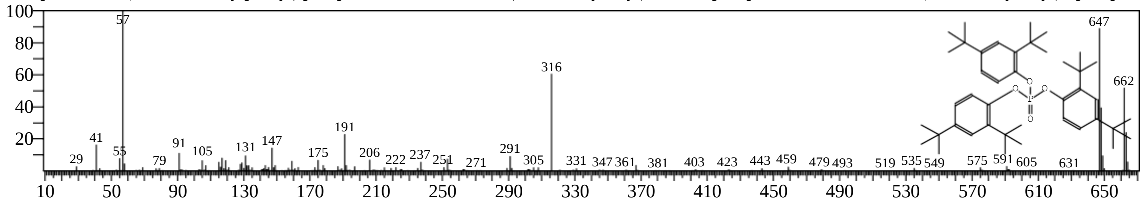

Hit#:2 Entry:214636 Library:NIST20M1.lib
SI:60 Formula:C23H36O2 CAS:56771-86-9 MolWeight:344 RetIndex:2435
CompName:1,4-Epoxynaphthalene-1(2H)-methanol, 4,5,7-tris(1,1-dimethylethyl)-3,4-dihydro-

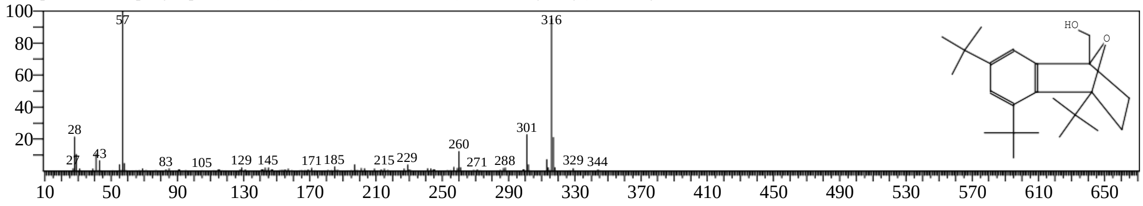

Hit#:3 Entry:182391 Library:NIST20M1.lib
SI:57 Formula:C17H23F3O2 CAS:0-00-0 MolWeight:316 RetIndex:1662
CompName:2,6-Di-tert-butyl-4-methylphenol, O-trifluoroacetyl $$ 2,6-Di-tert-butyl-4-methylphenyl 2,2,2-trifluoroacetate

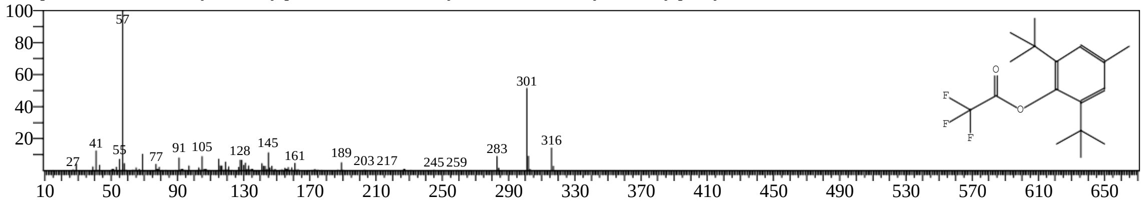

Hit#:4 Entry:42214 Library:NIST20M2.lib
SI:56 Formula:C40H58O3 CAS:134868-71-6 MolWeight:586 RetIndex:4549
CompName:2-tert-Butyl-4,6-bis(3,5-di-tert-butyl-4-hydroxybenzyl)phenol

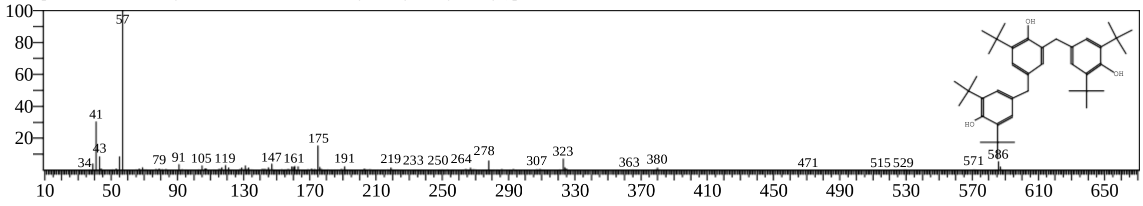

Hit#:5 Entry:43041 Library:NIST20M2.lib
SI:56 Formula:C36H49BrO3 CAS:98007-30-8 MolWeight:608 RetIndex:4542
CompName:4-Bromo-2,6-bis(3,5-di-tert-butyl-4-hydroxybenzyl)phenol

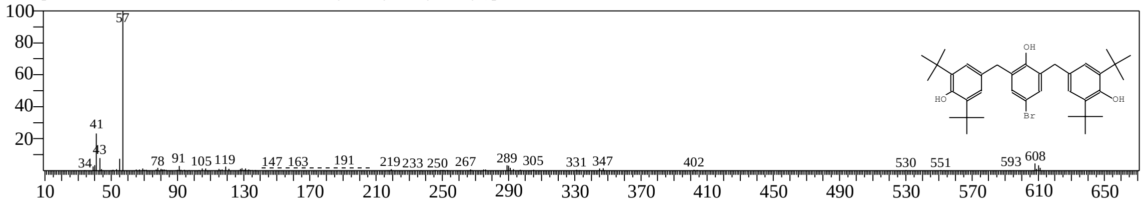

Chromatogram ben_pso_aoc_elu_EAT D:\benher\GCMS BATCH\ben_pso_aoc_elu_EAT.qgd

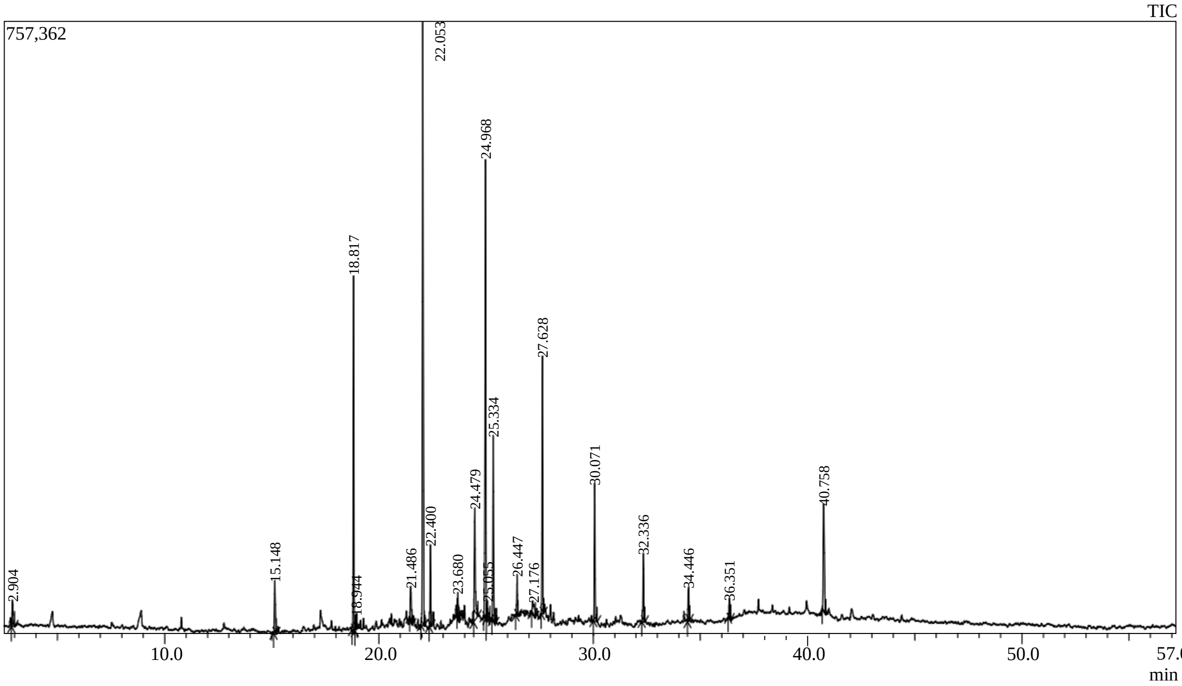

Peak Report TIC

Peak#	R.Time	I.Time	F.Time	Area	Area%	Height	Height%	A/H	Mark	Name
1	2.904	2.850	3.000	105820	1.16	32135	0.98	3.29		1-Butanol, 3-methyl-, acetate
2	15.148	15.083	15.217	169445	1.86	65136	1.99	2.60		Cetene
3	18.817	18.750	18.883	1126642	12.37	437540	13.40	2.57		1-Nonadecene
4	18.944	18.883	18.983	52888	0.58	19074	0.58	2.77		Hexadecane
5	21.486	21.433	21.550	120158	1.32	41324	1.27	2.91		Eicosanoic acid
6	22.053	21.950	22.117	2001824	21.99	747018	22.88	2.68		1-Octadecene
7	22.400	22.350	22.450	260124	2.86	100889	3.09	2.58	V	Octadecanal
8	23.680	23.650	23.750	58205	0.64	28515	0.87	2.04		7,9-Di-tert-butyl-1-oxaspiro(4,5)
9	24.479	24.417	24.617	526721	5.78	138809	4.25	3.79	V	n-Hexadecanoic acid
10	24.968	24.883	25.017	1502977	16.51	565620	17.32	2.66		1-Tetracosene
11	25.055	25.017	25.183	85603	0.94	22872	0.70	3.74	V	Carbonic acid, eicosyl prop-1-en
12	25.334	25.267	25.433	620412	6.81	229668	7.03	2.70		Octadecanal
13	26.447	26.383	26.500	129778	1.43	49725	1.52	2.61		Octadecanenitrile
14	27.176	27.117	27.300	94787	1.04	15643	0.48	6.06		Octadecanoic acid
15	27.628	27.567	27.683	866305	9.51	318024	9.74	2.72		1-Tetracosene
16	30.071	30.000	30.183	488934	5.37	170352	5.22	2.87		1-Hexacosene
17	32.336	32.267	32.417	242627	2.66	84359	2.58	2.88		Octacosanol
18	34.446	34.400	34.500	121041	1.33	42330	1.30	2.86	V	Octadecyl trifluoroacetate
19	36.351	36.300	36.417	63069	0.69	23472	0.72	2.69		Tricosyl trifluoroacetate
20	40.758	40.683	40.850	468013	5.14	132489	4.06	3.53		Tris(2,4-di-tert-butylphenyl) pho
				9105373	100.00	3264994	100.00			

Library

<< Target >>
Line#:1 R.Time:2.900(Scan#:25) MassPeaks:363
RawMode:Averaged 2.883-2.917(24-26) BasePeak:43.05(7496)
BG Mode:Calc. from Peak Group 1 - Event 1 Scan

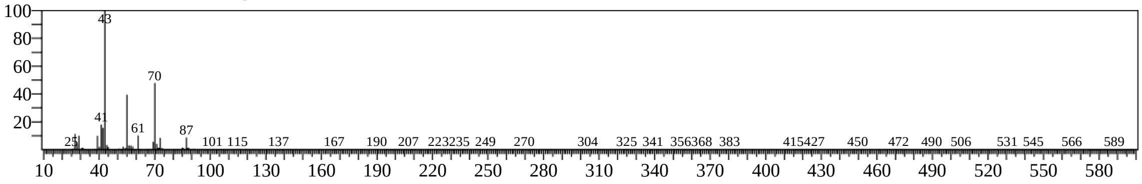

Hit#:1 Entry:6782 Library:NIST20R.lib
SI:96 Formula:C7H14O2 CAS:123-92-2 MolWeight:130 RetIndex:820
CompName:1-Butanol, 3-methyl-, acetate $$ Isopentyl alcohol, acetate $$ Acetic acid, 3-methylbutyl ester $$ Banana oil $$ Isoamyl acetate $$ Isoamyl etha

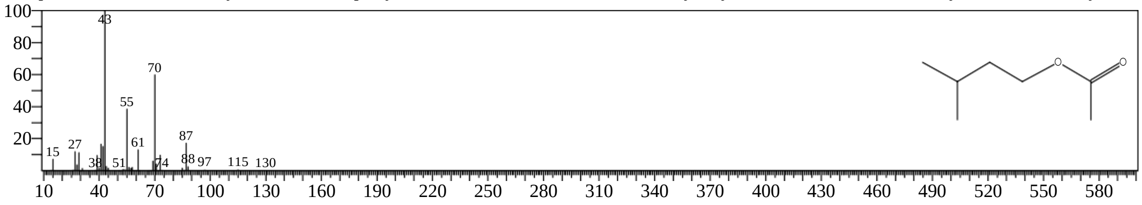

Hit#:2 Entry:6785 Library:NIST20R.lib
SI:95 Formula:C7H14O2 CAS:123-92-2 MolWeight:130 RetIndex:820
CompName:1-Butanol, 3-methyl-, acetate $$ Isopentyl alcohol, acetate $$ Acetic acid, 3-methylbutyl ester $$ Banana oil $$ Isoamyl acetate $$ Isoamyl etha

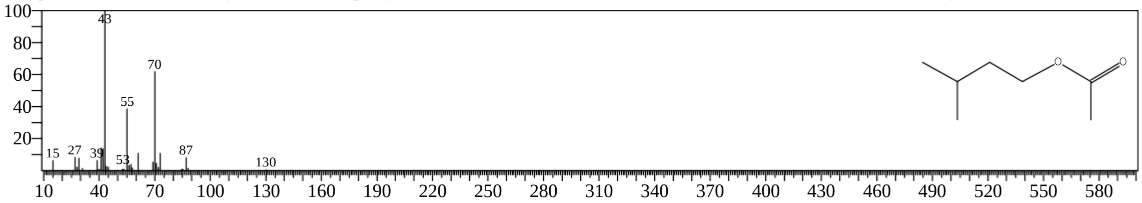

Hit#:3 Entry:8673 Library:NIST20M1.lib
SI:95 Formula:C7H14O2 CAS:123-92-2 MolWeight:130 RetIndex:820
CompName:1-Butanol, 3-methyl-, acetate $$ Isopentyl alcohol, acetate $$ Acetic acid, 3-methylbutyl ester $$ Banana oil $$ Isoamyl acetate $$ Isoamyl etha

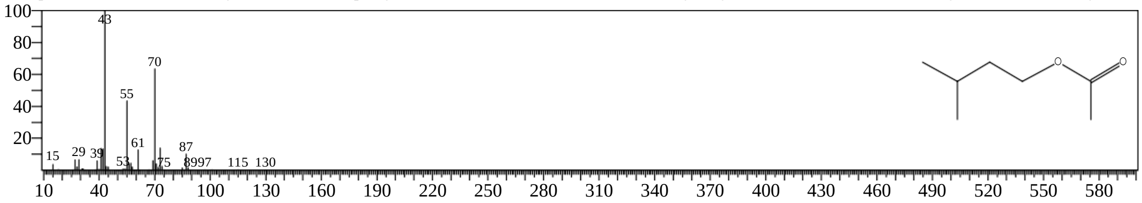

Hit#:4 Entry:6784 Library:NIST20R.lib
SI:95 Formula:C7H14O2 CAS:123-92-2 MolWeight:130 RetIndex:820
CompName:1-Butanol, 3-methyl-, acetate $$ Isopentyl alcohol, acetate $$ Acetic acid, 3-methylbutyl ester $$ Banana oil $$ Isoamyl acetate $$ Isoamyl etha

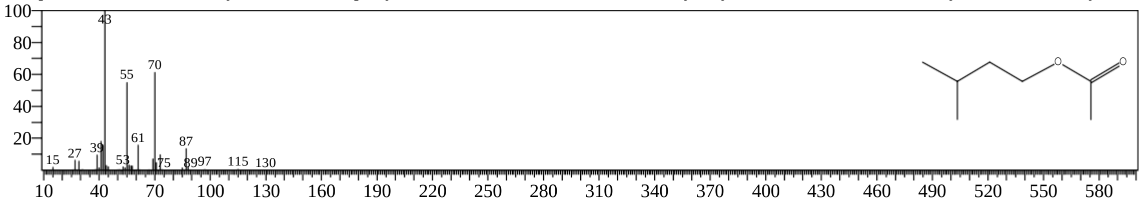

Hit#:5 Entry:6783 Library:NIST20R.lib
SI:93 Formula:C7H14O2 CAS:123-92-2 MolWeight:130 RetIndex:820
CompName:1-Butanol, 3-methyl-, acetate $$ Isopentyl alcohol, acetate $$ Acetic acid, 3-methylbutyl ester $$ Banana oil $$ Isoamyl acetate $$ Isoamyl etha

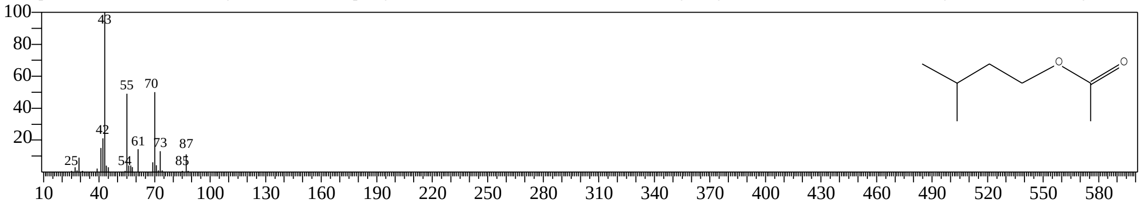

<< Target >>
Line#:2 R.Time:15.150(Scan#:760) MassPeaks:348
RawMode:Averaged 15.133-15.167(759-761) BasePeak:41.05(4835)
BG Mode:Calc. from Peak Group 1 - Event 1 Scan

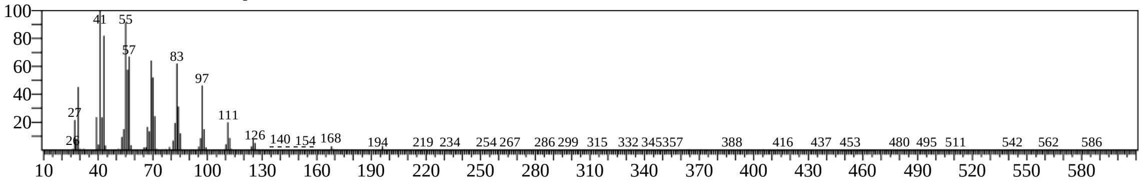

Hit#:1 Entry:27374 Library:NIST20R.lib
SI:97 Formula:C16H32 CAS:629-73-2 MolWeight:224 RetIndex:1602
CompName:Cetene $$ 1-Hexadecene $$ .alpha.-Hexadecene $$ n-Hexadec-1-ene $$ 1-Cetene $$ Hexadecylene-1 $$ Hexadec-1-ene $$ Hexadecene-1 $$ N

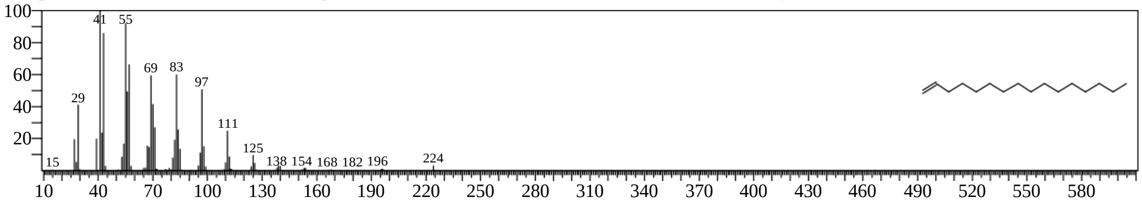

Hit#:2 Entry:22010 Library:NIST20R.lib
SI:97 Formula:C14H28 CAS:1120-36-1 MolWeight:196 RetIndex:1403
CompName:1-Tetradecene $$ n-Tetradec-1-ene $$ .alpha.-Tetradecene $$ Neodene 14 $$ Tetradec-1-ene $$ Tetradecene-1

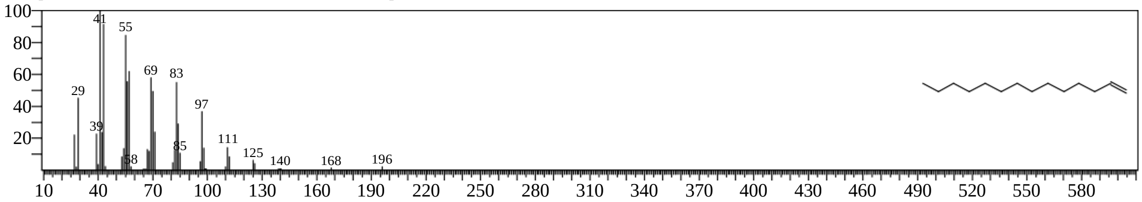

Hit#:3 Entry:24888 Library:NIST20R.lib
SI:97 Formula:C15H30 CAS:13360-61-7 MolWeight:210 RetIndex:1502
CompName:1-Pentadecene $$ Pentadecene,1- $$ Pentadec-1-ene

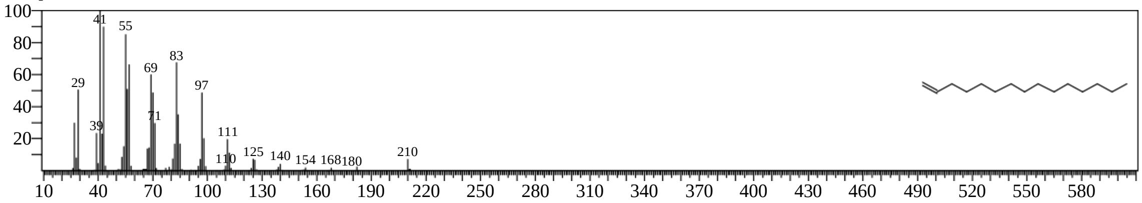

Hit#:4 Entry:27376 Library:NIST20R.lib
SI:97 Formula:C16H32 CAS:629-73-2 MolWeight:224 RetIndex:1602
CompName:Cetene $$ 1-Hexadecene $$ .alpha.-Hexadecene $$ n-Hexadec-1-ene $$ 1-Cetene $$ Hexadecylene-1 $$ Hexadec-1-ene $$ Hexadecene-1 $$ N

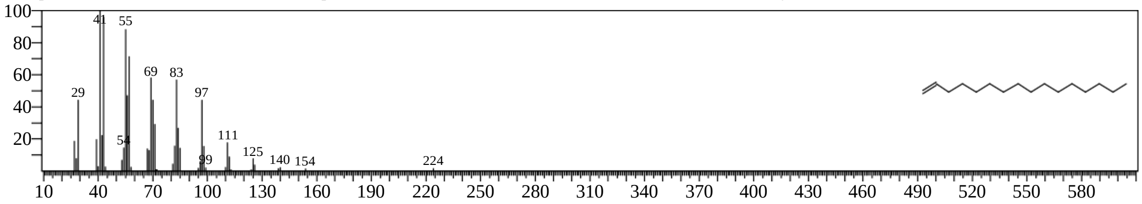

Hit#:5 Entry:27373 Library:NIST20R.lib
SI:96 Formula:C16H32 CAS:629-73-2 MolWeight:224 RetIndex:1602
CompName:Cetene $$ 1-Hexadecene $$ .alpha.-Hexadecene $$ n-Hexadec-1-ene $$ 1-Cetene $$ Hexadecylene-1 $$ Hexadec-1-ene $$ Hexadecene-1 $$ N

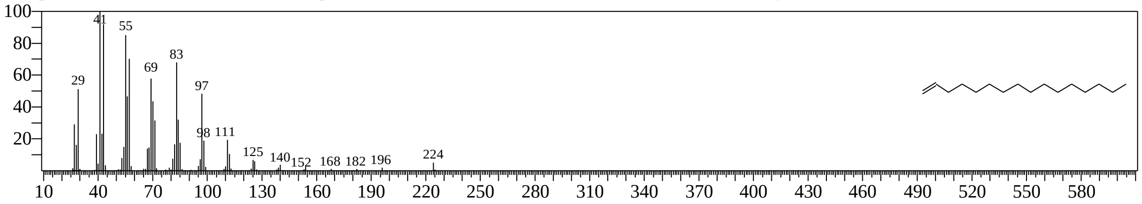

<< Target >>
Line#:3 R.Time:18.817(Scan#:980) MassPeaks:313
RawMode:Averaged 18.800-18.833(979-981) BasePeak:41.05(29222)
BG Mode:Calc. from Peak Group 1 - Event 1 Scan

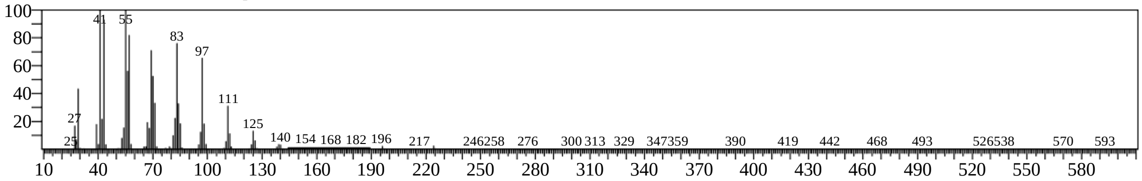

Hit#:1 Entry:32652 Library:NIST20R.lib
SI:97 Formula:C19H38 CAS:18435-45-5 MolWeight:266 RetIndex:1900
CompName:1-Nonadecene $$ Nonadec-1-ene

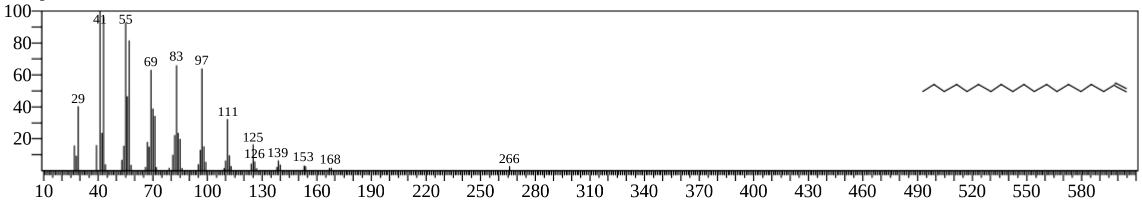

Hit#:2 Entry:107593 Library:NIST20M1.lib
SI:97 Formula:C18H36 CAS:112-88-9 MolWeight:252 RetIndex:1801
CompName:1-Octadecene $$ .alpha.-Octadecene $$ Octadecylene, .alpha.- $$ Neodene 18 $$ Octadec-1-ene $$ Octadecene-1 $$ Octadeca-1-ene

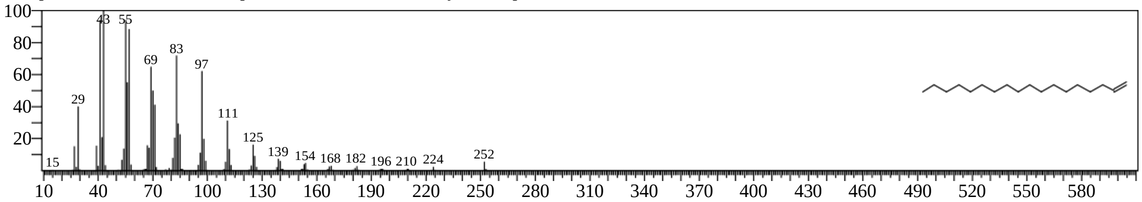

Hit#:3 Entry:27375 Library:NIST20R.lib
SI:96 Formula:C16H32 CAS:629-73-2 MolWeight:224 RetIndex:1602
CompName:Cetene $$ 1-Hexadecene $$ .alpha.-Hexadecene $$ n-Hexadec-1-ene $$ 1-Cetene $$ Hexadecylene-1 $$ Hexadec-1-ene $$ Hexadecene-1 $$ N

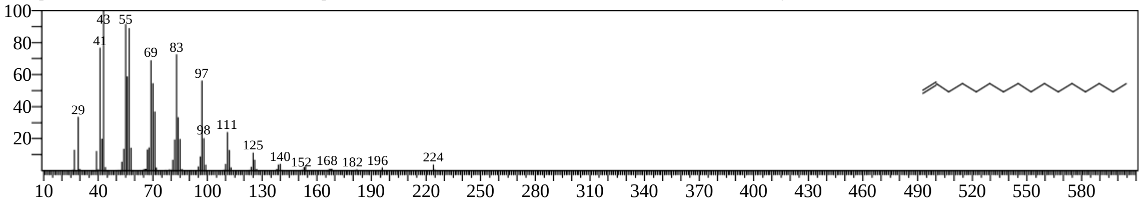

Hit#:4 Entry:31115 Library:NIST20R.lib
SI:96 Formula:C18H36 CAS:112-88-9 MolWeight:252 RetIndex:1801
CompName:1-Octadecene $$ .alpha.-Octadecene $$ Octadecylene, .alpha.- $$ Neodene 18 $$ Octadec-1-ene $$ Octadecene-1 $$ Octadeca-1-ene

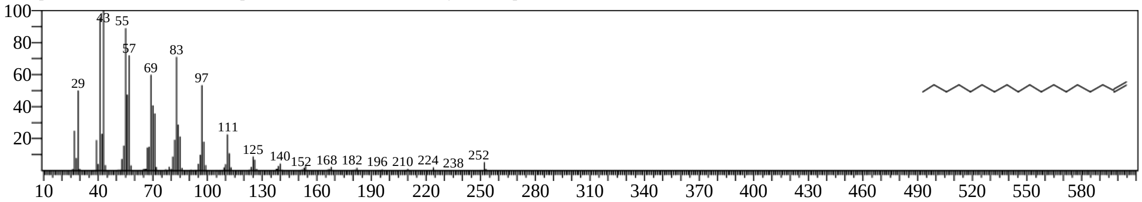

Hit#:5 Entry:107601 Library:NIST20M1.lib
SI:96 Formula:C18H36 CAS:7206-19-1 MolWeight:252 RetIndex:1818
CompName:3-Octadecene, (E)- $$ (3E)-3-Octadecene $$ (E)-3-Octadecene $$ trans-3-Octadecene

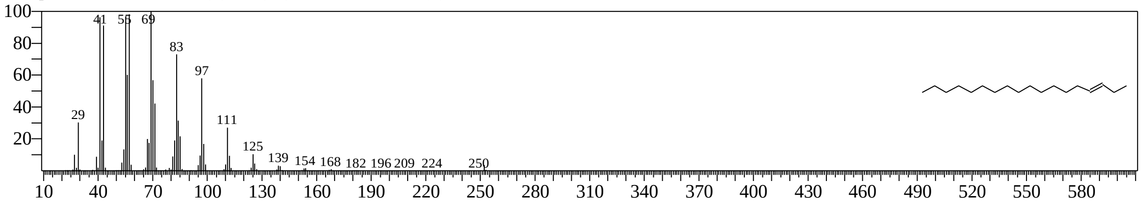

<< Target >>
Line#:4 R.Time:18.950(Scan#:988) MassPeaks:291
RawMode:Averaged 18.933-18.967(987-989) BasePeak:57.10(2744)
BG Mode:Calc. from Peak Group 1 - Event 1 Scan

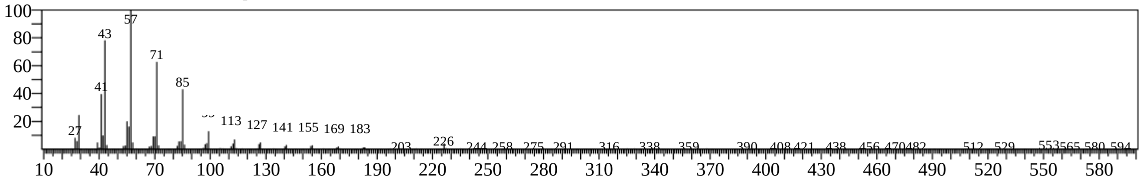

Hit#:1 Entry:27736 Library:NIST20R.lib
SI:96 Formula:C16H34 CAS:544-76-3 MolWeight:226 RetIndex:1600
CompName:Hexadecane $$ n-Cetane $$ n-Hexadecane $$ Cetane

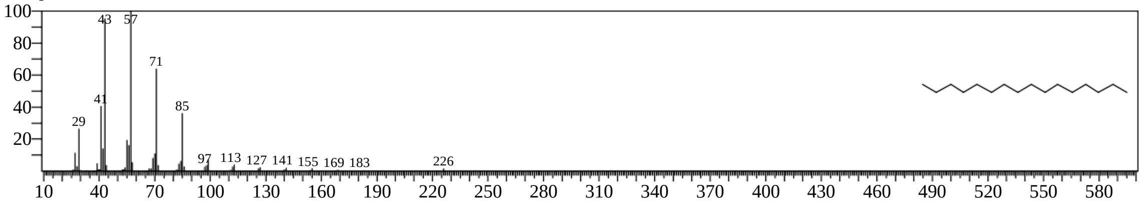

Hit#:2 Entry:22497 Library:NIST20R.lib
SI:96 Formula:C14H30 CAS:629-59-4 MolWeight:198 RetIndex:1400
CompName:Tetradecane $$ n-Tetradecane

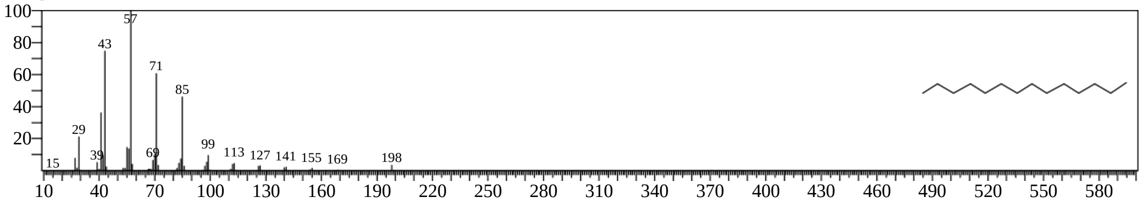

Hit#:3 Entry:25293 Library:NIST20R.lib
SI:96 Formula:C15H32 CAS:629-62-9 MolWeight:212 RetIndex:1500
CompName:Pentadecane $$ n-Pentadecane $$ CH3(CH2)13CH3

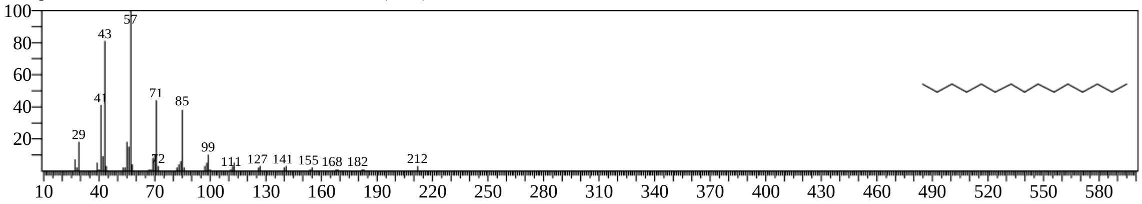

Hit#:4 Entry:32904 Library:NIST20R.lib
SI:96 Formula:C19H40 CAS:629-92-5 MolWeight:268 RetIndex:1900
CompName:Nonadecane $$ n-Nonadecane

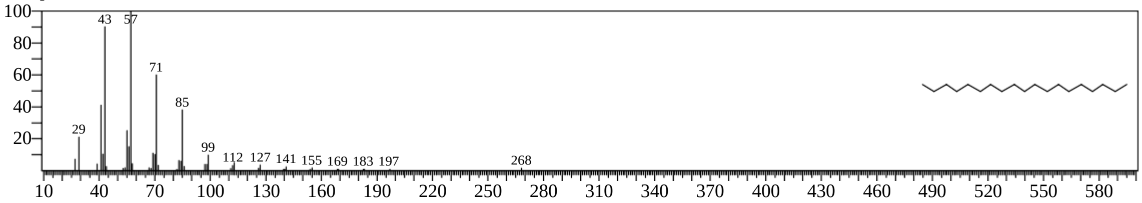

Hit#:5 Entry:25295 Library:NIST20R.lib
SI:96 Formula:C15H32 CAS:629-62-9 MolWeight:212 RetIndex:1500
CompName:Pentadecane $$ n-Pentadecane $$ CH3(CH2)13CH3

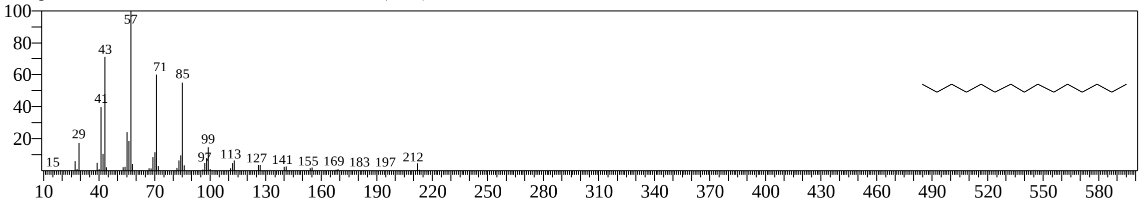

<< Target >>
Line#:5 R.Time:21.483(Scan#:1140) MassPeaks:350
RawMode:Averaged 21.467-21.500(1139-1141) BasePeak:43.10(2755)
BG Mode:Calc. from Peak Group 1 - Event 1 Scan

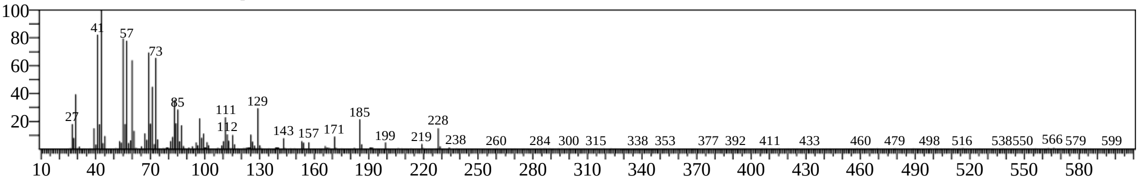

Hit#:1 Entry:36903 Library:NIST20R.lib
SI:90 Formula:C20H40O2 CAS:506-30-9 MolWeight:312 RetIndex:2366
CompName:Eicosanoic acid $$ Arachic acid $$ Arachidic acid $$ Icosanoic acid $$ n-Eicosanoic acid $$ Arachidic acid (synthetic)

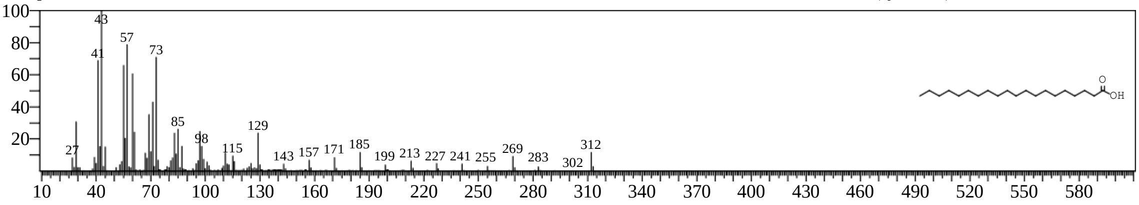

Hit#:2 Entry:96603 Library:NIST20M1.lib
SI:89 Formula:C15H30O2 CAS:1002-84-2 MolWeight:242 RetIndex:1869
CompName:Pentadecanoic acid $$ Pentadecylic acid $$ n-Pentadecanoic acid $$ n-Pentadecylic acid

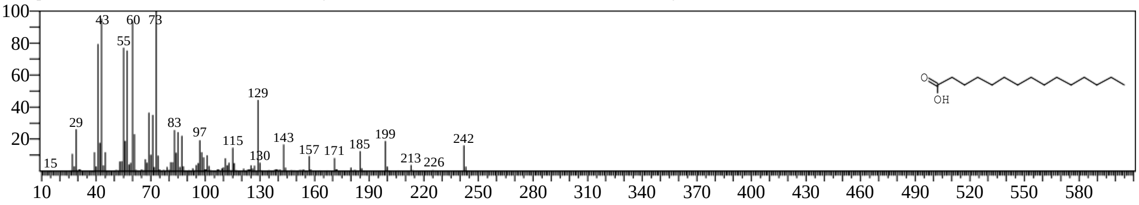

Hit#:3 Entry:28009 Library:NIST20R.lib
SI:89 Formula:C14H28O2 CAS:544-63-8 MolWeight:228 RetIndex:1769
CompName:Tetradecanoic acid $$ Myristic acid $$ n-Tetradecanoic acid $$ n-Tetradecoic acid $$ Neo-Fat 14 $$ Univol U 316S $$ 1-Tridecanecarboxylic

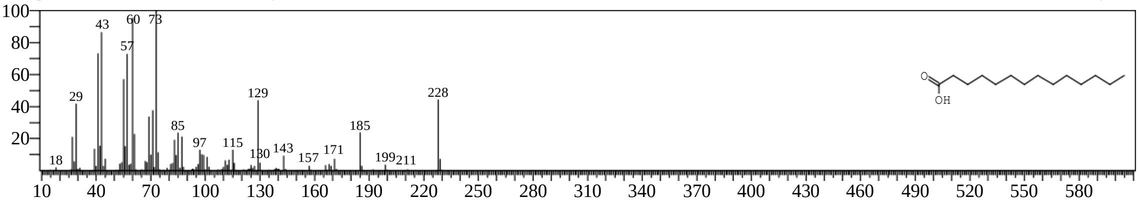

Hit#:4 Entry:81713 Library:NIST20M1.lib
SI:89 Formula:C14H28O2 CAS:544-63-8 MolWeight:228 RetIndex:1769
CompName:Tetradecanoic acid $$ Myristic acid $$ n-Tetradecanoic acid $$ n-Tetradecoic acid $$ Neo-Fat 14 $$ Univol U 316S $$ 1-Tridecanecarboxylic

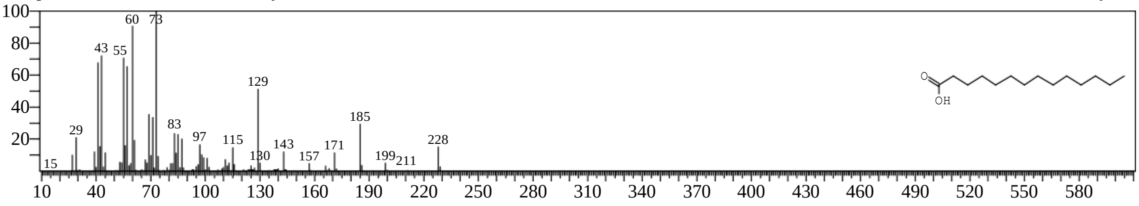

Hit#:5 Entry:34462 Library:NIST20R.lib
SI:89 Formula:C18H36O2 CAS:57-11-4 MolWeight:284 RetIndex:2167
CompName:Octadecanoic acid $$ Stearic acid $$ n-Octadecanoic acid $$ Humko Industrene R $$ Hydrofol Acid 150 $$ Hystrene S-97 $$ Hystrene T-70 $

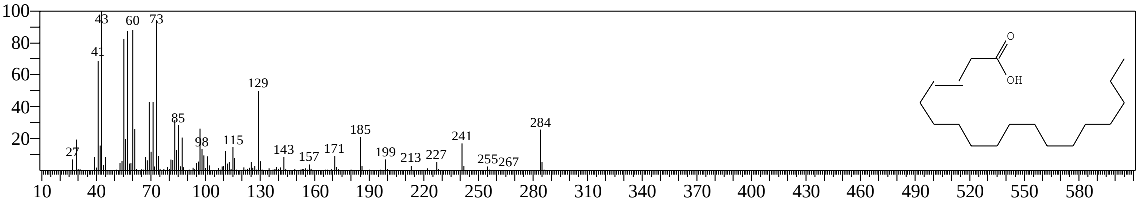

<< Target >>
Line#:6 R.Time:22.050(Scan#:1174) MassPeaks:285
RawMode:Averaged 22.033-22.067(1173-1175) BasePeak:55.05(47646)
BG Mode:Calc. from Peak Group 1 - Event 1 Scan

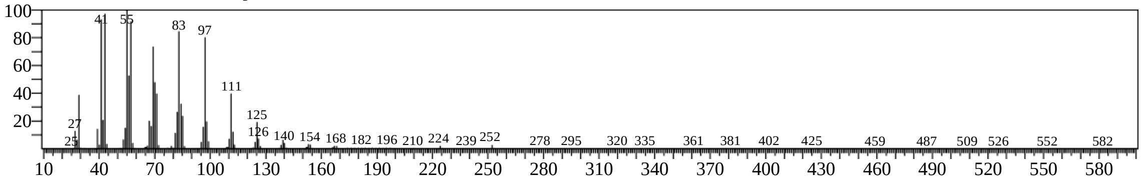

Hit#:1 Entry:107593 Library:NIST20M1.lib
SI:97 Formula:C18H36 CAS:112-88-9 MolWeight:252 RetIndex:1801
CompName:1-Octadecene $$ .alpha.-Octadecene $$ Octadecylene, .alpha.- $$ Neodene 18 $$ Octadec-1-ene $$ Octadecene-1 $$ Octadeca-1-ene

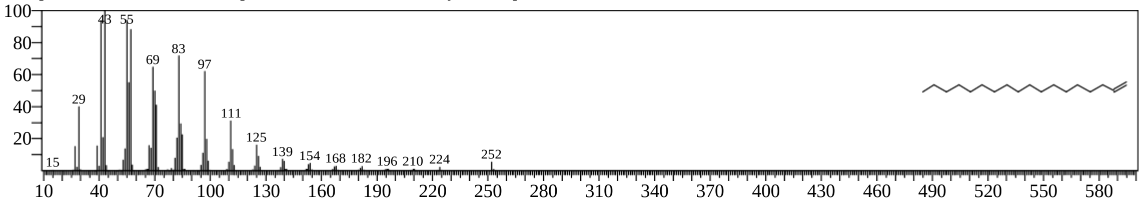

Hit#:2 Entry:32652 Library:NIST20R.lib
SI:96 Formula:C19H38 CAS:18435-45-5 MolWeight:266 RetIndex:1900
CompName:1-Nonadecene $$ Nonadec-1-ene

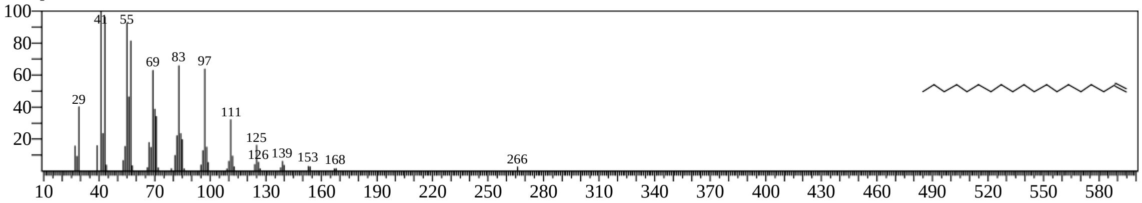

Hit#:3 Entry:156666 Library:NIST20M1.lib
SI:96 Formula:C21H42 CAS:95008-11-0 MolWeight:294 RetIndex:2117
CompName:10-Heneicosene (c,t) $$ (10E)-10-Henicosene #

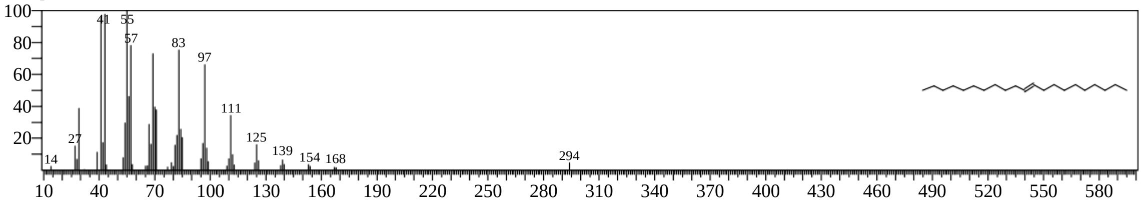

Hit#:4 Entry:31116 Library:NIST20R.lib
SI:96 Formula:C18H36 CAS:112-88-9 MolWeight:252 RetIndex:1801
CompName:1-Octadecene $$ .alpha.-Octadecene $$ Octadecylene, .alpha.- $$ Neodene 18 $$ Octadec-1-ene $$ Octadecene-1 $$ Octadeca-1-ene

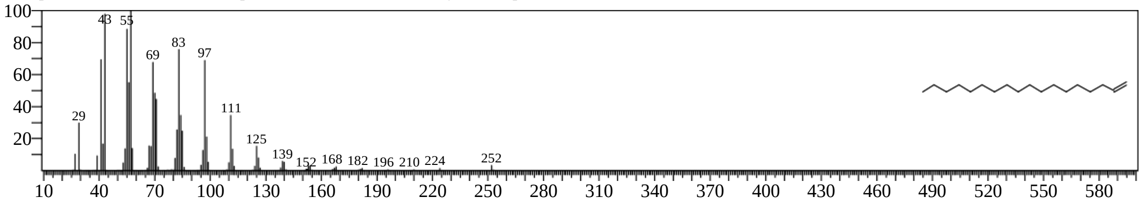

Hit#:5 Entry:29353 Library:NIST20R.lib
SI:96 Formula:C17H34 CAS:6765-39-5 MolWeight:238 RetIndex:1701
CompName:1-Heptadecene $$ Hexahydroaplotaxene $$ Heptadec-1-ene $$ NSC 77132 $$ Heptadecene (Related)

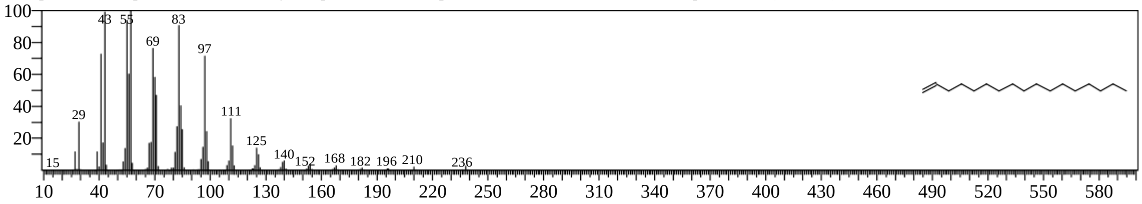

<< Target >>
Line#:7 R.Time:22.400(Scan#:1195) MassPeaks:264
RawMode:Averaged 22.383-22.417(1194-1196) BasePeak:43.10(6225)
BG Mode:Calc. from Peak Group 1 - Event 1 Scan

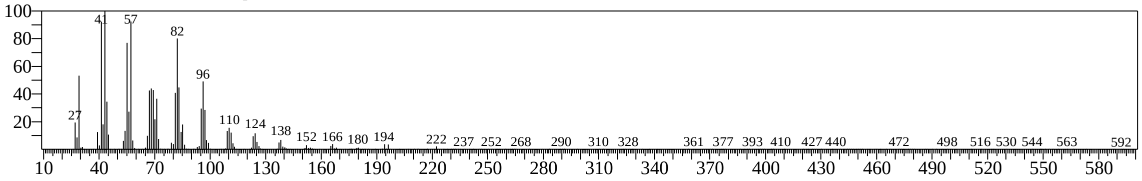

Hit#:1 Entry:125846 Library:NIST20M1.lib
SI:96 Formula:C18H36O CAS:638-66-4 MolWeight:268 RetIndex:1999
CompName:Octadecanal $$ Stearaldehyde $$ Octadecyl aldehyde $$ Stearyl aldehyde $$ n-Octadecanal

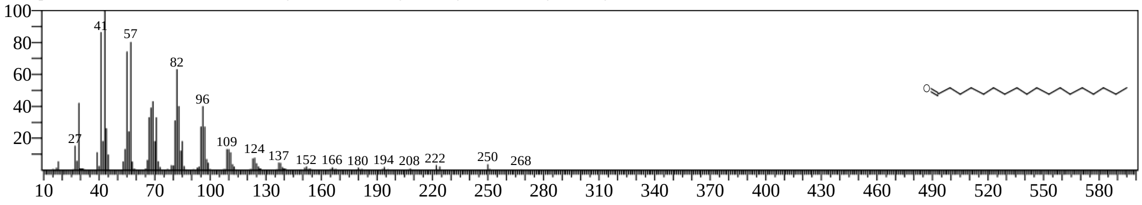

Hit#:2 Entry:25275 Library:NIST20R.lib
SI:96 Formula:C14H28O CAS:124-25-4 MolWeight:212 RetIndex:1601
CompName:Tetradecanal $$ Myristaldehyde $$ Myristylaldehyde $$ Tetradecylaldehyde $$ n-Tetradecanal $$ Aldehyde C-14 $$ Aldehyde C-14, myristic $

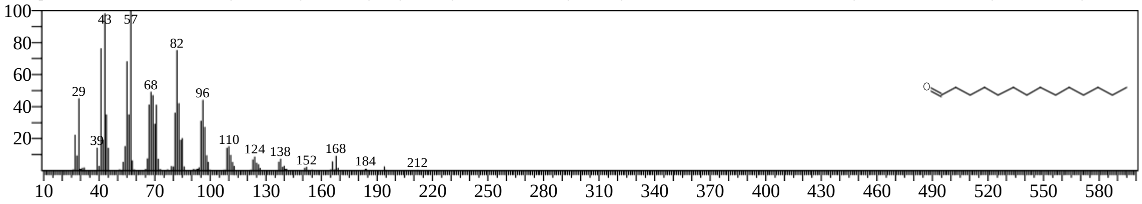

Hit#:3 Entry:25276 Library:NIST20R.lib
SI:96 Formula:C14H28O CAS:124-25-4 MolWeight:212 RetIndex:1601
CompName:Tetradecanal $$ Myristaldehyde $$ Myristylaldehyde $$ Tetradecylaldehyde $$ n-Tetradecanal $$ Aldehyde C-14 $$ Aldehyde C-14, myristic $

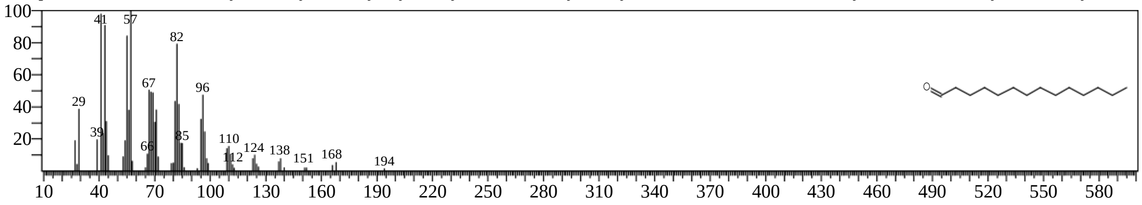

Hit#:4 Entry:25278 Library:NIST20R.lib
SI:95 Formula:C14H28O CAS:124-25-4 MolWeight:212 RetIndex:1601
CompName:Tetradecanal $$ Myristaldehyde $$ Myristylaldehyde $$ Tetradecylaldehyde $$ n-Tetradecanal $$ Aldehyde C-14 $$ Aldehyde C-14, myristic $

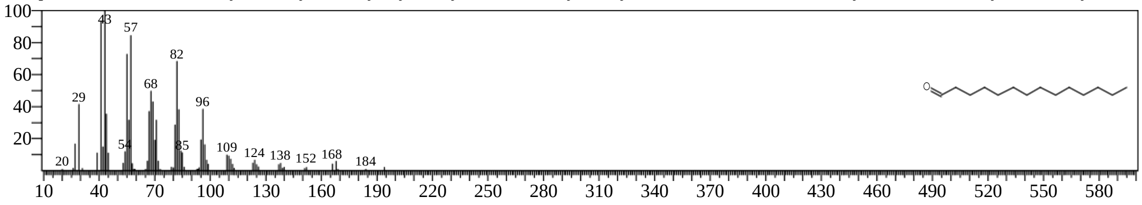

Hit#:5 Entry:109833 Library:NIST20M1.lib
SI:95 Formula:C17H34O CAS:0-00-0 MolWeight:254 RetIndex:1899
CompName:Heptadecanal

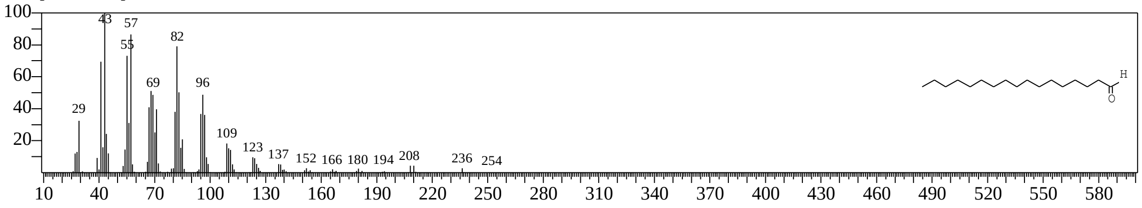

<< Target >>
Line#:8 R.Time:23.683(Scan#:1272) MassPeaks:296
RawMode:Averaged 23.667-23.700(1271-1273) BasePeak:178.05(2888)
BG Mode:Calc. from Peak Group 1 - Event 1 Scan

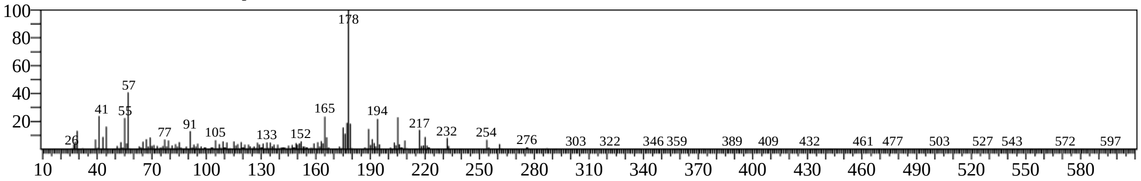

Hit#:1 Entry:135154 Library:NIST20M1.lib
SI:64 Formula:C17H24O3 CAS:82304-66-3 MolWeight:276 RetIndex:2081
CompName:7,9-Di-tert-butyl-1-oxaspiro(4,5)deca-6,9-diene-2,8-dione $$ 1-Oxa-spiro[4.5]deca-6,9-diene-2,8-dione, 7,9-di-tert-butyl- $$ 7,9-Di-tert-butyl-1

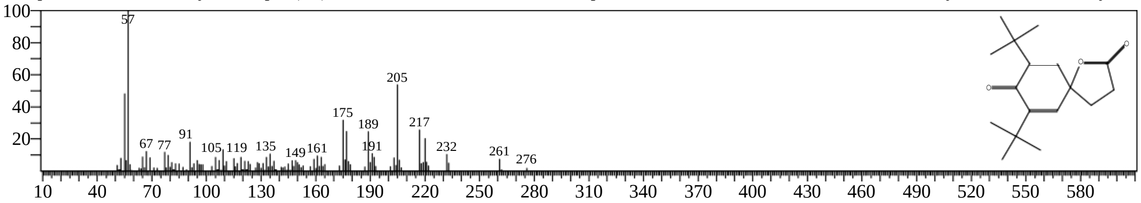

Hit#:2 Entry:33672 Library:NIST20R.lib
SI:63 Formula:C17H24O3 CAS:82304-66-3 MolWeight:276 RetIndex:2081
CompName:7,9-Di-tert-butyl-1-oxaspiro(4,5)deca-6,9-diene-2,8-dione $$ 1-Oxa-spiro[4.5]deca-6,9-diene-2,8-dione, 7,9-di-tert-butyl- $$ 7,9-Di-tert-butyl-1

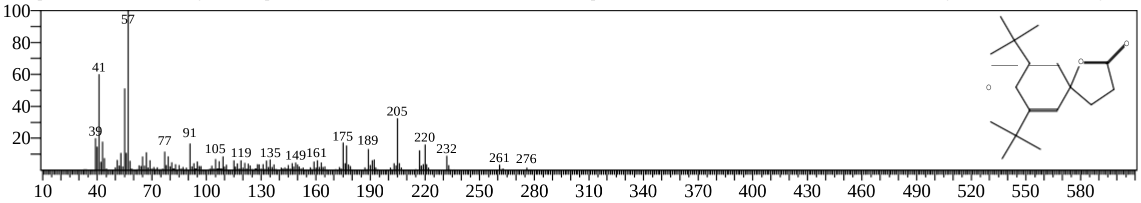

Hit#:3 Entry:222371 Library:NIST20M1.lib
SI:62 Formula:C22H25ClSi CAS:0-00-0 MolWeight:352 RetIndex:2151
CompName:2-Silabicyclo[2.2.2]-[5.6,7.8]dibenzo-2-chlor-3-neopentyl-2-vinyl-octa-5,7-diene

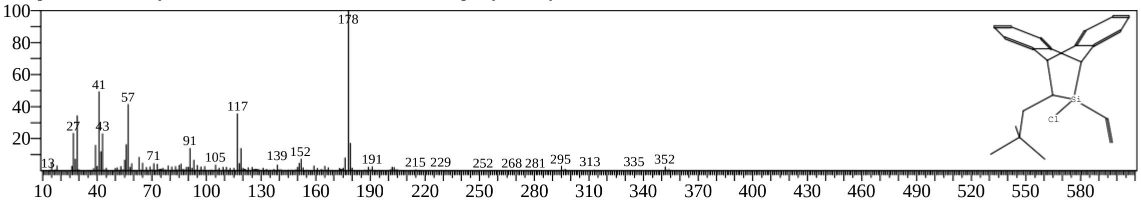

Hit#:4 Entry:72563 Library:NIST20M1.lib
SI:61 Formula:C13H16O3 CAS:84945-01-7 MolWeight:220 RetIndex:1974
CompName:Coumarin-6-ol, 3,4-dihydro-4,4,7,8-tetramethyl- $$ 6-Hydroxy-4,4,7,8-tetramethyl-2-chromanone #

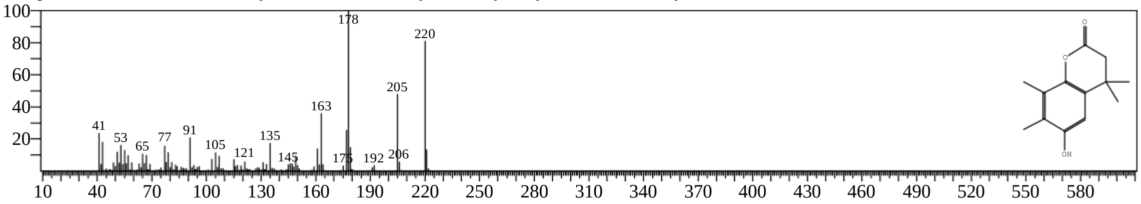

Hit#:5 Entry:224623 Library:NIST20M1.lib
SI:61 Formula:C24H34O2 CAS:27996-19-6 MolWeight:354 RetIndex:2757
CompName:2-tert-Butyl-6-(3-tert-butyl-2-methoxy-5-methylbenzyl)-4-methylphenol

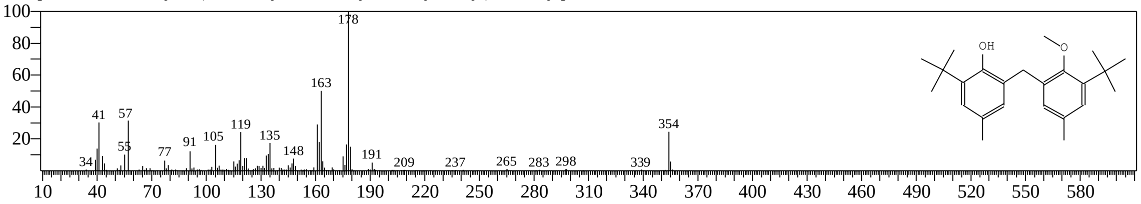

<< Target >>
Line#:9 R.Time:24.483(Scan#:1320) MassPeaks:439
RawMode:Averaged 24.467-24.500(1319-1321) BasePeak:43.10(8508)
BG Mode:Calc. from Peak Group 1 - Event 1 Scan

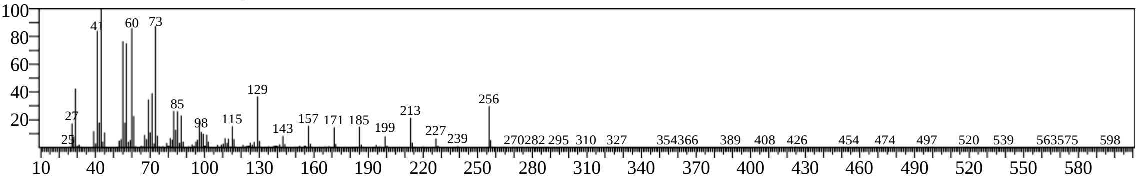

Hit#:1 Entry:31598 Library:NIST20R.lib
SI:96 Formula:C16H32O2 CAS:57-10-3 MolWeight:256 RetIndex:1968
CompName:n-Hexadecanoic acid $$ Hexadecanoic acid $$ n-Hexadecoic acid $$ Palmitic acid $$ Pentadecanecarboxylic acid $$ 1-Pentadecanecarboxylic

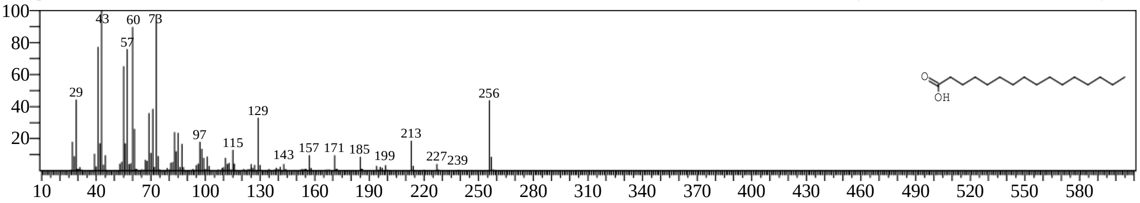

Hit#:2 Entry:31601 Library:NIST20R.lib
SI:95 Formula:C16H32O2 CAS:57-10-3 MolWeight:256 RetIndex:1968
CompName:n-Hexadecanoic acid $$ Hexadecanoic acid $$ n-Hexadecoic acid $$ Palmitic acid $$ Pentadecanecarboxylic acid $$ 1-Pentadecanecarboxylic

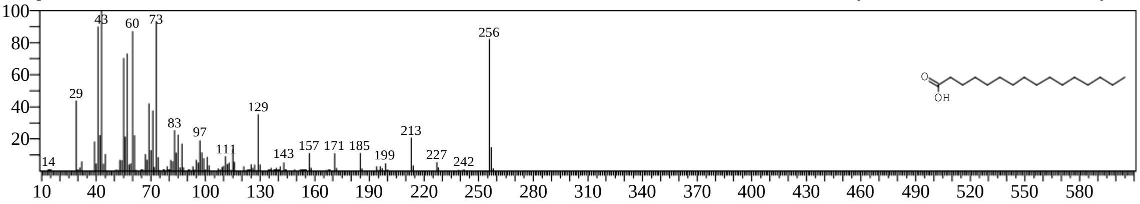

Hit#:3 Entry:96603 Library:NIST20M1.lib
SI:94 Formula:C15H30O2 CAS:1002-84-2 MolWeight:242 RetIndex:1869
CompName:Pentadecanoic acid $$ Pentadecylic acid $$ n-Pentadecanoic acid $$ n-Pentadecylic acid

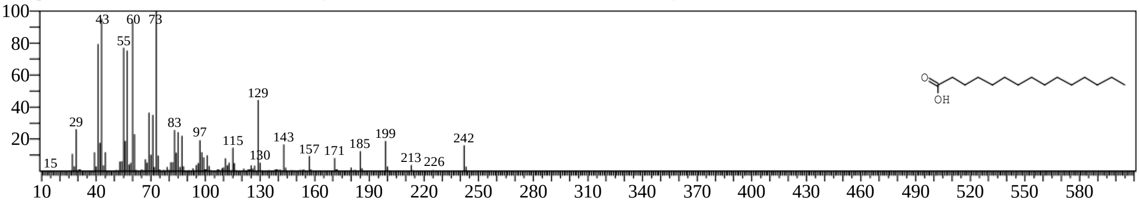

Hit#:4 Entry:112116 Library:NIST20M1.lib
SI:93 Formula:C16H32O2 CAS:57-10-3 MolWeight:256 RetIndex:1968
CompName:n-Hexadecanoic acid $$ Hexadecanoic acid $$ n-Hexadecoic acid $$ Palmitic acid $$ Pentadecanecarboxylic acid $$ 1-Pentadecanecarboxylic

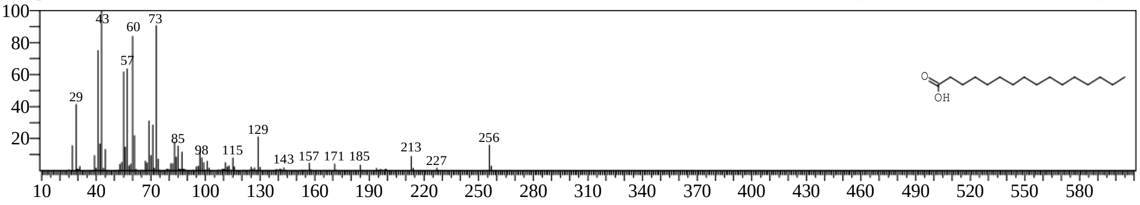

Hit#:5 Entry:31599 Library:NIST20R.lib
SI:92 Formula:C16H32O2 CAS:57-10-3 MolWeight:256 RetIndex:1968
CompName:n-Hexadecanoic acid $$ Hexadecanoic acid $$ n-Hexadecoic acid $$ Palmitic acid $$ Pentadecanecarboxylic acid $$ 1-Pentadecanecarboxylic

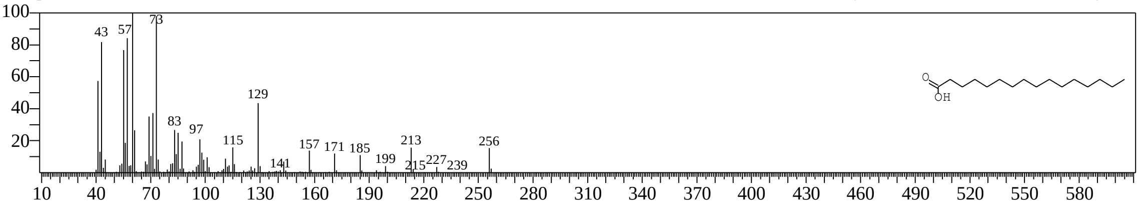

<< Target >>
Line#:10 R.Time:24.967(Scan#:1349) MassPeaks:314
RawMode:Averaged 24.950-24.983(1348-1350) BasePeak:43.10(34906)
BG Mode:Calc. from Peak Group 1 - Event 1 Scan

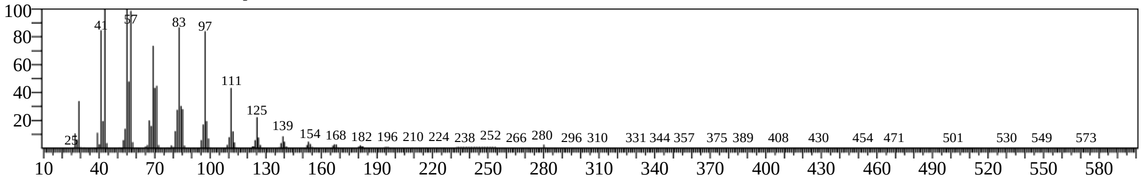

Hit#:1 Entry:38649 Library:NIST20R.lib
SI:96 Formula:C24H48 CAS:10192-32-2 MolWeight:336 RetIndex:2397
CompName:1-Tetracosene

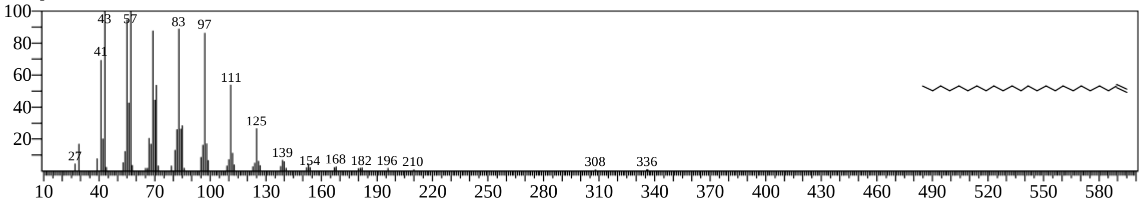

Hit#:2 Entry:32651 Library:NIST20R.lib
SI:96 Formula:C19H38 CAS:18435-45-5 MolWeight:266 RetIndex:1900
CompName:1-Nonadecene $$ Nonadec-1-ene

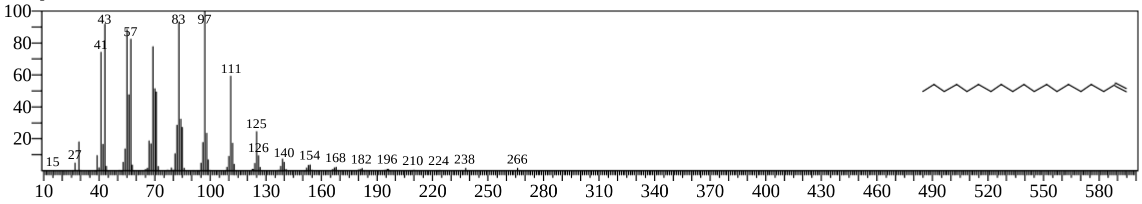

Hit#:3 Entry:29353 Library:NIST20R.lib
SI:96 Formula:C17H34 CAS:6765-39-5 MolWeight:238 RetIndex:1701
CompName:1-Heptadecene $$ Hexahydroaplotaxene $$ Heptadec-1-ene $$ NSC 77132 $$ Heptadecene (Related)

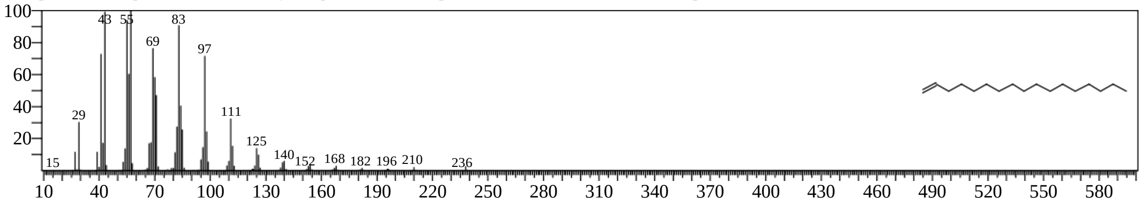

Hit#:4 Entry:40183 Library:NIST20R.lib
SI:96 Formula:C26H52 CAS:18835-33-1 MolWeight:364 RetIndex:2596
CompName:1-Hexacosene

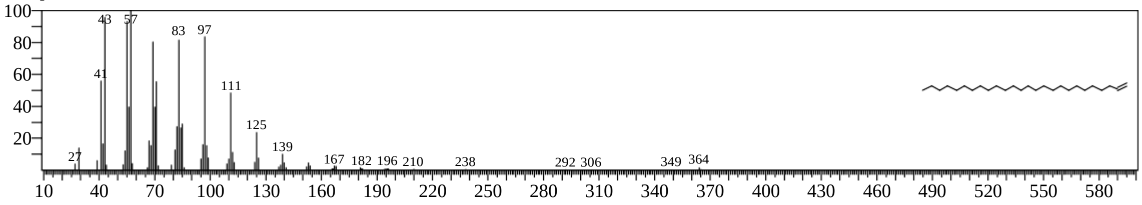

Hit#:5 Entry:37645 Library:NIST20R.lib
SI:96 Formula:C23H46 CAS:27519-02-4 MolWeight:322 RetIndex:2315
CompName:9-Tricosene, (Z)- $$ (Z)-9-Tricosene $$ cis-9-Tricosene $$ Muscalure $$ (9Z)-Tricosene $$ (9Z)-9-Tricosene #

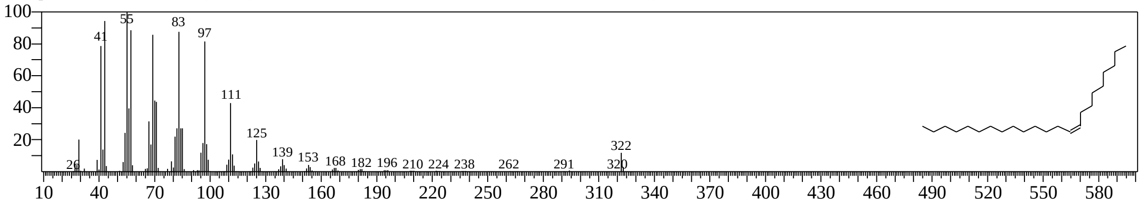

<< Target >>
Line#:11 R.Time:25.050(Scan#:1354) MassPeaks:319
RawMode:Averaged 25.033-25.067(1353-1355) BasePeak:43.10(1600)
BG Mode:Calc. from Peak Group 1 - Event 1 Scan

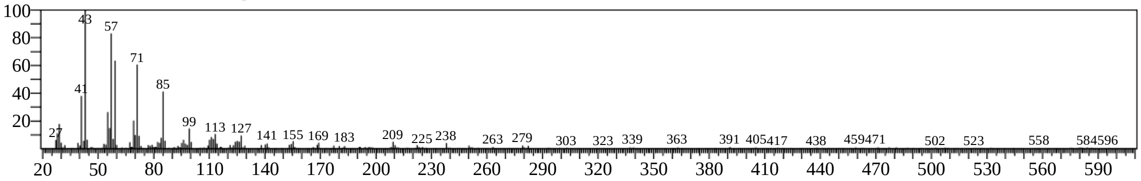

Hit#:1 Entry:248622 Library:NIST20M1.lib
SI:87 Formula:C24H46O3 CAS:0-00-0 MolWeight:382 RetIndex:2617
CompName:Carbonic acid, eicosyl prop-1-en-2-yl ester

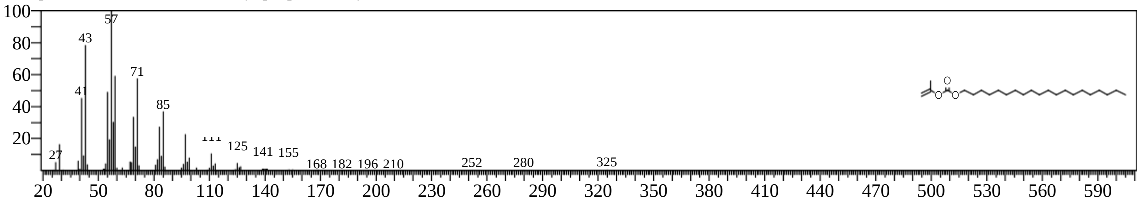

Hit#:2 Entry:224510 Library:NIST20M1.lib
SI:86 Formula:C22H42O3 CAS:0-00-0 MolWeight:354 RetIndex:2418
CompName:Carbonic acid, octadecyl prop-1-en-2-yl ester

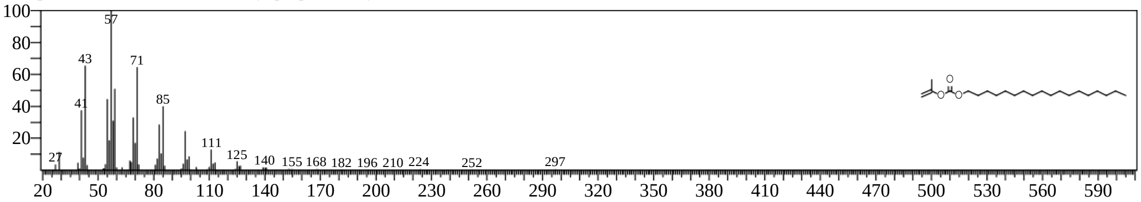

Hit#:3 Entry:210056 Library:NIST20M1.lib
SI:86 Formula:C21H40O3 CAS:0-00-0 MolWeight:340 RetIndex:2319
CompName:Carbonic acid, heptadecyl prop-1-en-2-yl ester

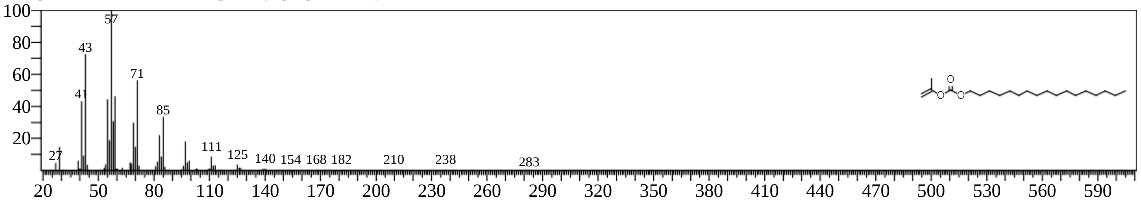

Hit#:4 Entry:161493 Library:NIST20M1.lib
SI:86 Formula:C18H34O3 CAS:0-00-0 MolWeight:298 RetIndex:2021
CompName:Carbonic acid, prop-1-en-2-yl tetradecyl ester

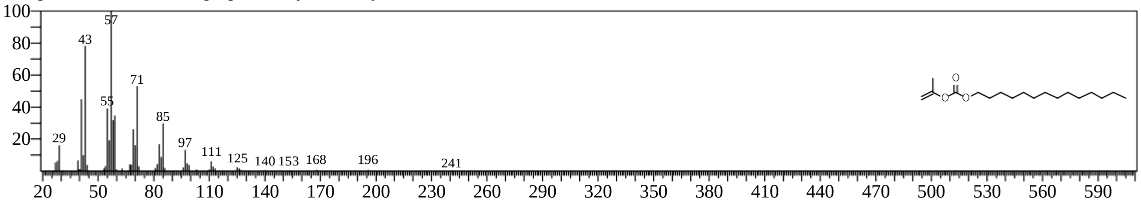

Hit#:5 Entry:142257 Library:NIST20M1.lib
SI:86 Formula:C20H42 CAS:1560-86-7 MolWeight:282 RetIndex:1945
CompName:Nonadecane, 2-methyl- $$ 2-Methylnonadecane

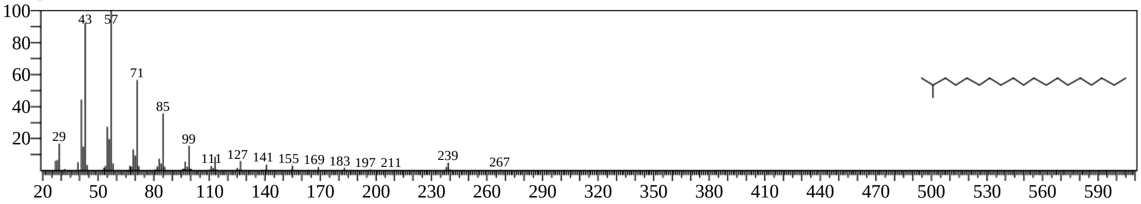

<< Target >>
Line#:12 R.Time:25.333(Scan#:1371) MassPeaks:324
RawMode:Averaged 25.317-25.350(1370-1372) BasePeak:43.10(14398)
BG Mode:Calc. from Peak Group 1 - Event 1 Scan

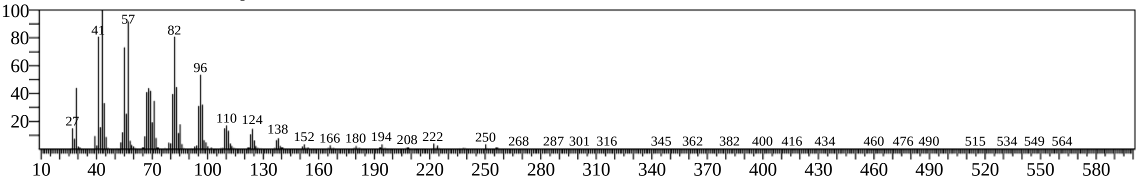

Hit#:1 Entry:125846 Library:NIST20M1.lib
SI:96 Formula:C18H36O CAS:638-66-4 MolWeight:268 RetIndex:1999
CompName:Octadecanal $$ Stearaldehyde $$ Octadecyl aldehyde $$ Stearyl aldehyde $$ n-Octadecanal

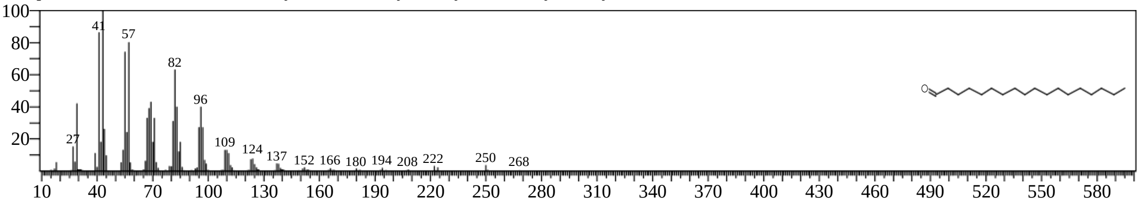

Hit#:2 Entry:109833 Library:NIST20M1.lib
SI:96 Formula:C17H34O CAS:0-00-0 MolWeight:254 RetIndex:1899
CompName:Heptadecanal

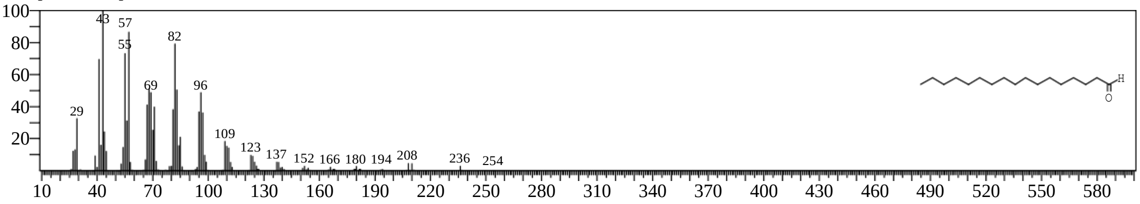

Hit#:3 Entry:25275 Library:NIST20R.lib
SI:95 Formula:C14H28O CAS:124-25-4 MolWeight:212 RetIndex:1601
CompName:Tetradecanal $$ Myristaldehyde $$ Myristylaldehyde $$ Tetradecylaldehyde $$ n-Tetradecanal $$ Aldehyde C-14 $$ Aldehyde C-14, myristic $

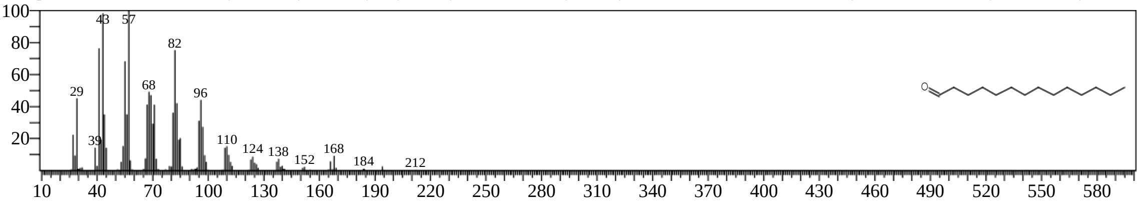

Hit#:4 Entry:25276 Library:NIST20R.lib
SI:95 Formula:C14H28O CAS:124-25-4 MolWeight:212 RetIndex:1601
CompName:Tetradecanal $$ Myristaldehyde $$ Myristylaldehyde $$ Tetradecylaldehyde $$ n-Tetradecanal $$ Aldehyde C-14 $$ Aldehyde C-14, myristic $

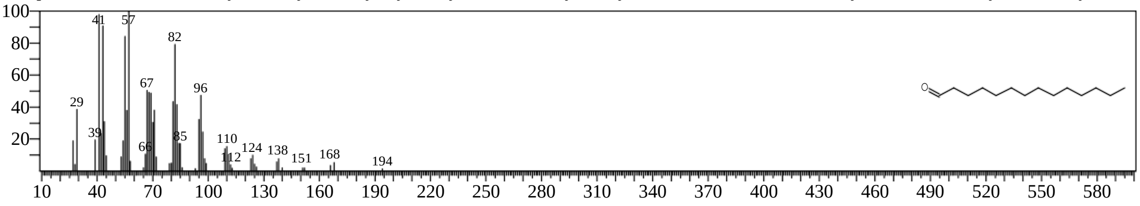

Hit#:5 Entry:32890 Library:NIST20R.lib
SI:95 Formula:C18H36O CAS:638-66-4 MolWeight:268 RetIndex:1999
CompName:Octadecanal $$ Stearaldehyde $$ Octadecyl aldehyde $$ Stearyl aldehyde $$ n-Octadecanal

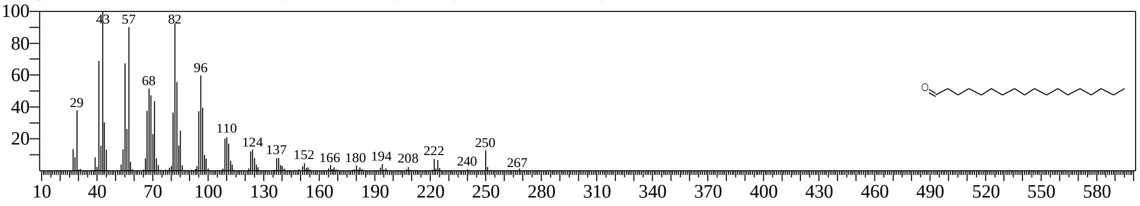

<< Target >>
Line#:13 R.Time:26.450(Scan#:1438) MassPeaks:314
RawMode:Averaged 26.433-26.467(1437-1439) BasePeak:57.10(3008)
BG Mode:Calc. from Peak Group 1 - Event 1 Scan

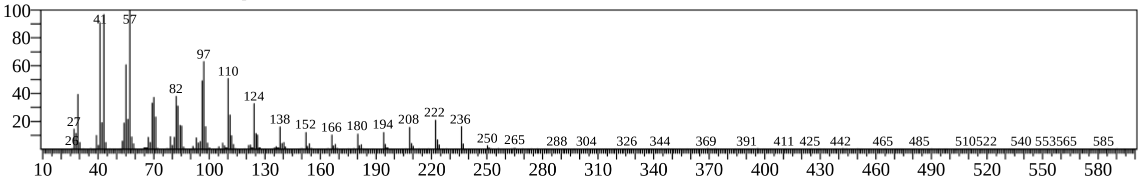

Hit#:1 Entry:32527 Library:NIST20R.lib
SI:94 Formula:C18H35N CAS:638-65-3 MolWeight:265 RetIndex:2056
CompName:Octadecanenitrile $$ Stearonitrile $$ Heptadecyl cyanide $$ Nitril kyseliny stearove $$ 1-Cyanoheptadecane $$ Octadecanonitrile $$ Oktadeka

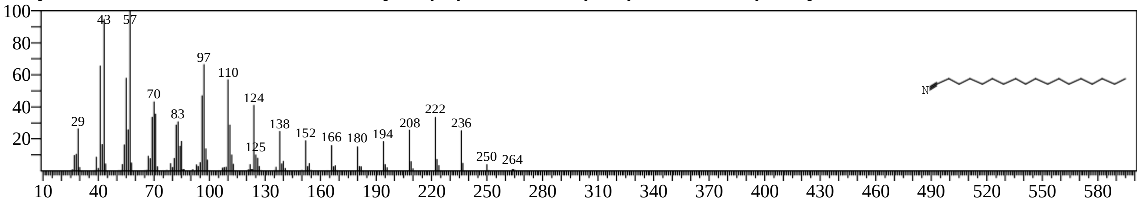

Hit#:2 Entry:30956 Library:NIST20R.lib
SI:93 Formula:C17H33N CAS:5399-02-0 MolWeight:251 RetIndex:1956
CompName:Heptadecanenitrile $$ Hexadecyl cyanide $$ Heptadecanonitrile $$ Heptadecanoic acid nitrile $$ Cetyl cyanide $$ 1-Cyanohexadecane $$ Mar

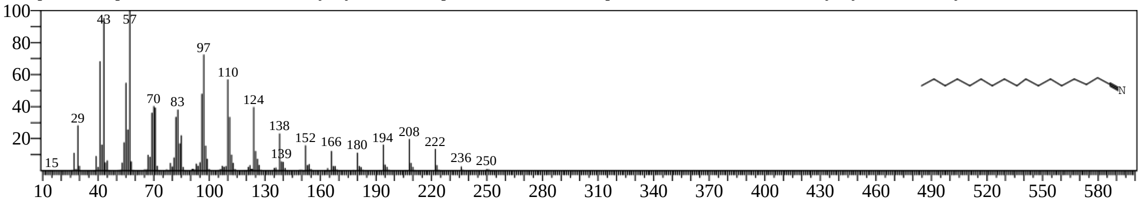

Hit#:3 Entry:29157 Library:NIST20R.lib
SI:93 Formula:C16H31N CAS:629-79-8 MolWeight:237 RetIndex:1857
CompName:Hexadecanenitrile $$ Palmitonitrile $$ Palmitic acid, nitrile $$ 1-Cyanopentadecane

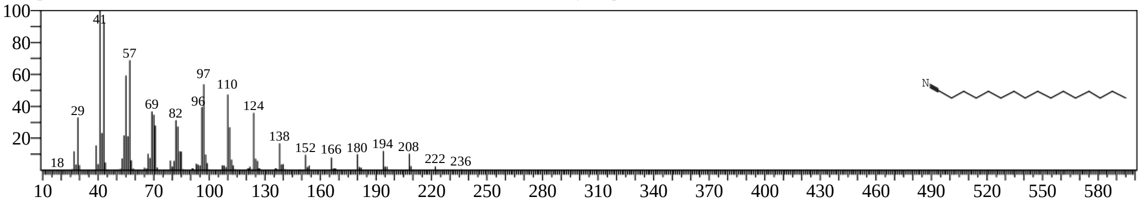

Hit#:4 Entry:29155 Library:NIST20R.lib
SI:92 Formula:C16H31N CAS:629-79-8 MolWeight:237 RetIndex:1857
CompName:Hexadecanenitrile $$ Palmitonitrile $$ Palmitic acid, nitrile $$ 1-Cyanopentadecane

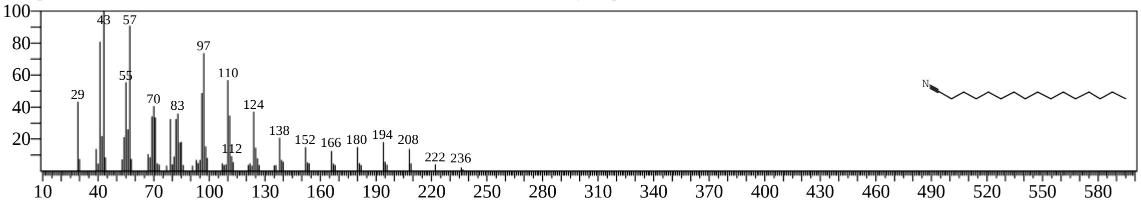

Hit#:5 Entry:27174 Library:NIST20R.lib
SI:92 Formula:C15H29N CAS:18300-91-9 MolWeight:223 RetIndex:1758
CompName:Pentadecanenitrile $$ Tetradecyl cyanide $$ Pentadecanitrile $$ n-Pentadecanonitrile $$ Pentadecanonitrile

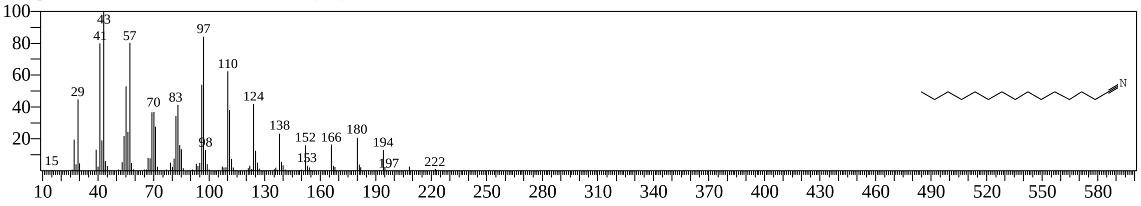

<< Target >>
Line#:14 R.Time:27.183(Scan#:1482) MassPeaks:321
RawMode:Averaged 27.167-27.200(1481-1483) BasePeak:43.10(1146)
BG Mode:Calc. from Peak Group 1 - Event 1 Scan

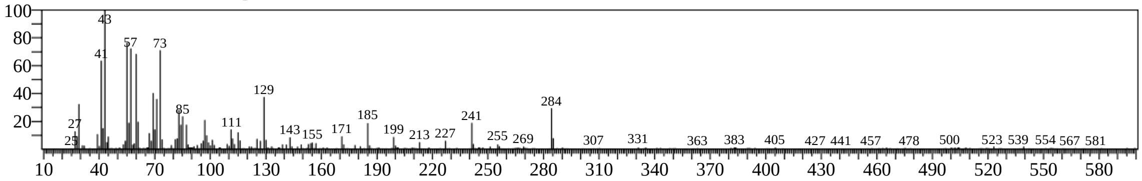

Hit#:1 Entry:34460 Library:NIST20R.lib
SI:94 Formula:C18H36O2 CAS:57-11-4 MolWeight:284 RetIndex:2167
CompName:Octadecanoic acid $$ Stearic acid $$ n-Octadecanoic acid $$ Humko Industrene R $$ Hydrofol Acid 150 $$ Hystrene S-97 $$ Hystrene T-70 $

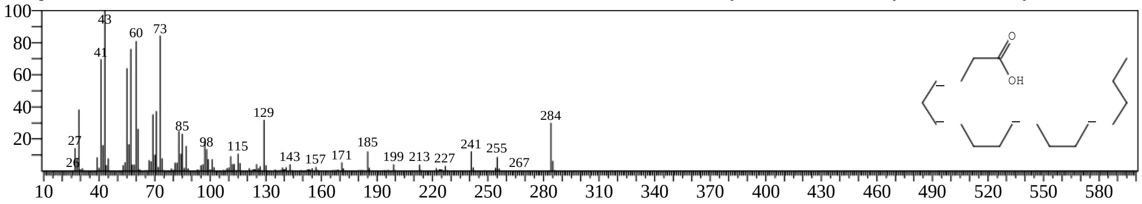

Hit#:2 Entry:34462 Library:NIST20R.lib
SI:94 Formula:C18H36O2 CAS:57-11-4 MolWeight:284 RetIndex:2167
CompName:Octadecanoic acid $$ Stearic acid $$ n-Octadecanoic acid $$ Humko Industrene R $$ Hydrofol Acid 150 $$ Hystrene S-97 $$ Hystrene T-70 $

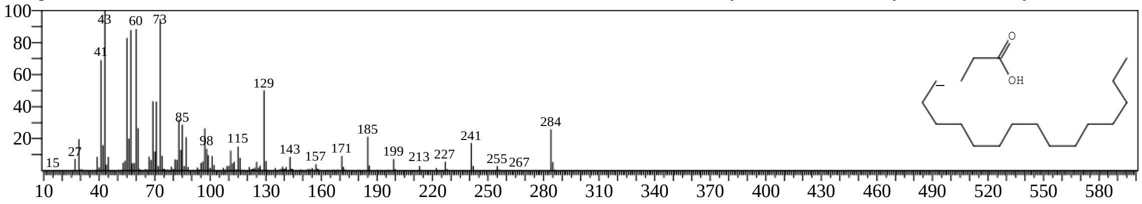

Hit#:3 Entry:34461 Library:NIST20R.lib
SI:93 Formula:C18H36O2 CAS:57-11-4 MolWeight:284 RetIndex:2167
CompName:Octadecanoic acid $$ Stearic acid $$ n-Octadecanoic acid $$ Humko Industrene R $$ Hydrofol Acid 150 $$ Hystrene S-97 $$ Hystrene T-70 $

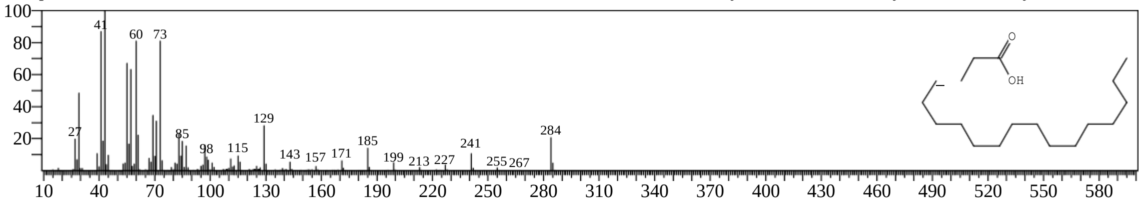

Hit#:4 Entry:144781 Library:NIST20M1.lib
SI:92 Formula:C18H36O2 CAS:57-11-4 MolWeight:284 RetIndex:2167
CompName:Octadecanoic acid $$ Stearic acid $$ n-Octadecanoic acid $$ Humko Industrene R $$ Hydrofol Acid 150 $$ Hystrene S-97 $$ Hystrene T-70 $

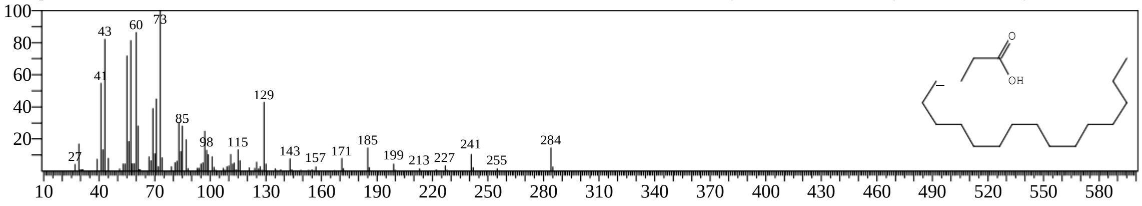

Hit#:5 Entry:36903 Library:NIST20R.lib
SI:91 Formula:C20H40O2 CAS:506-30-9 MolWeight:312 RetIndex:2366
CompName:Eicosanoic acid $$ Arachic acid $$ Arachidic acid $$ Icosanoic acid $$ n-Eicosanoic acid $$ Arachidic acid (synthetic)

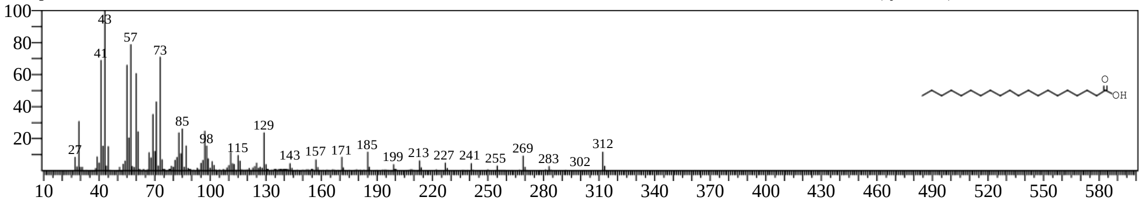

<< Target >>
Line#:15 R.Time:27.633(Scan#:1509) MassPeaks:309
RawMode:Averaged 27.617-27.650(1508-1510) BasePeak:57.10(20442)
BG Mode:Calc. from Peak Group 1 - Event 1 Scan

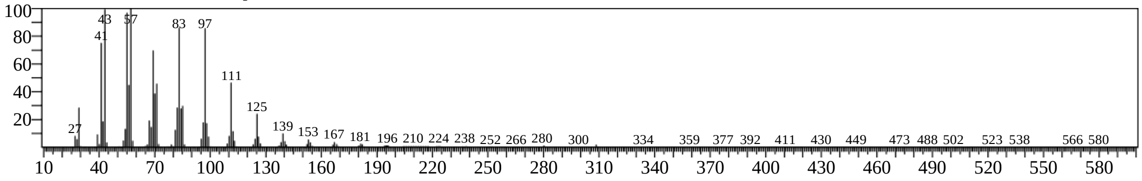

Hit#:1 Entry:38649 Library:NIST20R.lib
SI:97 Formula:C24H48 CAS:10192-32-2 MolWeight:336 RetIndex:2397
CompName:1-Tetracosene

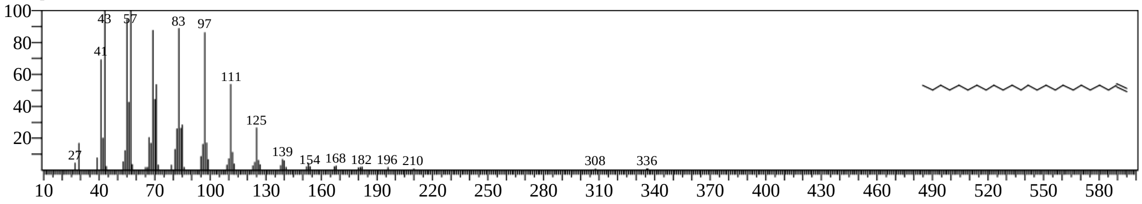

Hit#:2 Entry:40183 Library:NIST20R.lib
SI:97 Formula:C26H52 CAS:18835-33-1 MolWeight:364 RetIndex:2596
CompName:1-Hexacosene

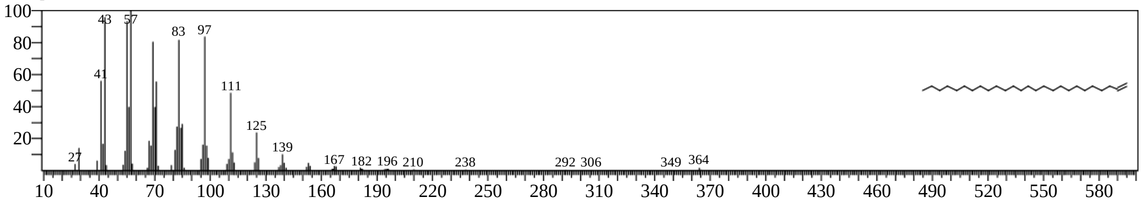

Hit#:3 Entry:32651 Library:NIST20R.lib
SI:96 Formula:C19H38 CAS:18435-45-5 MolWeight:266 RetIndex:1900
CompName:1-Nonadecene $$ Nonadec-1-ene

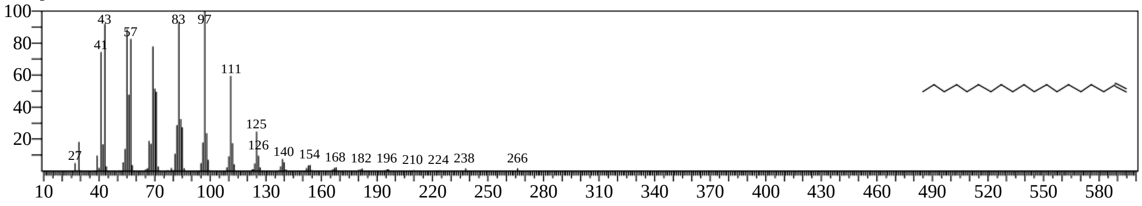

Hit#:4 Entry:178229 Library:NIST20M1.lib
SI:96 Formula:C21H44O CAS:15594-90-8 MolWeight:312 RetIndex:2351
CompName:1-Heneicosanol $$ Henicosan-1-ol $$ Heneicosyl alcohol

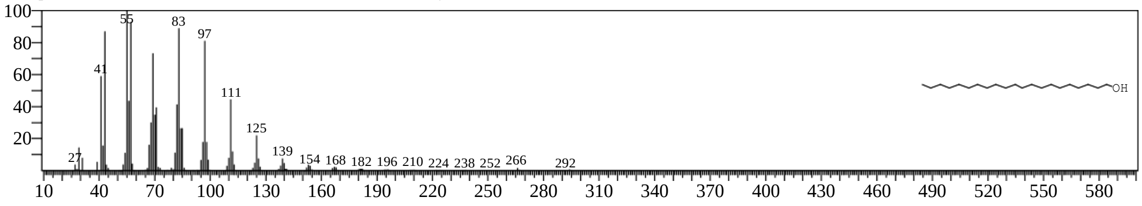

Hit#:5 Entry:37648 Library:NIST20R.lib
SI:96 Formula:C23H46 CAS:18835-32-0 MolWeight:322 RetIndex:2298
CompName:1-Tricosene $$ Tricosene-1

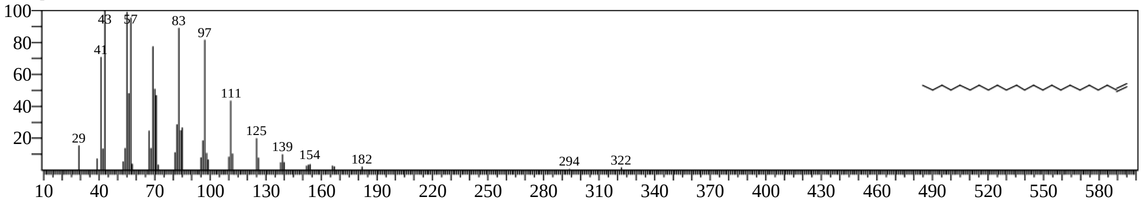

<< Target >>
Line#:16 R.Time:30.067(Scan#:1655) MassPeaks:289
RawMode:Averaged 30.050-30.083(1654-1656) BasePeak:57.10(11954)
BG Mode:Calc. from Peak Group 1 - Event 1 Scan

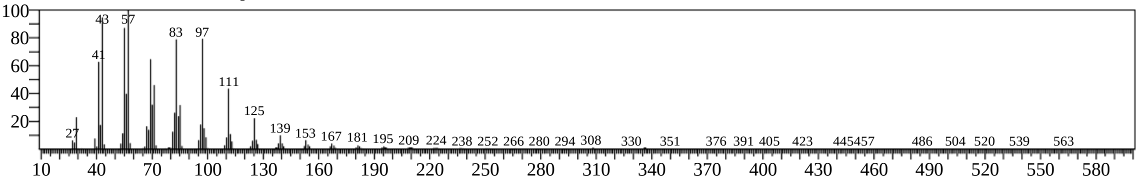

Hit#:1 Entry:40183 Library:NIST20R.lib
SI:97 Formula:C26H52 CAS:18835-33-1 MolWeight:364 RetIndex:2596
CompName:1-Hexacosene

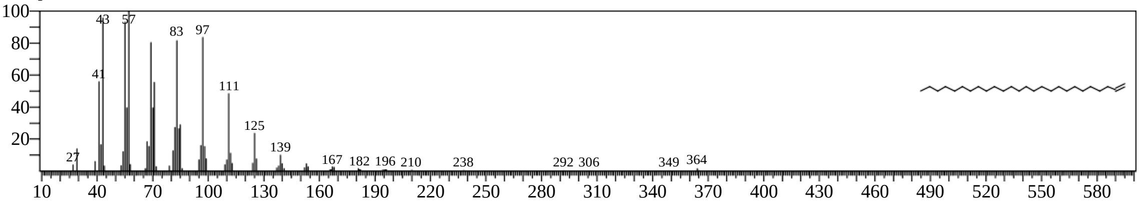

Hit#:2 Entry:221995 Library:NIST20M1.lib
SI:97 Formula:C19H35F3O2 CAS:0-00-0 MolWeight:352 RetIndex:1911
CompName:Heptadecyl trifluoroacetate $$ Heptadecyl 2,2,2-trifluoroacetate $$ 1-Heptadecanol, trifluoroacetate $$ Trifluoroacetic acid, n-heptadecyl ester

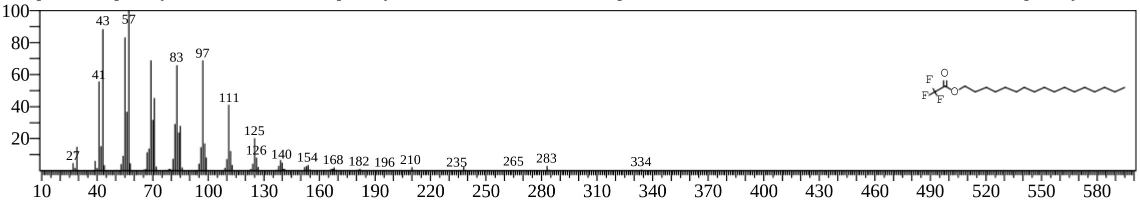

Hit#:3 Entry:207144 Library:NIST20M1.lib
SI:96 Formula:C18H33F3O2 CAS:6222-03-3 MolWeight:338 RetIndex:1812
CompName:Trifluoroacetoxy hexadecane $$ Hexadecyl trifluoroacetate $$ Hexadecyl 2,2,2-trifluoroacetate $$ 1-Hexadecanol, trifluoroacetate $$ Acetic ac

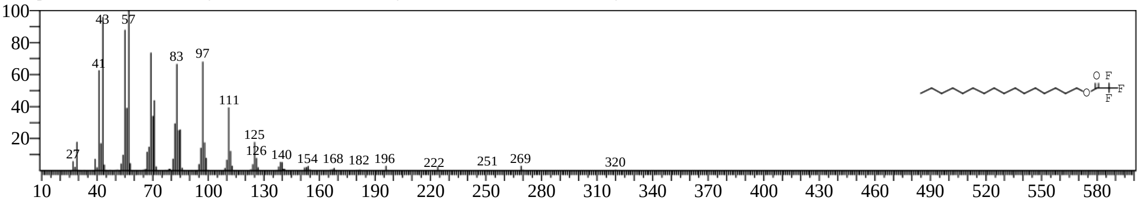

Hit#:4 Entry:234861 Library:NIST20M1.lib
SI:96 Formula:C20H37F3O2 CAS:79392-43-1 MolWeight:366 RetIndex:2011
CompName:Octadecyl trifluoroacetate $$ Octadecyl 2,2,2-trifluoroacetate $$ 1-Octadecanol, trifluoroacetate $$ Trifluoroacetic acid, n-octadecyl ester $$ A

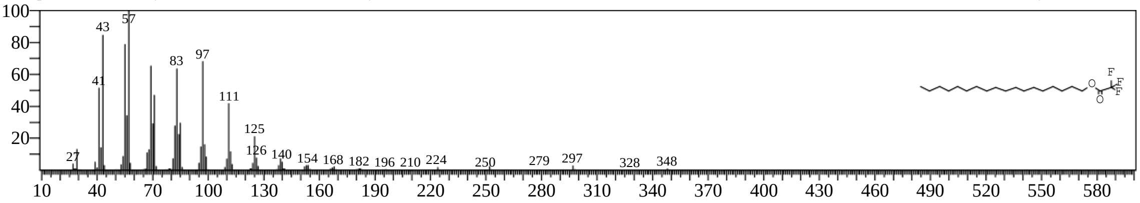

Hit#:5 Entry:41853 Library:NIST20R.lib
SI:96 Formula:C28H58O CAS:557-61-9 MolWeight:410 RetIndex:3047
CompName:Octacosanol $$ 1-Octacosanol $$ n-Octacosanol $$ Cluytyl alcohol $$ Montanyl alcohol $$ Octacosyl alcohol

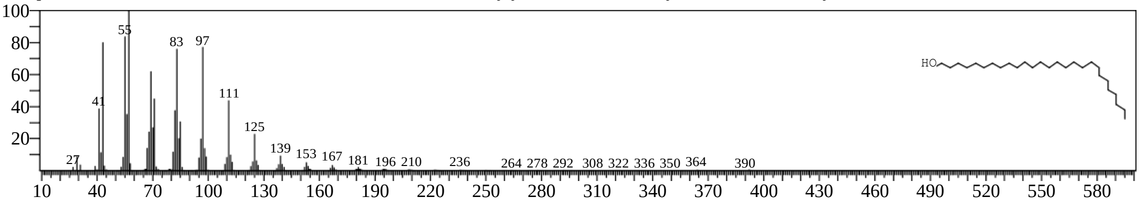

<< Target >>
Line#:17 R.Time:32.333(Scan#:1791) MassPeaks:370
RawMode:Averaged 32.317-32.350(1790-1792) BasePeak:57.10(5944)
BG Mode:Calc. from Peak Group 1 - Event 1 Scan

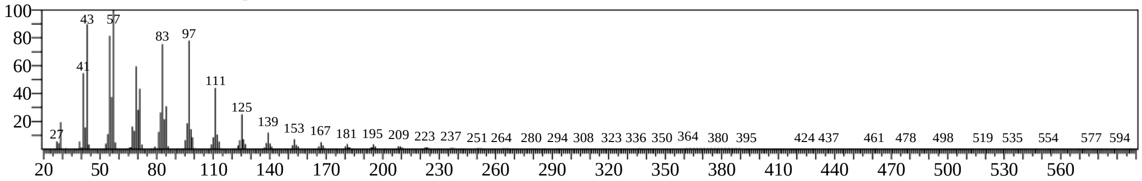

Hit#:1 Entry:41853 Library:NIST20R.lib
SI:97 Formula:C28H58O CAS:557-61-9 MolWeight:410 RetIndex:3047
CompName:Octacosanol $$ 1-Octacosanol $$ n-Octacosanol $$ Cluytyl alcohol $$ Montanyl alcohol $$ Octacosyl alcohol

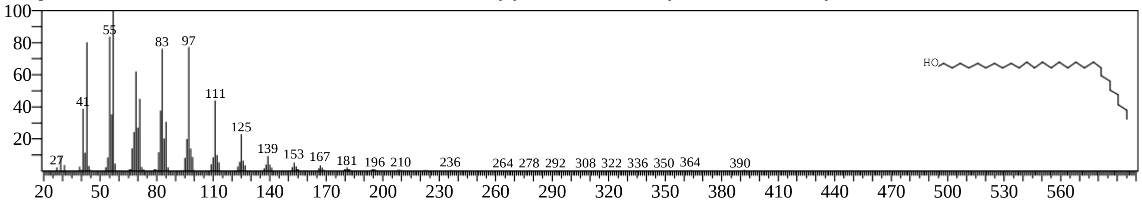

Hit#:2 Entry:234861 Library:NIST20M1.lib
SI:96 Formula:C20H37F3O2 CAS:79392-43-1 MolWeight:366 RetIndex:2011
CompName:Octadecyl trifluoroacetate $$ Octadecyl 2,2,2-trifluoroacetate $$ 1-Octadecanol, trifluoroacetate $$ Trifluoroacetic acid, n-octadecyl ester $$ A

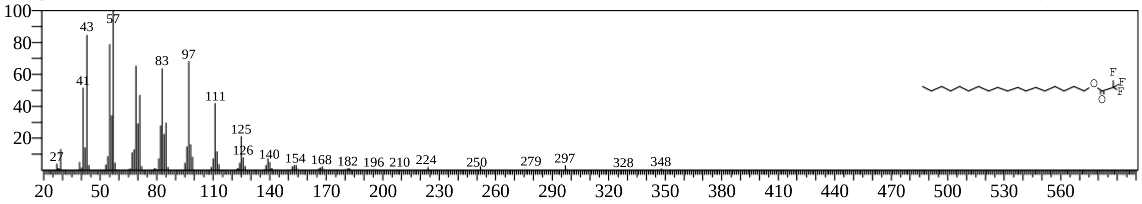

Hit#:3 Entry:221995 Library:NIST20M1.lib
SI:96 Formula:C19H35F3O2 CAS:0-00-0 MolWeight:352 RetIndex:1911
CompName:Heptadecyl trifluoroacetate $$ Heptadecyl 2,2,2-trifluoroacetate $$ 1-Heptadecanol, trifluoroacetate $$ Trifluoroacetic acid, n-heptadecyl ester

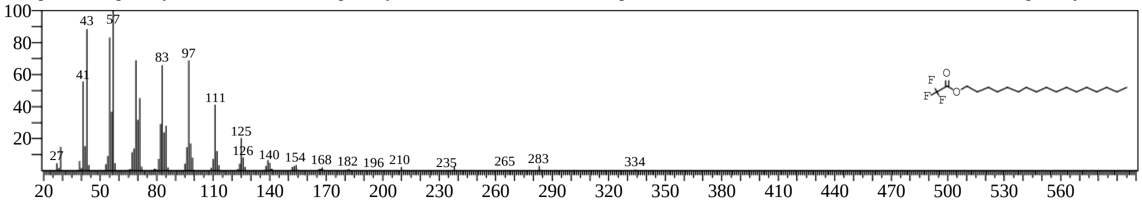

Hit#:4 Entry:40183 Library:NIST20R.lib
SI:96 Formula:C26H52 CAS:18835-33-1 MolWeight:364 RetIndex:2596
CompName:1-Hexacosene

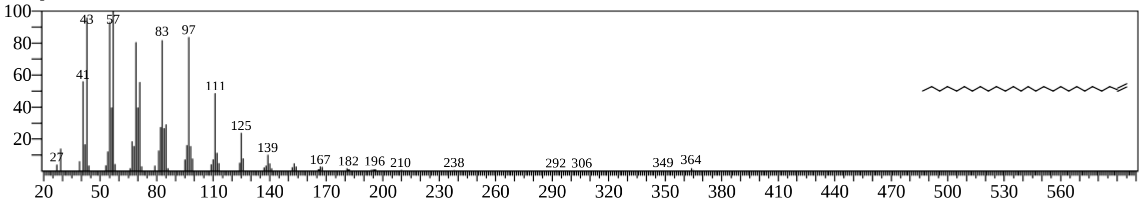

Hit#:5 Entry:1512 Library:NIST20M2.lib
SI:95 Formula:C20H35F5O2 CAS:959218-78-1 MolWeight:402 RetIndex:1872
CompName:Pentafluoropropionic acid, heptadecyl ester

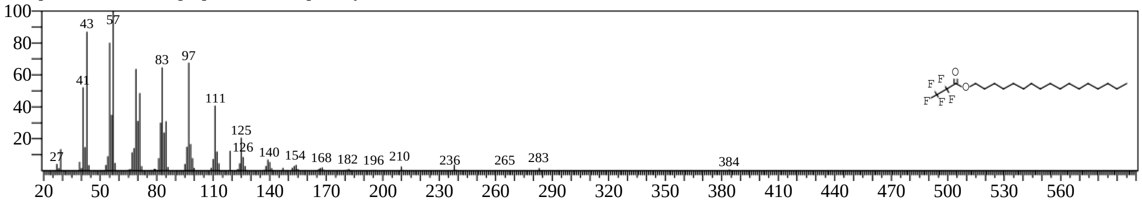

<< Target >>
Line#:18 R.Time:34.450(Scan#:1918) MassPeaks:348
RawMode:Averaged 34.433-34.467(1917-1919) BasePeak:57.10(3085)
BG Mode:Calc. from Peak Group 1 - Event 1 Scan

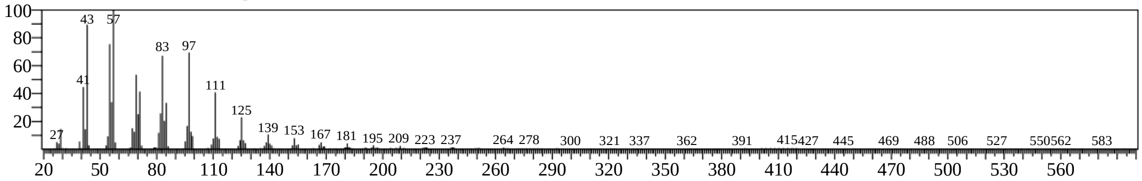

Hit#:1 Entry:234861 Library:NIST20M1.lib
SI:96 Formula:C20H37F3O2 CAS:79392-43-1 MolWeight:366 RetIndex:2011
CompName:Octadecyl trifluoroacetate $$ Octadecyl 2,2,2-trifluoroacetate $$ 1-Octadecanol, trifluoroacetate $$ Trifluoroacetic acid, n-octadecyl ester $$ A

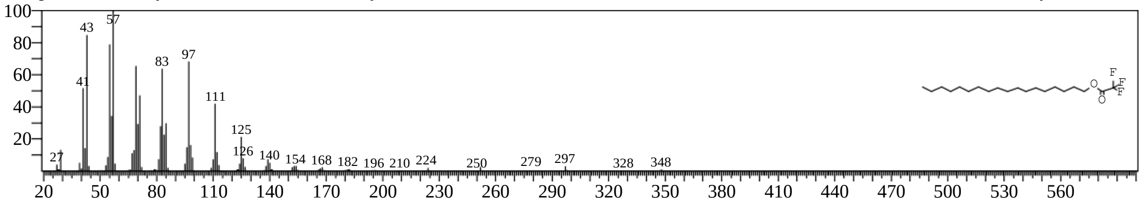

Hit#:2 Entry:41853 Library:NIST20R.lib
SI:96 Formula:C28H58O CAS:557-61-9 MolWeight:410 RetIndex:3047
CompName:Octacosanol $$ 1-Octacosanol $$ n-Octacosanol $$ Cluytyl alcohol $$ Montanyl alcohol $$ Octacosyl alcohol

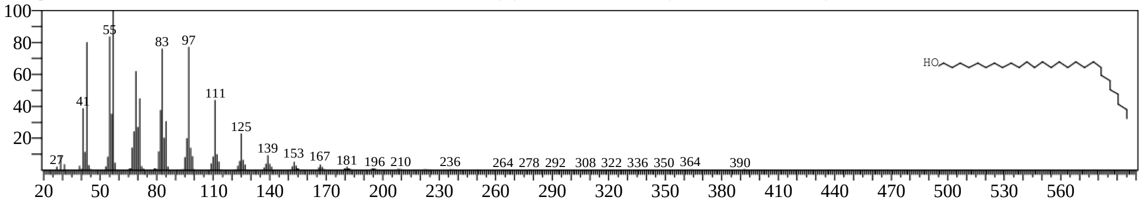

Hit#:3 Entry:17731 Library:NIST20M2.lib
SI:96 Formula:C25H47F3O2 CAS:0-00-0 MolWeight:436 RetIndex:2508
CompName:Tricosyl trifluoroacetate $$ Tricosyl 2,2,2-trifluoroacetate $$ 1-Tricosanol, trifluoroacetate

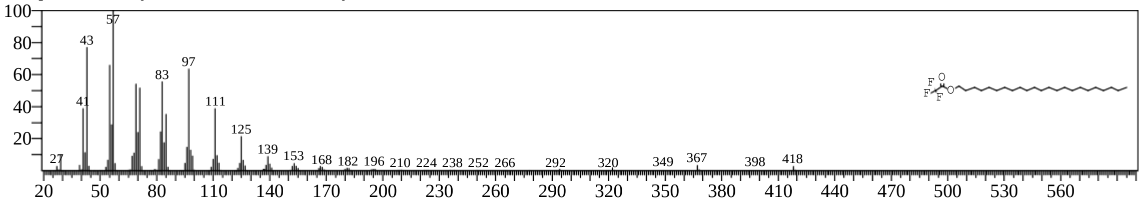

Hit#:4 Entry:35676 Library:NIST20M2.lib
SI:95 Formula:C25H43F7O2 CAS:0-00-0 MolWeight:508 RetIndex:2230
CompName:Heneicosyl heptafluorobutyrate $$ Heneicosyl 2,2,3,3,4,4,4-heptafluorobutanoate $$ 1-Heneicosanol, heptafluorobutyrate $$ Heneicosyl perflu

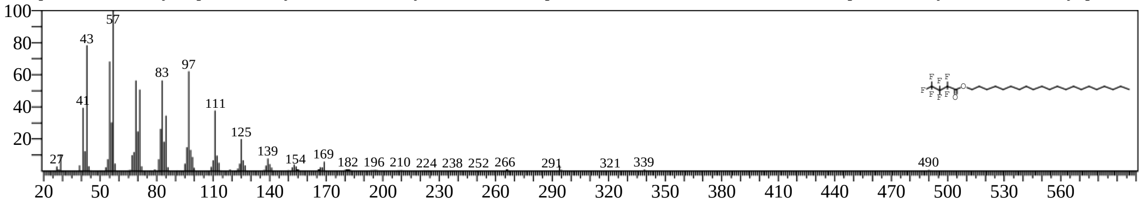

Hit#:5 Entry:221995 Library:NIST20M1.lib
SI:95 Formula:C19H35F3O2 CAS:0-00-0 MolWeight:352 RetIndex:1911
CompName:Heptadecyl trifluoroacetate $$ Heptadecyl 2,2,2-trifluoroacetate $$ 1-Heptadecanol, trifluoroacetate $$ Trifluoroacetic acid, n-heptadecyl ester

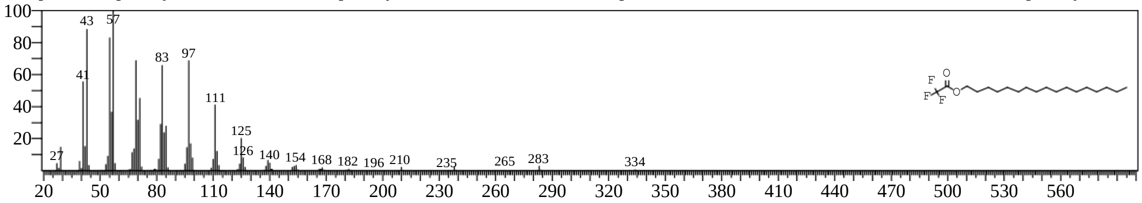

<< Target >>
Line#:19 R.Time:36.350(Scan#:2032) MassPeaks:261
RawMode:Averaged 36.333-36.367(2031-2033) BasePeak:57.10(1866)
BG Mode:Calc. from Peak Group 1 - Event 1 Scan

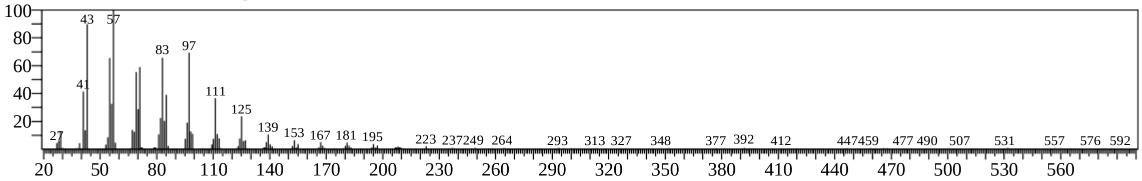

Hit#:1 Entry:17731 Library:NIST20M2.lib
SI:95 Formula:C25H47F3O2 CAS:0-00-0 MolWeight:436 RetIndex:2508
CompName:Tricosyl trifluoroacetate $$ Tricosyl 2,2,2-trifluoroacetate $$ 1-Tricosanol, trifluoroacetate

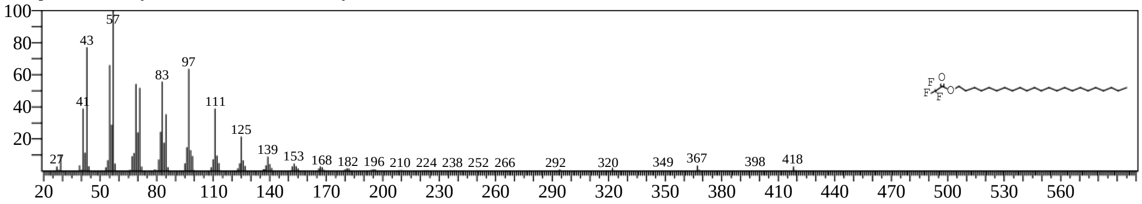

Hit#:2 Entry:35676 Library:NIST20M2.lib
SI:95 Formula:C25H43F7O2 CAS:0-00-0 MolWeight:508 RetIndex:2230
CompName:Heneicosyl heptafluorobutyrate $$ Heneicosyl 2,2,3,3,4,4,4-heptafluorobutanoate $$ 1-Heneicosanol, heptafluorobutyrate $$ Heneicosyl perflu

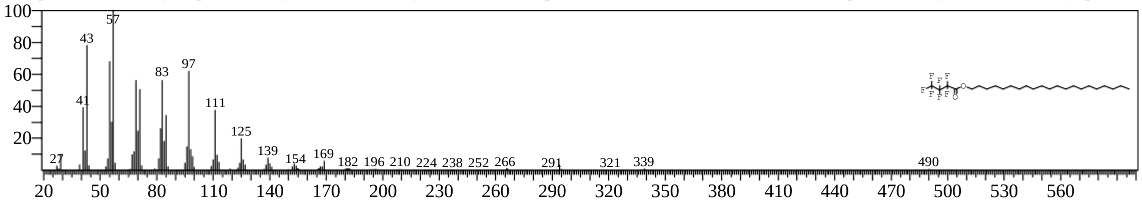

Hit#:3 Entry:20814 Library:NIST20M2.lib
SI:95 Formula:C23H41F5O2 CAS:0-00-0 MolWeight:444 RetIndex:2170
CompName:Eicosyl pentafluoropropionate $$ Eicosyl 2,2,3,3,3-pentafluoropropanoate $$ 1-Eicosanol, pentafluoropropionate

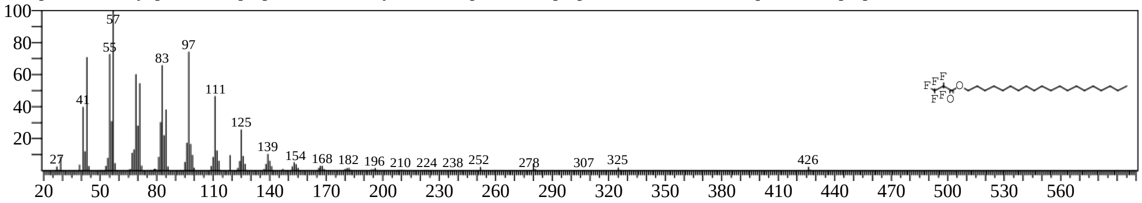

Hit#:4 Entry:11742 Library:NIST20M2.lib
SI:95 Formula:C24H45F3O2 CAS:0-00-0 MolWeight:422 RetIndex:2408
CompName:Docosyl trifluoroacetate $$ Docosyl 2,2,2-trifluoroacetate $$ 1-Docosanol, trifluoroacetate

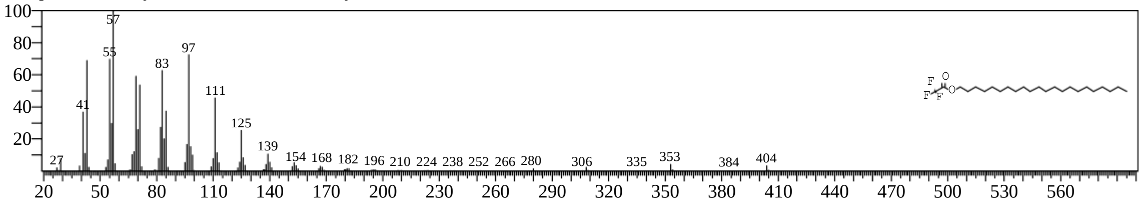

Hit#:5 Entry:234861 Library:NIST20M1.lib
SI:95 Formula:C20H37F3O2 CAS:79392-43-1 MolWeight:366 RetIndex:2011
CompName:Octadecyl trifluoroacetate $$ Octadecyl 2,2,2-trifluoroacetate $$ 1-Octadecanol, trifluoroacetate $$ Trifluoroacetic acid, n-octadecyl ester $$ A

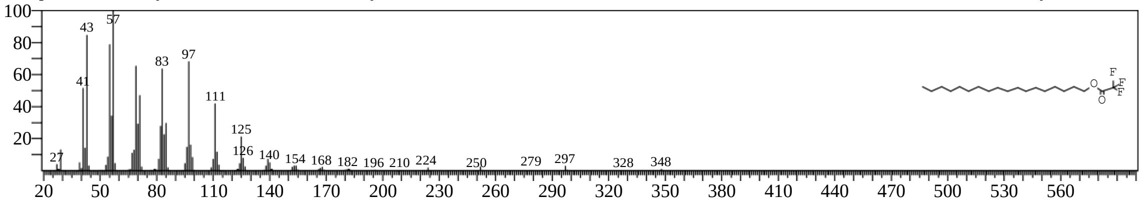

<< Target >>
Line#:20 R.Time:40.750(Scan#:2296) MassPeaks:513
RawMode:Averaged 40.733-40.767(2295-2297) BasePeak:57.10(23712)
BG Mode:Calc. from Peak Group 1 - Event 1 Scan

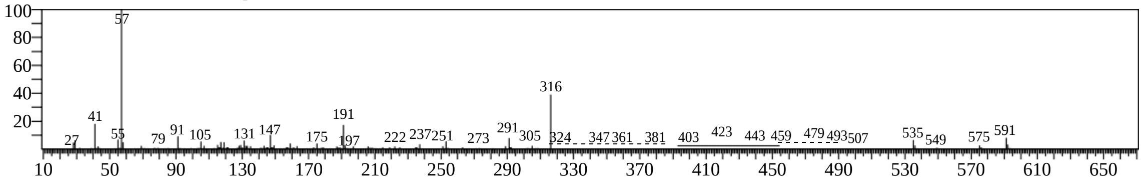

Hit#:1 Entry:44526 Library:NIST20M2.lib
SI:65 Formula:C42H63O4P CAS:95906-11-9 MolWeight:662 RetIndex:0
CompName:Tris(2,4-di-tert-butylphenyl) phosphate $$ Phenol, 2,4-bis(1,1-dimethylethyl)-, 1,1',1''-phosphate $$ Phenol, 2,4-bis(1,1-dimethylethyl)-, phosph

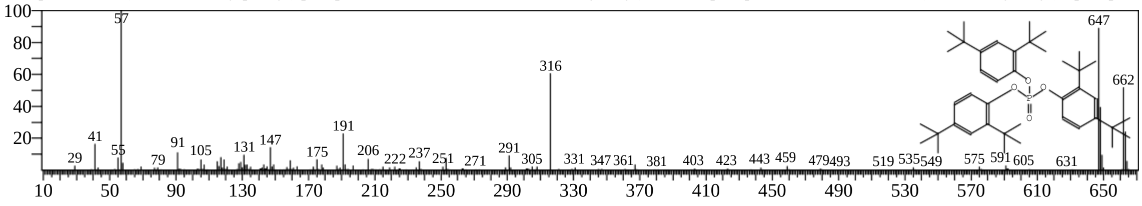

Hit#:2 Entry:42214 Library:NIST20M2.lib
SI:60 Formula:C40H58O3 CAS:134868-71-6 MolWeight:586 RetIndex:4549
CompName:2-tert-Butyl-4,6-bis(3,5-di-tert-butyl-4-hydroxybenzyl)phenol

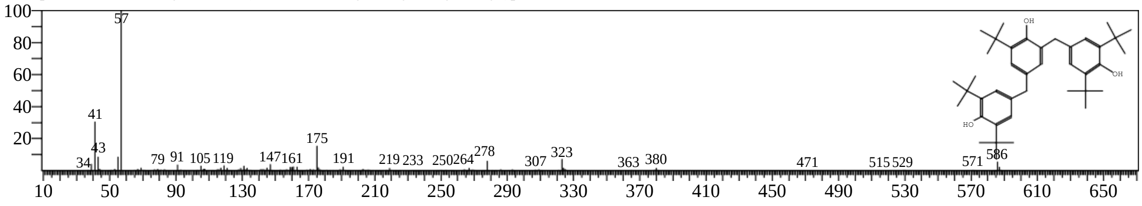

Hit#:3 Entry:182391 Library:NIST20M1.lib
SI:60 Formula:C17H23F3O2 CAS:0-00-0 MolWeight:316 RetIndex:1662
CompName:2,6-Di-tert-butyl-4-methylphenol, O-trifluoroacetyl $$ 2,6-Di-tert-butyl-4-methylphenyl 2,2,2-trifluoroacetate

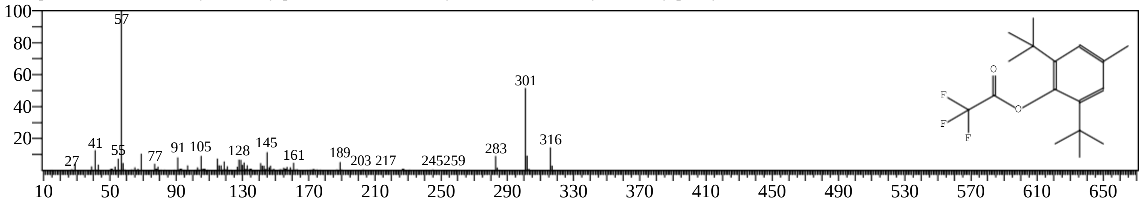

Hit#:4 Entry:43041 Library:NIST20M2.lib
SI:60 Formula:C36H49BrO3 CAS:98007-30-8 MolWeight:608 RetIndex:4542
CompName:4-Bromo-2,6-bis(3,5-di-tert-butyl-4-hydroxybenzyl)phenol

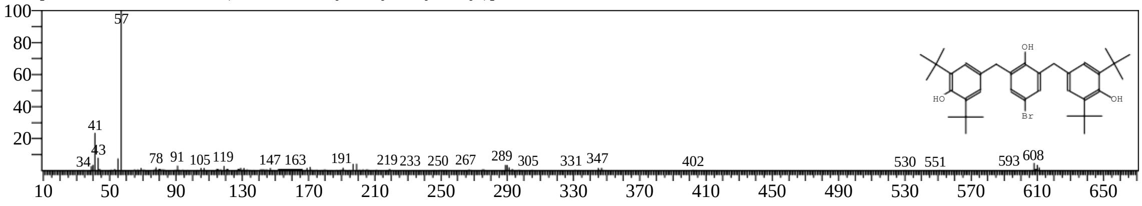

Hit#:5 Entry:214636 Library:NIST20M1.lib
SI:59 Formula:C23H36O2 CAS:56771-86-9 MolWeight:344 RetIndex:2435
CompName:1,4-Epoxynaphthalene-1(2H)-methanol, 4,5,7-tris(1,1-dimethylethyl)-3,4-dihydro-

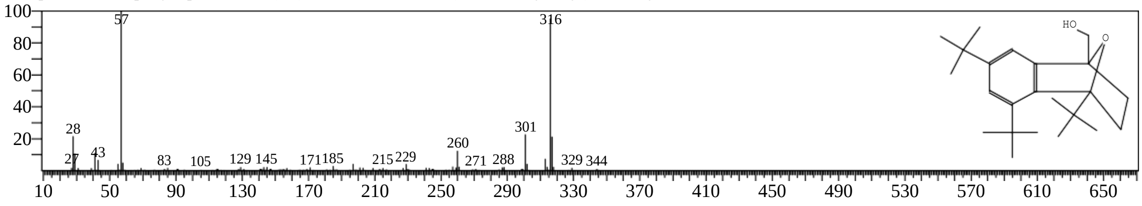

Supplement: Supplementary file 1 [file DataSheet_1.pdf]
